# Extracellular RNA clearance by non-canonical cell-surface MDA5 protects against lung fibrosis

**DOI:** 10.1038/s44321-026-00513-6

**Published:** 2026-10-12

**Authors:** Takashi Ishii, Masafumi Horie, Hirokazu Urushiyama, Yusuke Murakami, Tomoya Narita, Ryutaro Fukui, Kensuke Miyake, Akira Saito, Naomi Yamashita, Keiki Kumano, Hidenori Kage

**Affiliations:** 1 Department of Respiratory Medicine, Graduate School of Medicine, The University of Tokyo, Tokyo, Japan; 2 Department of Pharmacotherapy, Research Institute of Pharmaceutical Sciences, Musashino University, Tokyo, Japan; 3 Division for Health Service Promotion, The University of Tokyo, Tokyo, Japan; 4 Division of Molecular and Genomic Pathology, Department of Pathology, Graduate School of Medicine, Kobe University, Hyogo, Japan; 5 The Department of Computational Radiology and Preventive Medicine, The University of Tokyo, Tokyo, Japan; 6 Synergy Institute for Futuristic Mucosal Vaccine Research and Development, Chiba University, Chiba, Japan; 7 Medical Mycology Research Center, Chiba University, Chiba, Japan

**Keywords:** Immunology, Respiratory System

## Abstract

The role of innate immune receptors, specifically RNA sensors, in non-pathogen-driven lung fibrosis remains unclear. Here, we demonstrate that the cytosolic RNA sensor MDA5 could translocate to the cell surface upon bleomycin-induced damage. MDA5-deficient mice exhibited exacerbated lung fibrosis with increased IL-17A⁺ γδ T cells and neutrophils, which is independent of the canonical MAVS pathway and type I interferon signaling. Phenotypically, MDA5-deficient lung CD11b⁺ macrophages, which resemble human *SPP1*⁺ macrophages, showed elevated expression of CCR2 ligands. Mechanistically, cell-surface MDA5 acts as a scavenger receptor that captures extracellular self-RNA and shuttles it for lysosomal degradation. Loss of MDA5 leads to aberrant cellular RNA accumulation, triggering TLR8-dependent hyperproduction of CCR2 ligands. Analysis of human lung fibrosis samples from public datasets revealed *SPP1*⁺ macrophages are a major source of CCR2 ligands, suggesting their potential to activate monocytes. Our findings reveal an unrecognized protective function of MDA5 in lung fibrosis: preventing TLR8-mediated hyperinflammation through the lysosomal clearance of extracellular RNA. This mechanism provides crucial insights into the pathogenesis of fibrotic diseases like interstitial lung diseases.

## Synopsis

The innate immune receptor MDA5 plays a protective role in non-pathogen-driven lung fibrosis. By translocating to the cell surface, MDA5 clears damage-induced extracellular self-RNA via lysosomal degradation, preventing fatal hyperinflammation and fibrotic disease.

- Upon bleomycin-induced cellular damage, cytosolic MDA5 is dynamically translocated to the plasma membrane to function as a cell-surface scavenger receptor.
- Extracellular self-RNA is actively captured by surface MDA5 and shuttled into the lysosomal degradation pathway to prevent abnormal tissue accumulation.
- In MDA5-deficient models, accumulated extracellular RNA is aberrantly recognized by TLR8, whereby pathological hyperinflammation and exacerbated lung fibrosis are triggered.
- The hyperproduction of CCR2 ligands by SPP1+ macrophages and distinct IFIH1 expression in BALF cells and lungs are identified in human interstitial lung diseases.

## Abstract (web summary)

The innate immune receptor MDA5 plays a protective role in non-pathogen-driven lung fibrosis. By translocating to the cell surface, MDA5 clears damage-induced extracellular self-RNA via lysosomal degradation, preventing fatal hyperinflammation and fibrotic disease.

## Graphical abstract

**Figure:**
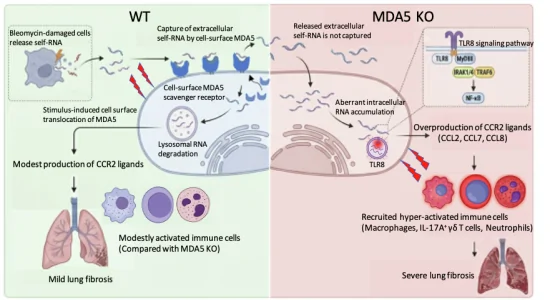

The paper explained **Problem**

Interstitial lung diseases (ILDs) are progressive, fibrotic lung conditions characterized by high mortality and limited therapeutic options. While extracellular self-nucleic acids are increasingly implicated in driving chronic inflammation, how self-RNAs are cleared from the extracellular space and whether innate immune receptors govern their homeostasis in ILD pathogenesis remain poorly understood.

**Results**

In this study, we demonstrate that the innate RNA sensor MDA5 functions as a novel scavenger device that physically captures extracellular self-RNA and shuttles it into the lysosomal degradation pathway, preventing pathological RNA accumulation. In a bleomycin-induced mouse model of pulmonary fibrosis, MDA5 deficiency markedly exacerbated lung tissue inflammation and fibrosis with excessive production of chemokines associated with CCR2 and subsequent infiltration of various types of immune cells. Mechanistically, MDA5 translocates to the cell surface to trap extracellular RNA and direct it toward lysosomal clearance; loss or knockdown of MDA5 leads to uncontrolled alternative RNA influx, overwhelming the cellular degradation machinery and triggering hyperactivation of endosomal TLR8 signaling.

**Impact**

Our findings uncover a non-canonical role for MDA5 as an extracellular RNA clearance receptor rather than merely a cytosolic innate immune sensor. Unraveling this MDA5–TLR8 regulatory axis provides a new paradigm for self-RNA homeostasis and highlights the enhancement of MDA5-mediated RNA clearance as a promising therapeutic strategy for inflammatory and fibrotic lung diseases.

## Introduction

Aberrant inflammation and subsequent profibrotic activity in various organs result in irreversible fibrotic changes, necessitating effective regulation for developing therapeutics against fibrotic diseases. Interstitial lung diseases (ILDs) are a group of fibrotic diseases, with idiopathic pulmonary fibrosis (IPF) being the most prevalent subtype. IPF often progresses rapidly despite treatment with pirfenidone and nintedanib (King et al, 2014; Ley et al, 2011; Richeldi et al, 2014). While the role of the inflammatory response in IPF remains unclear, various immune cells (including macrophages, neutrophils, and lymphocytes) and structural cells (such as fibroblasts) are known to be involved in ILD-associated fibrosis (Morse et al, 2019; Moss et al, 2022). Furthermore, patients with anti-melanoma differentiation-associated gene 5 (MDA5) antibody-positive dermatomyositis frequently experience acute ILD, which has a high mortality rate (Lu et al, 2024), suggesting the involvement of the innate immune system in ILD pathogenesis.

Innate immune receptors play a pivotal role as primary modulators in host defense against pathogens; however, their aberrant activation can induce sterile inflammation and contribute to the pathogenesis of various diseases, including autoimmune diseases, allergic diseases, and cancer (Gong et al, 2020). Some receptors specifically recognize nucleic acids, such as DNA and RtpdelNA, initiating downstream signaling involving NF-κB and interferon-regulatory factors. TLR9 and cGAS recognize DNA, whereas TLR3, TLR7, TLR8, RIG-I, and MDA5 recognize RNA (Wu and Chen, 2014). These RNA sensors primarily function to combat viral infections, and there are post-translational and transcriptional modifications that prevent the recognition of self-RNA under steady-state conditions (Chan and Gack, 2015; Rehwinkel and Gack, 2020; Schlee and Hartmann, 2016). Despite these regulatory mechanisms, self-RNA can be recognized under specific conditions, inducing downstream type I interferon signaling (Malathi et al, 2007; Stok et al, 2020). This mechanism is implicated in the pathogenesis of several diseases, such as Aicardi–Goutières syndrome, which is characterized by mutant MDA5 that is aberrantly activated by self-RNA (Rice et al, 2014). Single-nucleotide polymorphisms in *IFIH1* (gene encoding MDA5) are also associated with autoimmune and inflammatory diseases, including type 1 diabetes mellitus (Gorman et al, 2017; Smyth et al, 2006). Extracellular RNA can function as a danger-associated molecular pattern (DAMP), influencing inflammatory processes at various levels in cardiovascular and infectious diseases (Preissner et al, 2020). Thus, understanding the detailed mechanisms of how self-RNA and its receptors interact and affect various diseases is crucial. While previous studies have associated the aberrant activation of the cGAS-STING pathway with the pathogenesis of lung fibrosis (Benmerzoug et al, 2018; Zhang et al, 2022), it remains unclear whether RNA-recognizing innate immune receptors and self-RNA regulate the pathogenesis of ILDs.

In this study, we aimed to elucidate how extracellular self-RNA released by damaged cells exacerbates lung fibrosis and to determine the involvement of MDA5 in this process. MDA5 KO mice exhibited exacerbated features of bleomycin-induced lung fibrosis independent of downstream signaling through the mitochondrial antiviral signaling protein (MAVS). We found that this exacerbation of inflammation and fibrosis is linked to impaired RNA clearance. Specifically, MDA5 played a regulatory role in bleomycin-induced lung fibrosis by modulating CCR2-associated chemokines and the activation of specific subsets of macrophages, lymphocytes, and neutrophils. We also investigated the role of MDA5 in patients with ILDs and patients with anti-MDA5 autoantibody-positive dermatomyositis.

## Results

### Dynamics and distribution of MDA5 are modified by bleomycin treatment

**Figure 1:**
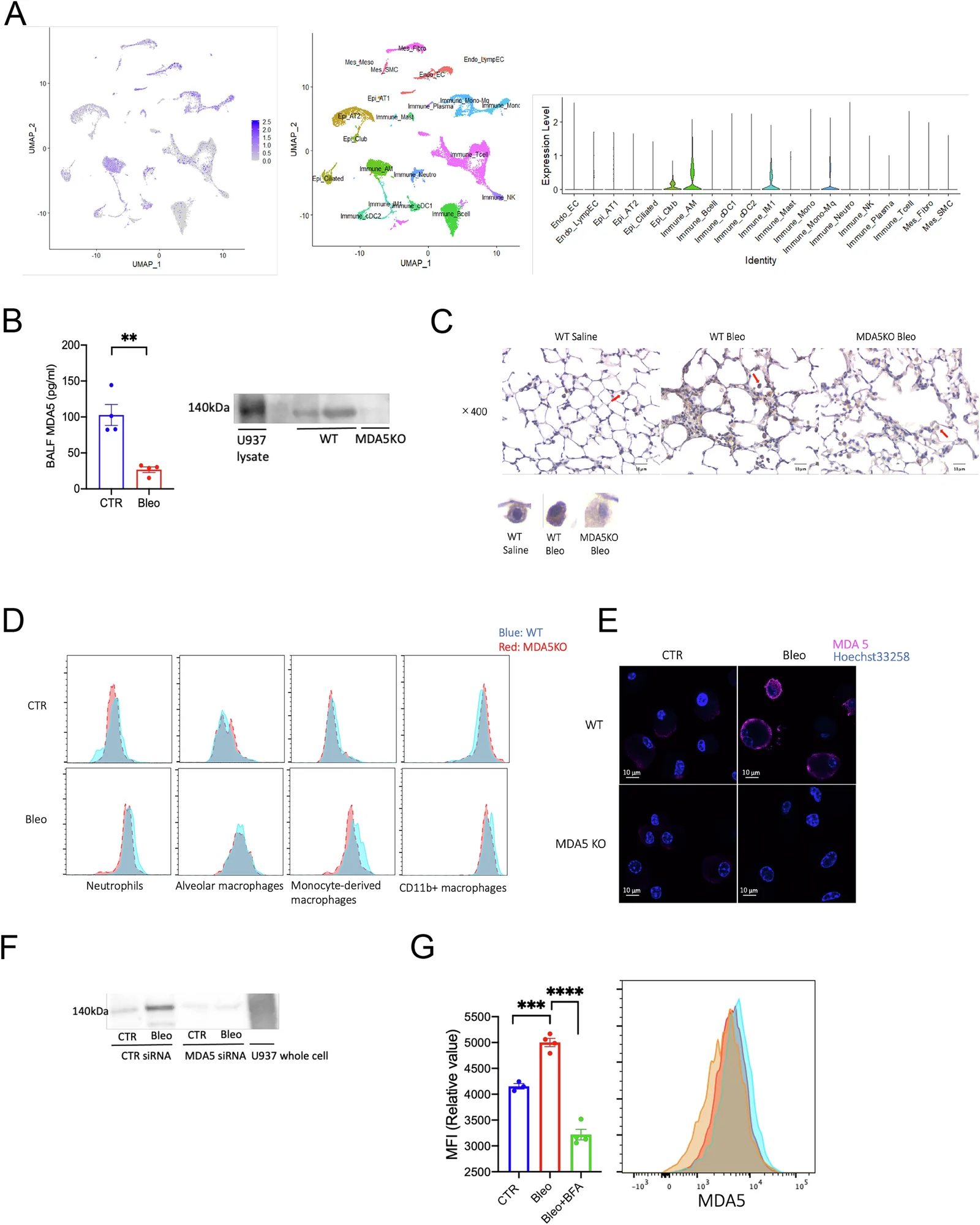
Distinct dynamics of MDA5 expression in the lung following bleomycin treatment. (**A**) *Ifih1* expression in whole lung cells derived from mice with control and radiation injury. Single-cell RNA sequencing datasets (GSE211713) were used for analysis. Upper, Uniform Manifold Approximation and Projection (UMAP) plots, lower, violin plots. Endo endothelial cells, Epi epithelial cells, Immune immune cells, Mes mesothelial cells, EC endothelial cells, LympEC lymphatic endothelial cells, AT1 alveolar type 1 cells, AT2 alveolar type 2 cells, Ciliated ciliated cells, Club club cells, AM alveolar macrophages, IM interstitial macrophages, Mast mast cells, Mono monocytes, Mq macrophages, Neutro neutrophils, NK natural killer cells, Plasma plasma cells, Fibro fibroblasts, SMC smooth muscle cells. (**B**) Protein levels of MDA5 in bronchoalveolar lavage fluid (BALF). Left: MDA5 concentration in BALF from WT mice treated with saline or bleomycin measured by ELISA, using the average measurement values from BALF of MDA5 KO mice (both under control and bleomycin-treated conditions) as background levels and subtracting them from the corresponding values of WT mice (*P* = 0.0024). Mice were analyzed two weeks after treatment. Right: Western blot analysis of concentrated BALF from WT and MDA5 KO mice to validate extracellular release. U937 lysates were used as a positive control. The data are representative of two independent experiments. (**C**) Immunohistochemical staining of lung MDA5 was performed on mice treated with either saline or bleomycin. The image of bleomycin-treated MDA5KO mice was shown as the control. Upper section, Scale bar, 20 µm; original magnification, ×400. Lower section, magnified image of representative lung macrophages. The arrow indicates MDA5 staining in a subset of macrophages. (**D**) Representative histograms of MDA5 expression in specific immune cell subsets measured by flow cytometry. Blue and red histograms represent cells from WT and MDA5 KO mice, respectively. (**E**) Immunofluorescence staining of surface MDA5 (pink) in bleomycin-treated WT bone marrow-derived macrophages (BMDMs) without permeabilization. Hoechst 33258 was used for nuclear staining (blue). Scale bar, 10 µm; original magnification, ×600. (**F**) Western blot analysis of cell surface proteins extracted from U937 cells with or without *IFIH1* knockdown treatments, treated with or without bleomycin. Data are representative of three independent experiments. (**G**) Effect of Brefeldin A (BFA) on MDA5 surface translocation in U937 cells. Left: Quantitative analysis of MDA5 mean fluorescence intensity (MFI) by flow cytometry. Right: Representative histogram of surface MDA5 expression; Red: Control, Blue: Bleomycin, Orange: Bleomycin + BFA (*P* = 0.0002 for Control vs. Bleomycin, and *P* < 0.0001 for Bleomycin vs. Bleomycin + BFA). (**B**, **G**) Data are presented as mean ± SEM. (**B**) *n* = 4 mice per group. (**G**) *n* = 3–4 per group and data are representative of two independent experiments. *P < 0.05, **P < 0.01, ***P < 0.001, ****P < 0.0001 as determined by Student’s *t* test or one-way ANOVA with Tukey’s post-hoc test. Source data are available online for this figure.

**Figure EV1:**
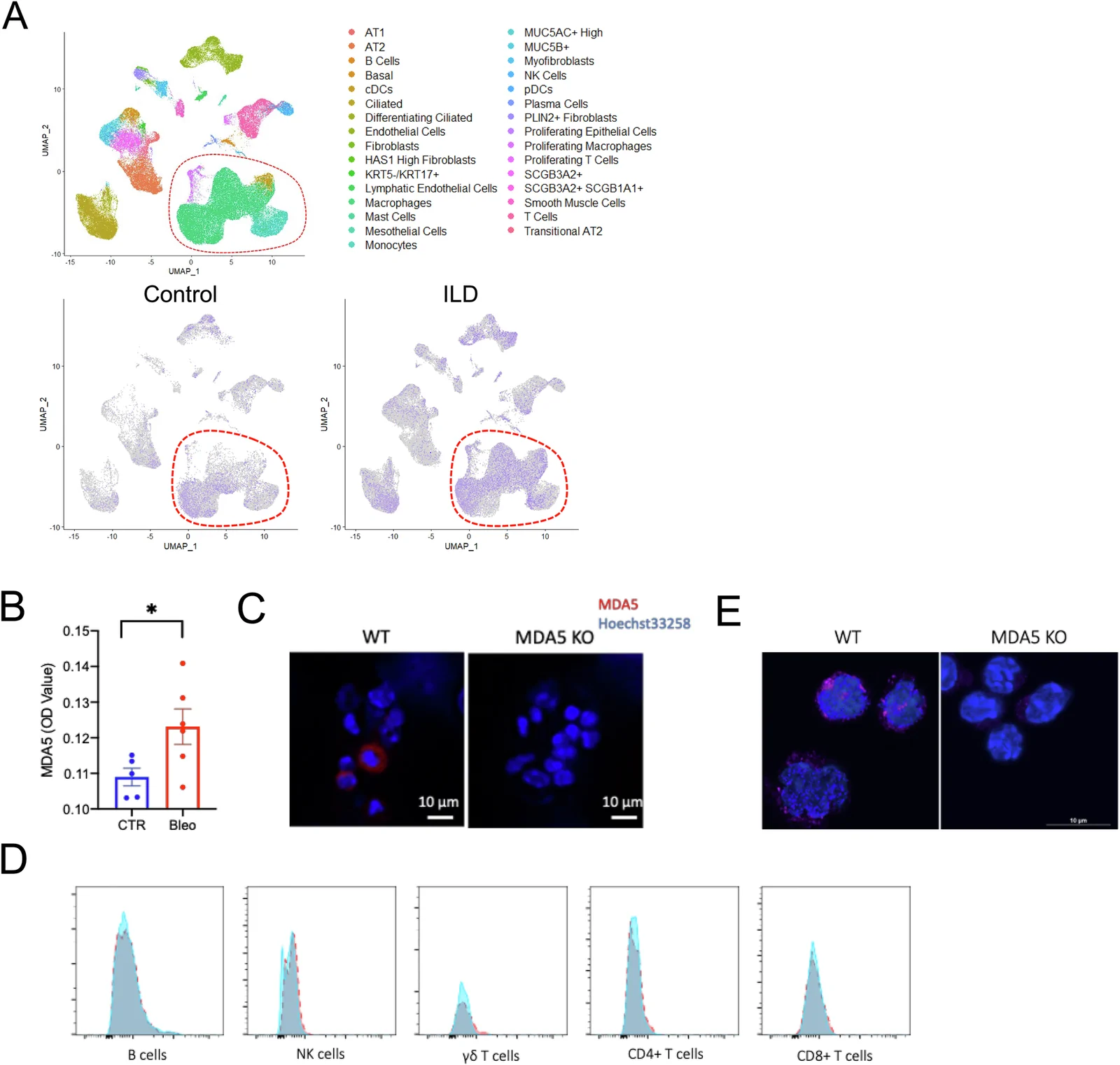
Expression and dynamics of MDA5 in lung immune cells during fibrosis. (**A**) Re-analysis of a single-cell RNA-sequencing dataset (GSE135893) from the lungs of healthy controls and patients with ILD. Upper panels show UMAP plots with new cell annotations. The red dashed circle indicates a population comprising macrophages, monocytes, and cDCs. Lower panels show the expression of *IFIH1* in this population. (**B**) U937 cells were treated with control or bleomycin for 24 h, and MDA5 levels in the supernatant were measured by ELISA (*P* value for CTR vs. Bleo: *P* = 0.0409). (**C**) Immunofluorescence staining for MDA5 (red) in single-cell suspensions from the lungs of WT and MDA5 KO mice. Nuclei were counterstained with Hoechst 33258 (blue). Scale bar, 10 µm; original magnification, ×200. (**D**) Representative flow cytometry histograms of surface MDA5 expression on lymphocyte subsets from bleomycin-treated WT (blue line) or MDA5 KO (red line, used as a negative control) mice. (**E**) Immunofluorescence staining of MDA5 (pink) in bleomycin-treated WT bone marrow-derived macrophages (BMDMs) with permeabilization using saponin. Hoechst 33258 was used for nuclear staining (blue). Scale bar, 10 µm; original magnification, ×600. Data in (**B**) is presented as mean ± SEM (*n* = 3–5 per group). *P < 0.05, by Student’s *t* test. Source data are available online for this figure

MDA5 is typically expressed in the cytoplasm and functions as an innate immune sensor for exogenous nucleic acids. We evaluated global *Ifih1* expression in the lungs of mice and humans with ILDs. In mice, re-analysis of the GSE211713 (Data ref: NCBI Gene Expression Omnibus GSE211713, 2023) radiation pneumonitis dataset revealed that *Ifih1* was predominantly expressed in alveolar, interstitial, and monocyte-derived macrophages (Fig. 1A). Single-cell RNA-seq data of lung cells derived from patients with ILDs also revealed that *IFIH1* was predominantly expressed in immune cells and endothelial cells (ECs) within the structural compartment (Fig. EV1A). To assess the dynamics and distribution of MDA5 in a bleomycin-induced mouse lung fibrosis model, we initially examined its protein levels. Interestingly, high levels of MDA5 were detected in bronchoalveolar lavage fluid (BALF), which decreased upon bleomycin treatment (Fig. 1B, left). To rigorously validate this extracellular release, we performed Western blotting on concentrated BALF; MDA5 was detected in the BALF of WT mice but was absent in that of MDA5 KO mice, confirming the specificity of the assay (Fig. 1B, right). We also measured the concentration of MDA5 in the supernatant of bleomycin- or control-treated U937 cells, revealing only a small amount of MDA5 in both conditions; however, the OD value slightly increased in the bleomycin-treated group (Fig. EV1B). Immunohistochemical staining of lung tissues for MDA5 revealed that a subset of lung macrophages, particularly in bleomycin-treated WT mice, expressed MDA5 (Fig. 1C).

Flow cytometric analysis without cell permeabilization was performed on several immune cell types from lungs derived from WT or MDA5 KO mice. This analysis revealed surface MDA5 expression on monocyte-derived macrophages and CD11b^+^ macrophages (Figs. 1D and EV1C), whereas no apparent expression of surface MDA5 was observed on lymphocytes (Fig. EV1D). Cell surface MDA5 was also detected on bleomycin-treated WT BM-derived macrophages (BMDMs) by immunofluorescence staining without permeabilization (Fig. 1E). Intracellular MDA5 expression in BMDMs, visualized via immunofluorescence staining with permeabilization, is shown in Fig. EV1E. To obtain direct biochemical evidence of this surface translocation, we isolated cell-surface proteins from U937 cells transfected with either control or *IFIH1* siRNA (Fig. EV4B), with or without subsequent bleomycin treatment. Cell viability remained high (85–95%) across all experimental groups prior to harvest. Western blot analysis demonstrated that bleomycin stimulation substantially increased the amount of MDA5 anchored to the plasma membrane (Fig. 1F). Furthermore, to elucidate the mechanism by which cytosolic MDA5 reaches the cell surface, we pre-treated the cells with Brefeldin A (BFA), an inhibitor of endoplasmic reticulum (ER)-to-Golgi transport. BFA treatment completely abolished the bleomycin-induced surface translocation of MDA5 (Fig. 1G), indicating that cellular stress actively triggers MDA5 secretion via the classical ER-to-Golgi vesicular transport pathway. These findings suggest the existence of both an extracellular and a cell-surface form of MDA5, in addition to its conventional cytoplasmic distribution. Moreover, the dynamics and distribution of MDA5 are influenced by bleomycin treatment via specific secretory mechanisms.

### MDA5 plays a protective role in bleomycin-induced lung fibrosis independent of the MDA5–MAVS pathway

**Figure 2:**
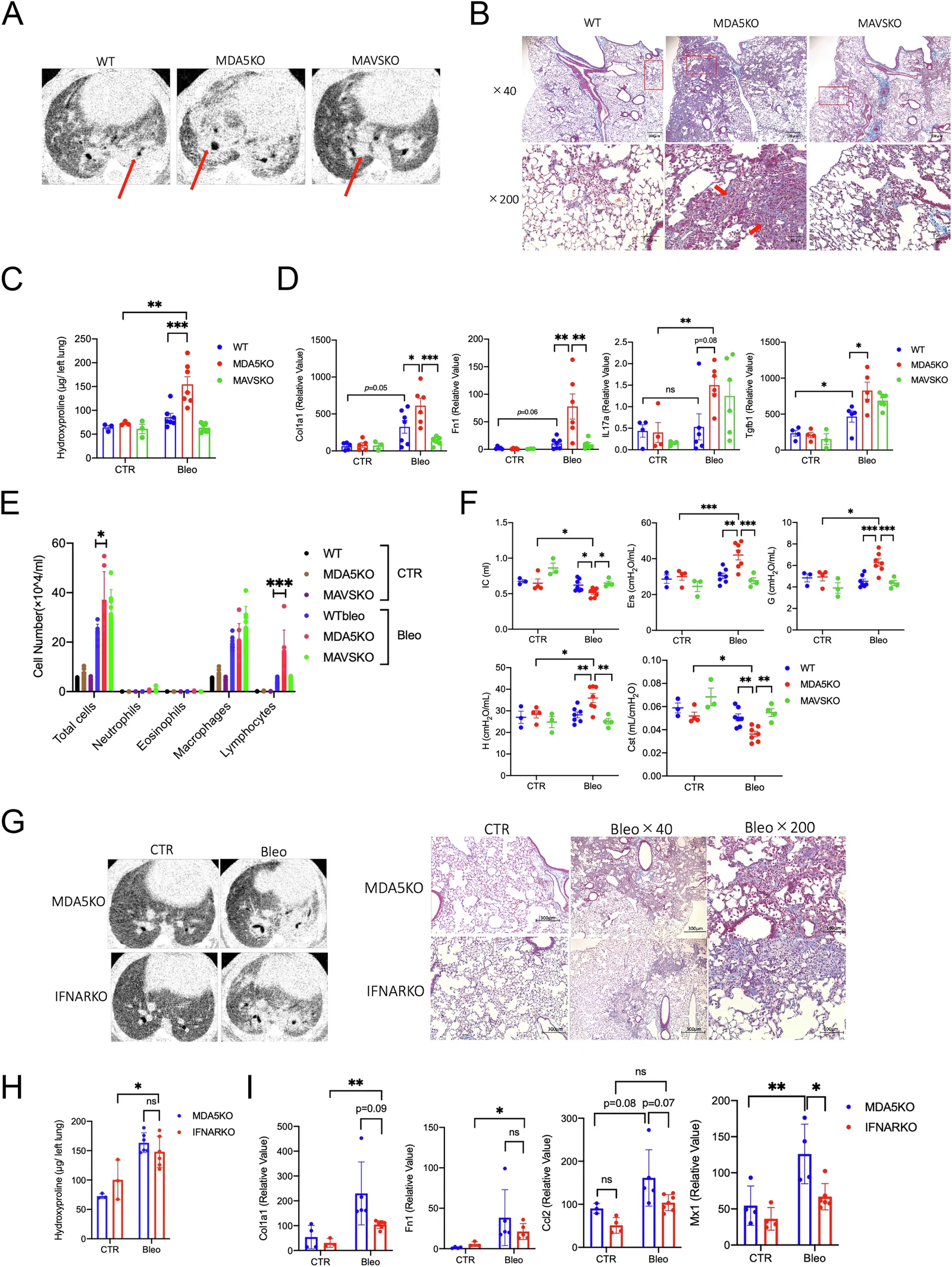
MDA5 KO mice showed more severe characteristics of interstitial pneumonia in bleomycin-induced lung fibrosis. (**A**) Representative CT images of lungs on day 21, where consolidation (arrows) reflects lung inflammation, edema, and fibrosis. (**B**) Representative lung sections of bleomycin-treated WT mice (left), MDA5 KO mice (middle), and MAVS KO mice (right). Masson’s trichrome staining was performed. Upper panels, original magnification, ×40. Lower panels, Scale bar, 50 µm; original magnification, ×200 (note that the image for WT mice in the magnification box was rotated by 90 degrees). Arrows indicate fibrotic lesions. (**C**) Measurement of hydroxyproline content in the left lung from WT, MDA5 KO, and MAVS KO mice. Measurement of hydroxyproline content in the left lung from WT, MDA5 KO, and MAVS KO mice (*P* = 0.0011 for MDA5 KO Control vs. MDA5 KO Bleomycin, and *P* = 0.0004 for WT Bleomycin vs. MDA5 KO Bleomycin). (**D**) mRNA expression levels of *Col1a1*, *Fn1*, *Tgfb1*, and *Il17a* in harvested lungs, as assessed by quantitative PCR (qPCR) analysis (*P* values for WT vs. MDA5 KO under bleomycin treatment: *Col1a1*, *P* = 0.0388; *Fn1*, *P* = 0.0028; *Tgfb1*, *P* = 0.0151. those for WT Control vs. WT Bleomycin: *Col1a1*, *p* = 0.0518; *Fn1*, *P* = 0.0553; *Tgfb1*, *P* = 0.0447. those for MDA5 KO vs. MAVS KO under bleomycin treatment: *Col1a1*, *P* = 0.0003; *Fn1*, *P* = 0.0019. *P* values for MDA5 KO CTR vs. MDA5 KO Bleomycin: *Il17a*, *P* = 0.0071). (**E**) Differential cell counts from BALF derived from WT, MDA5 KO, and MAVS KO mice (*P* values for WT vs. MDA5 KO under bleomycin treatment: total cells, *P* = 0.0322; lymphocytes, *P* = 0.0002). (**F**) Measurement of inspiratory capacity (IC), elastance (Ers), tissue damping (**G**), tissue elastance (**H**), and static compliance (Cst) as indicators of lung fibrosis using the flexiVent system (*P* values for WT vs. MDA5 KO under bleomycin treatment: IC, *P* = 0.0469; Ers, *P* = 0.0007; G, *P* = 0.0005; H, *P* = 0.0051; Cst, *P* = 0.0047. those for MDA5 KO CTR vs. MDA5 KO Bleomycin: IC, *P* = 0.033; Ers, *P* = 0.0035; G, *P* = 0.0218; H, *P* = 0.0308; Cst, *P* = 0.0133. those for MDA5 KO vs. MAVS KO under bleomycin treatment: IC, *P* = 0.0465; Ers, *P* = 0.0006; G, *P* = 0.0007; H, *P* = 0.0017; Cst, *P* = 0.0033.). (**G**–**I**) Comparison of bleomycin-induced phenotypes between MDA5 KO and IFNAR KO mice. (**G**) Left: Representative micro-CT images on day 21. Right: Representative Masson’s trichrome-stained lung sections of MDA5 KO mice (upper panels) and IFNAR KO mice (lower panels). Histology panels show saline-treated controls (CTR, left column), bleomycin-treated lungs, original magnification, ×40 (middle column), and bleomycin-treated lungs, original magnification, ×200 (right column; scale bar, 50 µm). (**H**) Measurement of hydroxyproline content in the left lung from MDA5 KO and IFNAR KO mice (*P* value for IFNAR KO CTR vs. IFNAR KO Bleomycin: *P* = 0.0448). (**I**) qPCR analysis of the expression levels of *Col1a1*, *Fn1*, *Ccl2*, and *Mx1* (*P* values for MDA5 KO vs. IFNAR KO under bleomycin treatment: *Mx1*, *P* = 0.027. those for IFNAR KO CTR vs. IFNAR KO Bleomycin: *Col1a1*, *P* = 0.0006; *Fn1*, *P* = 0.005. those for MDA5 KO CTR vs. MDA5 KO Bleomycin: *Ccl2*, *P* = 0.046; *Mx1*, *P* = 0.009). Data are presented as means ± SEM. *P < 0.05, **P < 0.01, ***P < 0.001, by two-way ANOVA with Tukey’s post-hoc test or Welch’s ANOVA followed by Dunnett’s T3 multiple comparisons test. *n* = 6–7 in the bleomycin-treated group and 3–4 in the saline-treated group (except for the flexiVent experiment, where *n* = 4–7 in the bleomycin-treated group and *n* = 3–4 in the saline-treated group). Source data are available online for this figure.

**Figure EV2:**
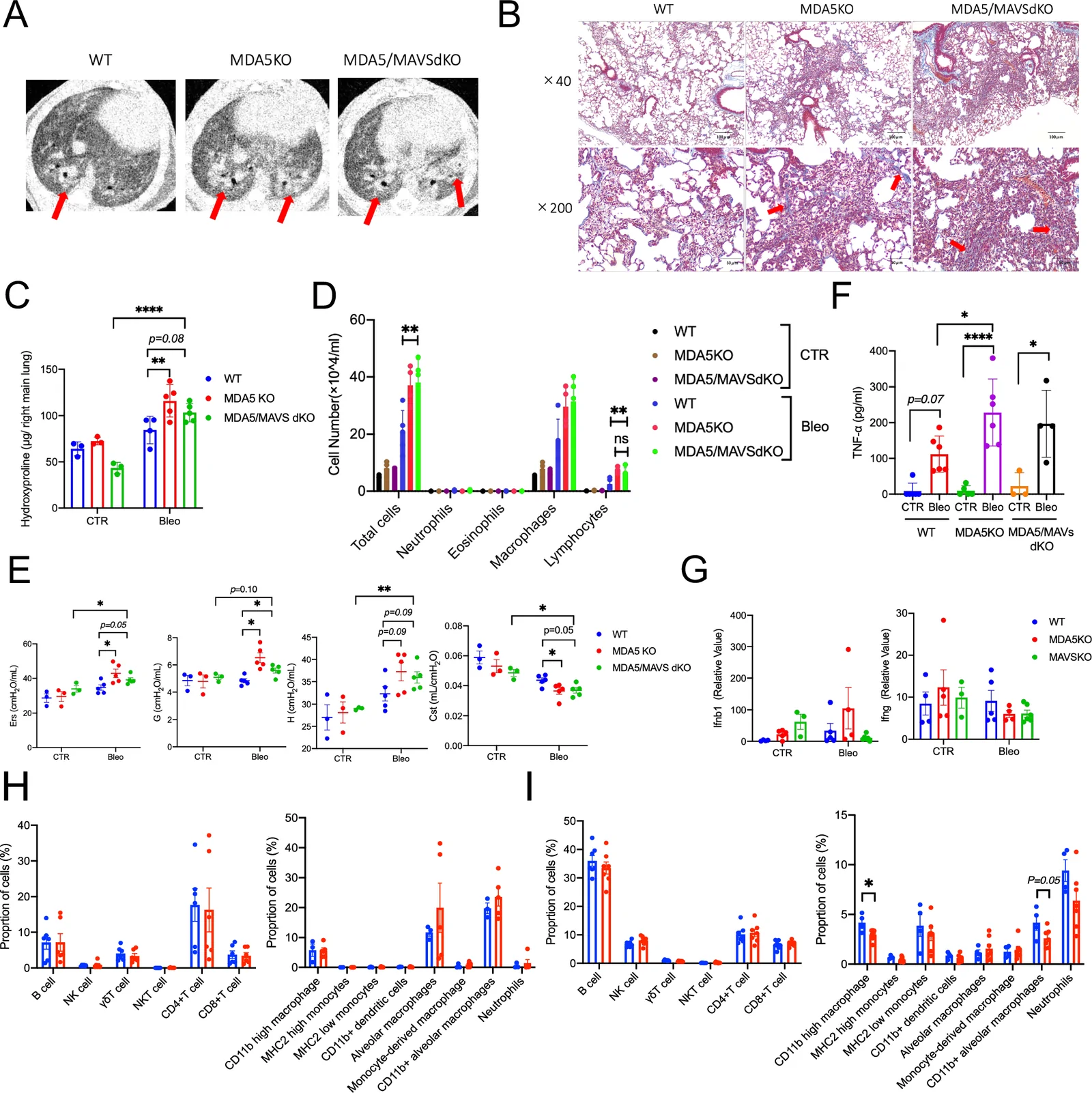
Inflammatory and fibrotic phenotype involving MDA5, MAVS, and IFNAR1. (**A**–**F**) WT, MDA5 KO, and MDA5/MAVS double KO (dKO) mice were challenged with intratracheally administered bleomycin. (**A**) Representative micro-CT images of lungs on day 21. Arrows indicate areas of consolidation reflecting inflammation, edema, and fibrosis. (**B**) Representative lung sections stained with Masson’s trichrome. Upper panels: original magnification, ×40; scale bar, 100 µm. Lower panels: original magnification, ×200; scale bar, 50 µm. (**C**) Hydroxyproline content in the right main lung (*P* values: MDA5/MAVS dKO Control vs. MDA5/MAVS dKO Bleomycin, *P* < 0.0001; WT Bleomycin vs. MDA5 KO Bleomycin, *P* = 0.0033). (**D**) Differential cell counts in BALF (*P* values for bleomycin-treated groups: total cells [WT vs. MDA5/MAVS dKO], *P* = 0.0035; lymphocytes [WT vs. MDA5/MAVS dKO], *P* = 0.003). ns not significant. (**E**) Lung mechanics, including elastance (Ers), tissue damping (G), tissue elastance (H), and static compliance (Cst), were measured using the flexiVent system. *P* values for Ers [WT Bleomycin vs. MDA5 KO Bleomycin, *P* = 0.0144; MDA5/MAVS dKO Control vs. MDA5/MAVS dKO Bleomycin, *P* = 0.0455]; G [WT Bleomycin vs. MDA5 KO Bleomycin, *P* = 0.0202; WT Bleomycin vs. MDA5/MAVS dKO Bleomycin, *P* = 0.0391]; H [WT Bleomycin vs. MDA5/MAVS dKO Bleomycin, *P* = 0.0242]; Cst [WT Bleomycin vs. MDA5 KO Bleomycin, *P* = 0.0416; MDA5/MAVS dKO Control vs. MDA5/MAVS dKO Bleomycin, *P* = 0.0242]). (**F**) TNF-α levels in the supernatants of control or bleomycin-treated alveolar macrophages derived from the indicated mice (*P* values: WT CTR vs. Bleo, *P* = 0.07; MDA5 KO CTR vs. Bleo, *P* < 0.0001; MDA5/MAVS dKO CTR vs. Bleo, *P* = 0.011; WT Bleo vs. MDA5 KO Bleo, *P* = 0.0308). (**G**) mRNA expression levels of *Ifnb1* (left) and *Ifng* (right) in whole lung homogenates from WT, MDA5 KO, and MAVS KO mice treated with saline (CTR) or bleomycin (Bleo), assessed by qPCR. (**H**, **I**) Flow cytometric analysis of lymphocyte (left) and other immune cell (right) populations in BALF (**H**) and lungs (**I**) from bleomycin-treated mice (*P* values in lungs (**I**) for MDA5 KO vs. IFNAR KO: CD11b high macrophages, *P* = 0.0195.). Blue, MDA5 KO; red, IFNAR KO. Data are presented as the mean ± SEM. *P < 0.05, **P < 0.01, ***P < 0.001, ****P < 0.0001, by one-way ANOVA with Tukey’s post-hoc test or Welch’s ANOVA followed by Dunnett’s T3 multiple comparisons test (**E**, **F**). *n* = 3–5 for the control groups, *n* = 4–7 for the bleomycin groups. Source data are available online for this figure

We compared the phenotypes of bleomycin-induced lung fibrosis in WT, MDA5 KO, and MAVS KO mice to determine whether MDA5 affects the pathogenesis of lung fibrosis and whether its effects are dependent on the canonical MDA5–MAVS pathway. Three weeks after intratracheal instillation of bleomycin, computed tomography (CT) scans revealed aggravated infiltrative shadows and ground-glass opacities, which reflect a combined landscape of active alveolar inflammation, proteinaceous edema, and progressive structural fibrosis, in MDA5 KO mice (Fig. 2A). Consistent with the CT findings, histopathological examination also revealed prominent collagen deposition and recruitment of inflammatory cells into the alveolar space in MDA5 KO mice (Fig. 2B). Furthermore, compared with WT and MAVS KO mice, MDA5 KO mice exhibited an increased amount of hydroxyproline in the lungs and significantly higher expression levels of *Col1a1, Fn1*, and *Tgfb1*, as revealed by lung quantitative PCR (qPCR) (Fig. 2C,D). We also measured the expression levels of *Ifnb* and *Ifng*, but there were no significant changes upon bleomycin treatment (Fig. EV2G). Interestingly, *Il17a* expression was increased by bleomycin treatment in MDA5 KO mice and MAVS KO mice, and showed a greater increase in bleomycin-treated MDA5 KO mice compared with WT mice (Fig. 2D). BALF analysis of differential cell counts revealed a significantly greater number of macrophages and lymphocytes following bleomycin treatment across all groups (Fig. 2E). However, lymphocyte counts in the BALF of MDA5 KO mice were significantly higher than those in the other experimental groups (Fig. 2E). Respiratory mechanics measurement showed that elastance (Ers), tissue elastance (H), and tissue damping (G) of the lungs were increased, while inspiratory capacity and static lung compliance (Cst) were decreased, only in bleomycin-treated MDA5 KO mice; these are consistent with the respiratory and physiological characteristics of interstitial pneumonia with lung fibrosis (Fig. 2F). To conclusively determine whether these MDA5-associated phenotypes are affected by the lack of MAVS signaling, we generated MDA5/MAVS double-knockout (dKO) mice. Following bleomycin treatment, dKO mice exhibited exacerbated fibrotic changes similar to those observed in MDA5 KO mice, which were significantly more severe than in their WT counterparts (Fig. EV2A–E). Furthermore, indicative of a hyper-inflammatory response, higher TNF-α secretion was observed in MDA5 KO alveolar macrophages compared with WT cells, whereas there was no significant difference between MDA5 KO and dKO alveolar macrophages (Fig. EV2F).

To investigate the involvement of the type I interferon (IFN) pathway, which could potentially be affected following MDA5 deficiency, we evaluated bleomycin-induced fibrosis in Interferon alpha receptor 1 KO (IFNAR KO) mice. While IFNAR KO mice exhibited lung fibrosis comparable to those in MDA5 KO mice in terms of CT findings (Fig. 2G), histopathological changes (Fig. 2G), hydroxyproline content (Fig. 2H), and *Fn1* expression (Fig. 2I), the expression levels of *Col1a1*, *Ccl2*, and the interferon-stimulated gene *Mx1* were suppressed (Fig. 2I). Detailed flow cytometric analysis of immune cell profiles in the BALF and lung tissues further revealed distinct inflammatory landscapes between the two strains; while no significant differences were observed in the proportions of lymphocyte lineages, the frequencies of lung CD11b^high^ macrophages and CD11b^+^ alveolar macrophages were decreased in IFNAR KO mice compared with MDA5 KO mice (Fig. EV2I). These results demonstrate that MDA5 plays a protective role against bleomycin-triggered lung fibrosis and associated inflammation via a unique mechanism that is independent of both the MDA5-MAVS and classical IFN signaling pathways.

### Distinct gene expression profiles in lymphocytes, neutrophils, and macrophages are upregulated in bleomycin-treated MDA5 KO mice

**Figure EV3:**
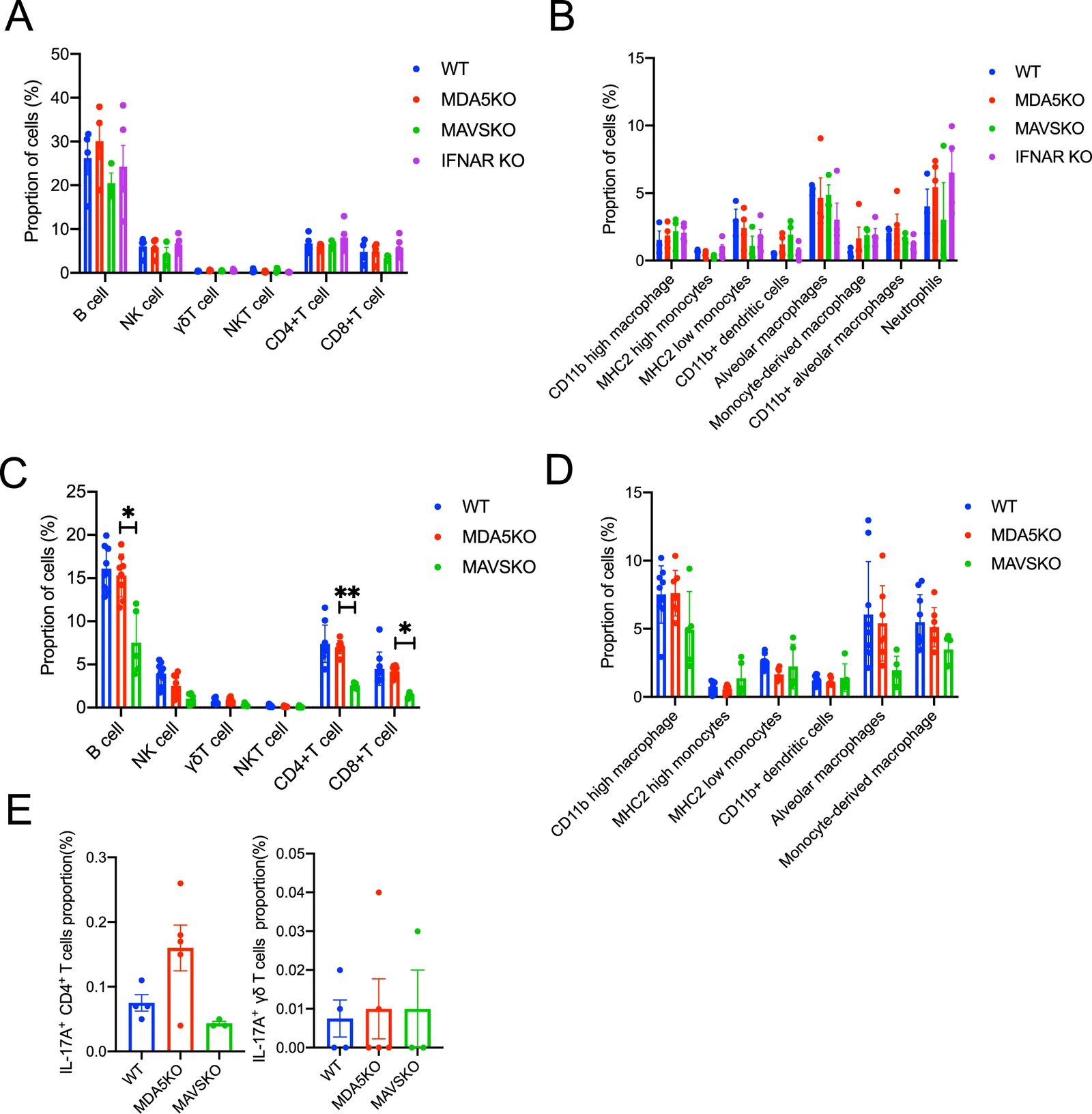
Immune cell populations in the lungs of control- and bleomycin-treated mice. (**A**, **B**) Flow cytometric analysis of lymphocyte (**A**) and other immune cell (**B**) populations in the lungs from saline-treated WT, MDA5 KO, MAVS KO, and IFNAR KO mice. (**C**, **D**) Proportions of specific lymphocyte populations (**C**) and other immune cell populations (**D**) in the lungs of bleomycin-treated WT, MDA5 KO, and MAVS KO mice, as determined by flow cytometry (exact *P* values for lymphocyte populations (**C**): B cells [MDA5 KO vs. MAVS KO], *P* < 0.0001; CD4^+^ T cells [MDA5 KO vs. MAVS KO], *P* < 0.0001; CD8^+^ T cells [MDA5 KO vs. MAVS KO], *P* = 0.0032). (**E**) The specific populations of IL-17A^+^ T cells in the lungs of saline-treated WT, MDA5 KO, and MAVS KO mice. Data are presented as mean ± SEM. *P < 0.05, **P < 0.01, ***P < 0.001, ****P < 0.0001, by two-way ANOVA with Tukey’s post-hoc test. *n* = 3–8 per group. Source data are available online for this figure

**Figure 3:**
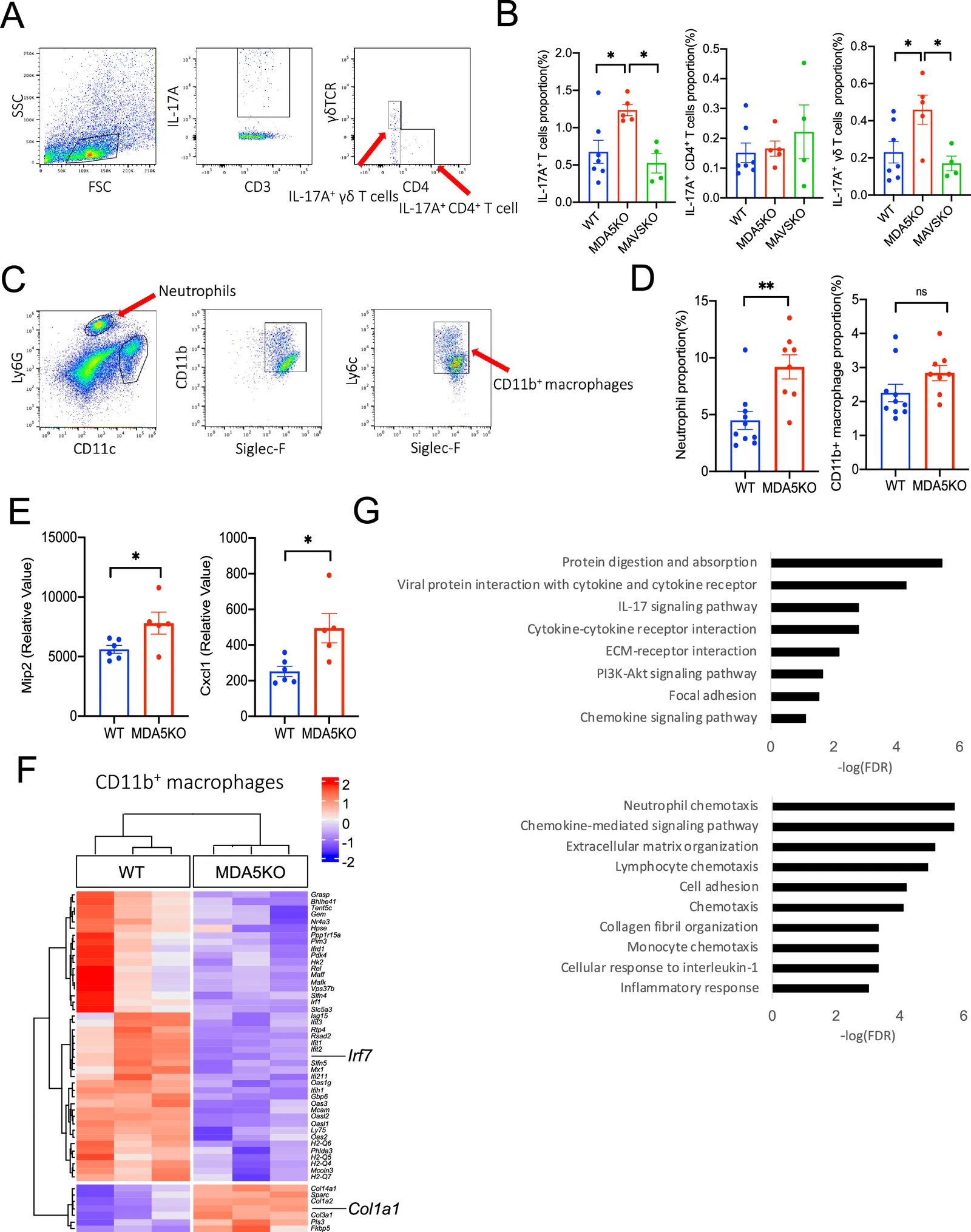
An increased proportion of lung IL-17A^+^ T cells and neutrophils, and distinct expression profiles in lung CD11b^+^ macrophages in bleomycin-treated MDA5 KO mice. (**A**) Gating strategy for identifying IL-17A-producing T cells in lung single-cell suspensions by flow cytometry. (**B**) Proportion of IL-17A^+^ T cells (left), IL-17A^+^ CD4^+^ T cells (middle), and IL-17A^+^ γδ T cells (right) in the lung derived from bleomycin-treated WT or MDA5KO mice (*P* values for WT vs. MDA5 KO: IL-17A^+^ T cells, *P* = 0.0262; IL-17A^+^ γδ T cells, *P* = 0.0487; those for MDA5 KO vs. MAVS KO: IL-17A^+^ T cells, *P* = 0.0141; IL-17A^+^ γδ T cells, *P* = 0.0487). (**C**) Isolation of neutrophils and CD11b^+^ macrophages sorted from the lungs of bleomycin-treated WT or MDA5 KO mice. (**D**) Proportion of lung neutrophils (left) and CD11b^+^ macrophages (right) in bleomycin-treated WT or MDA5 KO mice (*P* values for WT vs. MDA5 KO: neutrophils, *P* = 0.0022). (**E**) Expression levels of *Mip2* and *Cxcl1* in lung neutrophils measured by qPCR (*P* values for WT vs. MDA5 KO: *Mip2*, *P* = 0.0385; *Cxcl1*, *P* = 0.0145). (**F**, **G**) Global gene expression analysis in lung CD11b^+^ macrophages in bleomycin-treated WT or MDA5 KO mice by RNA-seq. (**F**) Heatmap of the top 50 DEGs in RNA harvested from CD11b^+^ macrophages derived from bleomycin-treated WT or MDA5 KO mice. (**G**) Gene ontology terms of biological processes (upper) and pathway terms (lower) determined by the gene set (fold change ≥1.5, FDR < 0.05) upregulated in MDA5 KO-derived CD11b^+^ macrophages. Data are presented as means ± SEM. *P < 0.05, **P < 0.01, by one-way ANOVA with Tukey’s post-hoc test. *n* = 4–6 in each group (**A**–**E**). Source data are available online for this figure.

In bleomycin-treated MDA5 KO mice, the number of lymphocytes in BALF and *Il17a* expression in the lungs were increased compared with those in the other experimental groups (Fig. 2D,E). Flow cytometric analysis was conducted to investigate the differential lymphocyte counts in the lungs of bleomycin-treated mice. We confirmed that the baseline cell proportions of both lymphocytes and other representative immune cells in the lungs exhibited no significant differences among WT, MDA5 KO, MAVS KO, and IFNAR KO mice (Fig. EV3A,B). Furthermore, there were no significant differences in the baseline proportions of specific lymphocyte subsets between WT and MDA5 KO mice (Fig. EV3C). IL-17A production by lymphocytes was then assessed through intracellular staining (Fig. 3A). The population of lung IL-17A-producing T cells, particularly IL-17A-producing γδ T cells, was significantly increased in bleomycin-treated MDA5 KO mice compared with WT mice (Fig. 3B), whereas their proportions showed no significant differences between the control mice (Fig. EV3E).

Subsequently, we evaluated other immune cells, including neutrophils, monocytes, and macrophages, in the lungs after bleomycin treatment. However, flow cytometric analysis revealed no substantial changes in the proportions of monocytes and macrophages between WT and MDA5 KO mice (Fig. EV3D). Considering the elevated expression of *Il17a in* lung T cells (Fig. 3A,B) and *Ifih1* in CD11b^+^ macrophages as demonstrated in public immune cell databases, we further analyzed lung neutrophils and CD11b^+^ macrophages (Fig. 3C). The proportion of lung neutrophils was increased, and their expression of *Cxcl1* and *Mip2*, which are indicators of chemotaxis and activation of neutrophils, was elevated in MDA5 KO mice compared with WT mice (Fig. 3D,E).

Regarding CD11b^+^ macrophages, although no significant difference in their proportion was observed between WT and MDA5 KO mice (Fig. 3D), RNA-seq analysis revealed distinct gene expression profiles between the two groups. The heatmap of the top 50 differentially expressed genes (DEGs) revealed elevated expression of fibrosis-related genes, including *Col1a1, Col1a2, Col3a1, Col14a1*, and *Sparc*, in the CD11b^+^ macrophages of MDA5 KO mice, whereas the expression of interferon-stimulated genes and the interferon-regulatory factor family was elevated in those of WT mice (Fig. 3F). Pathway and Gene Ontology (GO) analyses of the DEGs upregulated in MDA5 KO CD11b^+^ macrophages revealed several terms related to inflammation, chemotaxis, and cytokine interaction, including IL-17A signaling and tissue fibrosis (Fig. 3G). In summary, the populations of IL-17A-producing γδ T cells and subsequent inflammatory lung neutrophils were increased, and the transcriptional profile of CD11b^+^ macrophages favored chemotaxis, inflammation, and fibrosis in the lungs of bleomycin-treated MDA5 KO mice compared with their WT counterparts.

### Chemokines mediating CCR2 signaling in macrophages as key drivers of MDA5-dependent aggravation of bleomycin-induced lung fibrosis

**Figure 4:**
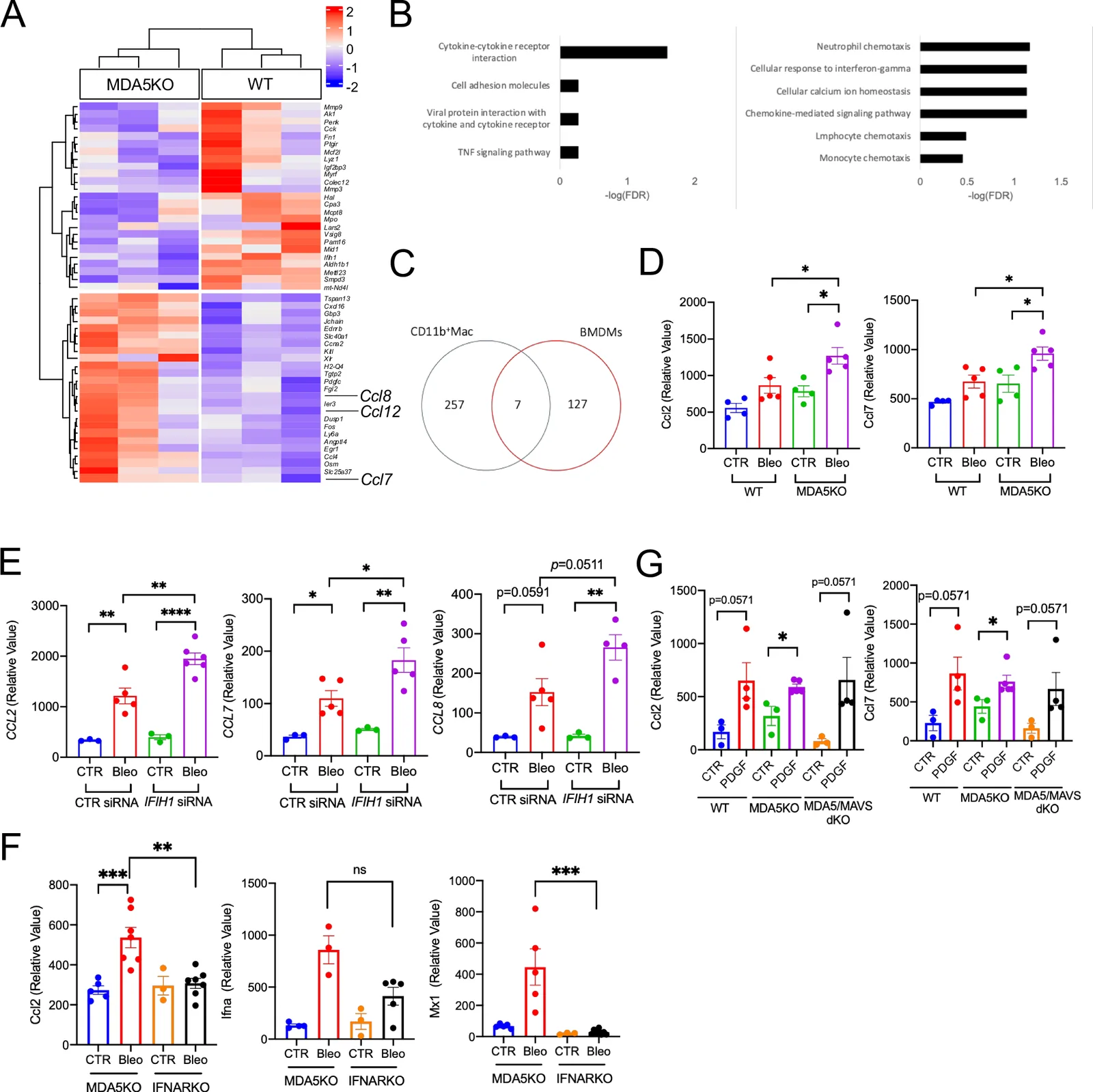
MDA5 regulates bleomycin-associated inflammation mediated by chemokines particularly those related to CCR2 in BMDMs and U937 cells. (**A**–**D**) BMDMs were harvested from WT or MDA5 KO mice and treated with control or bleomycin for subsequent analysis. (**A**, **B**) Global gene expression analysis in BMDMs. (**A**) A heatmap of the top 50 DEGs (*P* < 0.05) in harvested RNA of bleomycin-treated WT or MDA5 KO BMDMs. (**B**) Gene ontology terms of biological processes (upper) and pathway terms (lower) determined by the gene sets upregulated (*P* < 0.05) in MDA5 KO BMDMs. (**C**) Venn diagram showing the overlap between genes upregulated in MDA5 KO CD11b⁺ macrophages and those upregulated in MDA5 KO BMDMs, compared with their respective WT counterparts. The number of genes in each category is indicated. (**D**) The expression levels of chemokines related to CCR2 in BMDMs were assessed by qPCR (*Ccl2*, left, *Ccl7*, right) (*P* values for WT vs. MDA5 KO under bleomycin treatment: *Ccl2*, *P* = 0.0366; *Ccl7*, *P* = 0.0277. *P* values for MDA5 KO CTR vs. MDA5 KO Bleomycin: *Ccl2*, *P* = 0.0174; *Ccl7*, *P* = 0.0252). (**E**) U937 cells were transfected with control or MDA5 siRNA for knockdown of *IFIH1*, followed by treatment with control or bleomycin. The expression levels of chemokines related to CCR2 (*CCL2*, left, *CCL7*, middle, *CCL8*, right) were measured by qPCR (*P* values for control siRNA vs. *IFIH1* siRNA under bleomycin treatment: *CCL2*, *P* = 0.0024; *CCL7*, *P* = 0.0292. those for control siRNA CTR vs. control siRNA Bleomycin: *CCL2*, *P* = 0.0026; *CCL7*, *P* = 0.0485. those for *IFIH1* siRNA CTR vs. *IFIH1* siRNA Bleomycin: *CCL2*, *P* < 0.0001; *CCL7*, *P* = 0.0014; *CCL8*, *P* = 0.0018). (**F**) The expression levels of *Ccl2* and *Mx1* in BMDMs from MDA5 KO and IFNAR KO mice treated with control or bleomycin were assessed by qPCR (*P* values for MDA5 KO vs. IFNAR KO under bleomycin treatment: *Ccl2*, *P* = 0.0007; *Mx1*, *P* = 0.0001. Those for MDA5 KO CTR vs MDA5 KO Bleomycin: *Ccl2*, *P* = 0.0012). (**G**) BMDMs from WT, MDA5 KO, and MDA5/MAVS double KO (dKO) mice were stimulated with control (CTR) or PDGF-BB (PDGF) for 6 h, and the expression levels of *Ccl2* and *Ccl7* were measured by qPCR (*P* values for PDGF stimulation effects MDA5 KO: *Ccl2*, *P* = 0.0357; *Ccl7*, *P* = 0.0357). Data are presented as means ± SEM. *P < 0.05, *P* < 0.01, ***P < 0.001, ns, not significant, by one-way ANOVA with Tukey’s post-hoc test or Welch’s ANOVA followed by Dunnett’s T3 multiple comparisons test (**E**–**G**). (**D**–**G**) *n* = 4–6 in each group. Source data are available online for this figure.

**Figure EV4:**
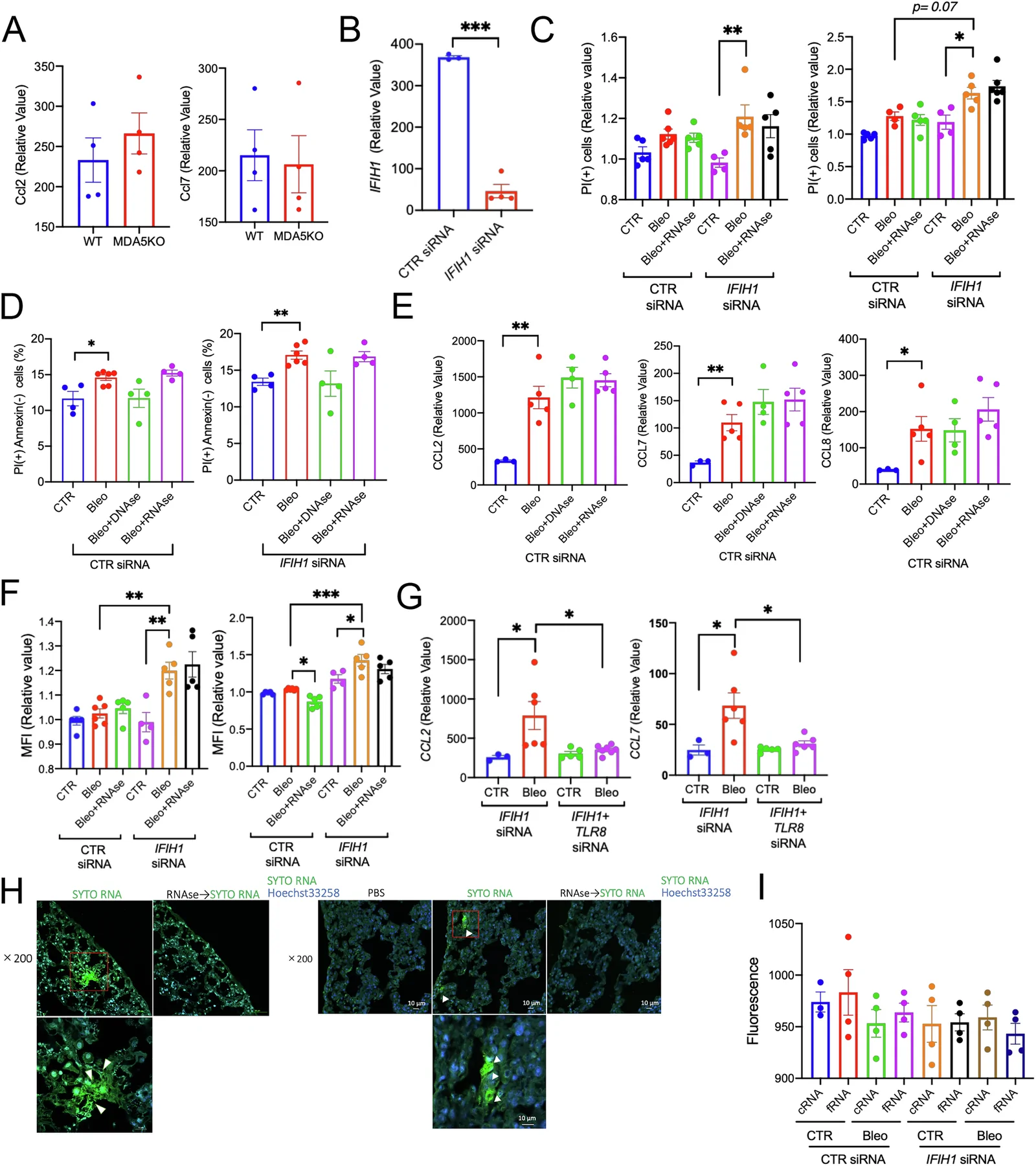
In vitro validation of the MDA5–RNA–TLR8 axis. (**A**) mRNA expression levels of *Ccl2* and *Ccl7* in PBS-treated BMDMs, assessed by qPCR. (**B**) *IFIH1* mRNA expression in U937 cells 24 h after transfection with control or *IFIH1* siRNA, measured by qPCR (*P* < 0.0001). (**C**) Proportions of PI-positive (necrotic) U937 cells 24 h (left) and 48 h (right) after stimulation. Cells were pre-treated with control or *IFIH1* siRNA, and then stimulated with PBS, bleomycin, or bleomycin + RNase (*P* value at 24 h: *IFIH1* siRNA [Control vs. Bleomycin], *P* = 0.0090; and similarly at 48 h: *IFIH1* siRNA [Control vs. Bleomycin], *P* = 0.0133). (**D**) U937 cells with or without *IFIH1* knockdown were treated with control, bleomycin, bleomycin + DNase, or bleomycin + RNase. The proportions of necrotic cells (Annexin-V^−^ PI^+^) were evaluated (*P* values: CTR siRNA [Control vs. Bleomycin], *P* = 0.0178; *IFIH1* siRNA [Control vs. Bleomycin], *P* = 0.0034). (**E**) mRNA expression levels of *CCL2*, *CCL7*, and *CCL8* in U937 cells stimulated with PBS, bleomycin, bleomycin + DNase, or bleomycin + RNase (*P* values: *CCL2* [Control vs. Bleomycin, *P* = 0.0025]; *CCL7* [Control vs. Bleomycin, *P* = 0.0086]; *CCL8* [Control vs. Bleomycin, *P* = 0.0299]). (**F**) Cellular RNA levels in U937 cells, measured by flow cytometry. MFI of an RNA-specific dye is shown for PI-negative (live, left) and PI-positive (necrotic, right) cells 24 h post-stimulation, as in (**C**) (*P* values for live cells: *IFIH1* siRNA [Control vs. Bleomycin], *P* = 0.0024; Bleomycin [CTR siRNA vs. *IFIH1* siRNA], *p* = 0.0056. *P* values for necrotic cells: CTR siRNA [Bleomycin vs. Bleomycin + RNase], *P* = 0.0001; *IFIH1* siRNA [Control vs. Bleomycin], *P* = 0.0367; Bleomycin [CTR siRNA vs. *IFIH1* siRNA], *P* < 0.0001). (**G**) mRNA expression levels of *CCL2* (left) and *CCL7* (right) in U937 cells transfected with control, *IFIH1* siRNA, or *IFIH1* + *TLR8* siRNAs, and then stimulated with bleomycin (*P* values for *IFIH1* siRNA Control vs. *IFIH1* siRNA Bleomycin: *CCL2*, *P* = 0.0303; *CCL7*, *P* = 0.0156. Those for *IFIH1* siRNA Bleomycin vs. *IFIH1* + *TLR8* siRNA Bleomycin: *CCL2*, *P* = 0.0232; *CCL7*, *P* = 0.0108). (**H**) Left: High-magnification images of RNA staining in the bleomycin-treated lungs of MDA5 KO mice shown in Fig. 6F. Upper panels, original magnification, ×200. Lower panel, original magnification, ×600; scale bar, 10 µm. Right: RNA staining with SYTO RNAselect in lung tissues from WT mice treated with bleomycin (0.5 mg/kg) to induce severe lung fibrosis. Hoechst 33258 was used for nuclear staining. Images show treatments with PBS (left), SYTO RNA (middle), and SYTO RNA after RNase treatment (right). Upper panels, original magnification, ×200. Lower panel (SYTO RNA), original magnification, ×600; scale bar, 10 µm. Arrows indicate RNA depositions. (**I**) Fluorescence in the supernatants of U937 cells treated with control RNA (cRNA) or fluorescent RNA (fRNA) were measured. Data are presented as mean ± SEM. *P < 0.05, *P* < 0.01, ***P < 0.001, by Student’s *t* test or one-way ANOVA. *n* = 3–6 per group. Source data are available online for this figure

To further evaluate the role of macrophages in the distinct phenotypes of lung fibrosis between WT and MDA5 KO mice, we cultured BMDMs from WT or MDA5 KO mice and treated them with bleomycin. RNA-seq analysis of these BMDMs revealed a distinct profile, and a heatmap of the top 50 DEGs was obtained (Fig. 4A). Among the upregulated DEGs in MDA5 KO BMDMs, *Ccl7, Ccl8*, and *Ccl12* were identified, and pathway and GO analyses highlighted chemokine-mediated signaling, chemotaxis, and cytokine-cytokine receptor interaction (Fig. 4B). By cross-referencing the RNA-seq results from CD11b^+^ lung macrophages and BMDMs, seven genes were commonly identified as upregulated DEGs in MDA5 KO mice compared with WT mice (Fig. 4C). Notably, *Ccl2, Ccl7*, and *Ccl12*, which are ligands for the chemokine receptor CCR2, were included. qPCR analysis confirmed that the expression levels of *Ccl2* and *Ccl7* were also significantly elevated in BMDMs treated with bleomycin, and this elevation was more pronounced in bleomycin-treated MDA5 KO BMDMs compared with their WT counterparts. In contrast, baseline expression levels of *Ccl2* and *Ccl7* were comparable between BMDMs from both strains (Figs. 4D and EV4A). To validate these findings in human cells, we conducted siRNA experiments using the human monocyte cell line U937 to knock down *IFIH1* expression (Fig. EV4B). qPCR analysis showed increased expression of *CCL2, CCL7*, and *CCL8* (ligands for human CCR2) in U937 cells following bleomycin treatment. In addition, *IFIH1* knockdown in U937 cells further enhanced the expression of these chemokines post-bleomycin treatment compared with cells treated with control siRNA (Fig. 4E). This hyper-inflammatory response was further confirmed in primary alveolar macrophages, where bleomycin stimulation induced higher TNF-α secretion in MDA5 KO alveolar macrophages compared with WT cells (Fig. EV2F).

Importantly, these findings suggest that this exaggerated chemokine response cannot be fully explained solely by the attenuation of classical type I interferon signaling, given that the induction of *Ccl2* and *Mx1* was significantly blunted in bleomycin-treated IFNAR KO BMDMs (Fig. 4F). Furthermore, to determine if this response is specific to bleomycin-induced cellular damage rather than general pro-fibrotic signaling, we stimulated BMDMs with PDGF-BB. Unlike bleomycin, PDGF-BB did not elicit differences in *Ccl2* or *Ccl7* expression between WT and MDA5 KO cells, highlighting that the MDA5-dependent protective mechanism is specifically triggered by damage-associated stimuli (Fig. 4G).

**Figure 5:**
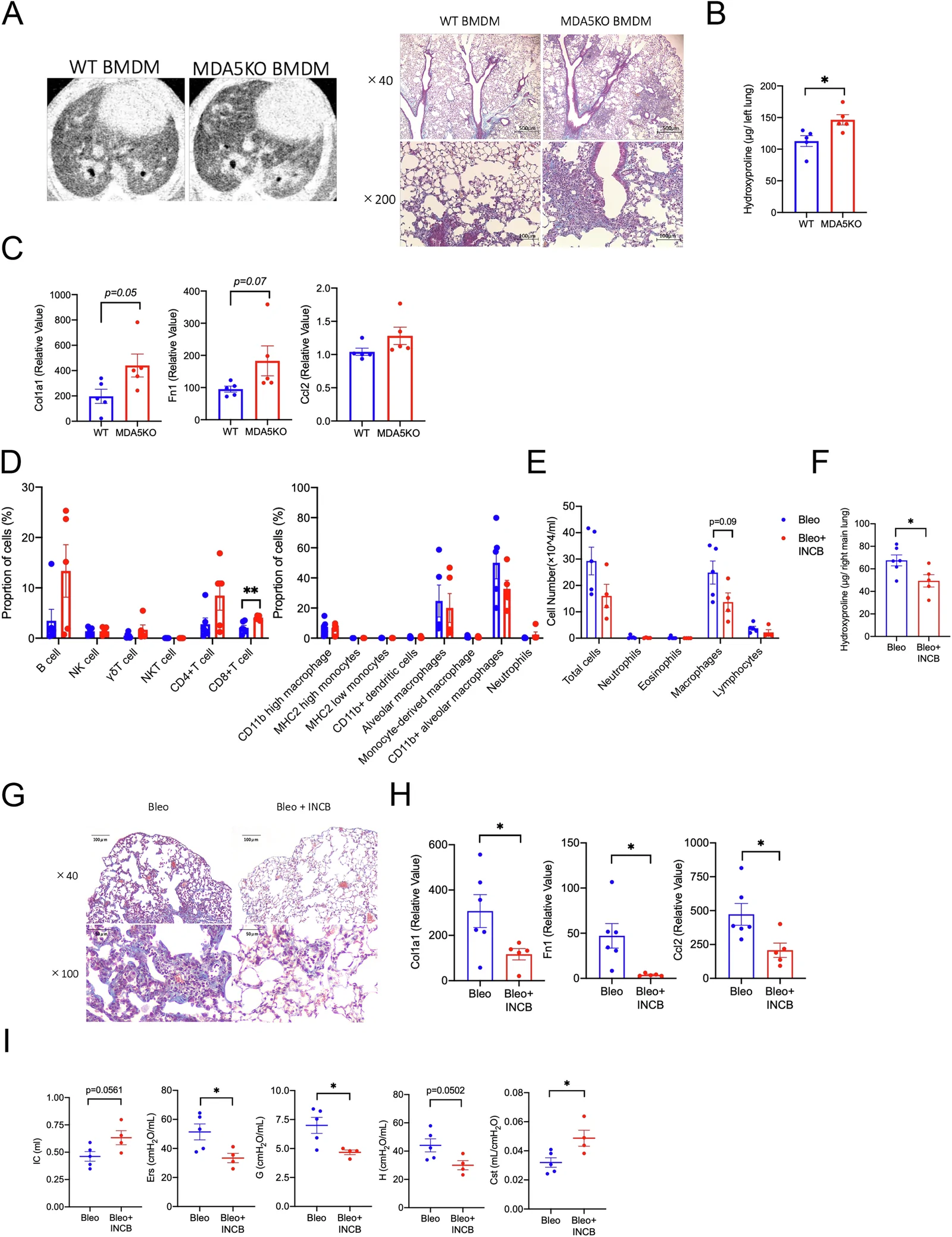
Macrophages drive exacerbated lung fibrosis in MDA5 KO mice, and a CCR2 antagonist ameliorates the pathogenesis. (**A**–**D**) WT (*n* = 5) or MDA5 KO (*n* = 5) BMDMs were stimulated with bleomycin and intratracheally transferred into recipient WT mice alongside a low dose of bleomycin. After two weeks, lungs and BALF were harvested. (**A**) Representative CT images (left) and histological analysis (right; Masson’s trichrome staining: upper panel, original magnification, ×40; lower panel, original magnification, ×100. Scale bar, 50 µm). (**B**) Measurement of hydroxyproline content (*P* value for WT BMDM vs. MDA5 KO BMDM: *P* = 0.0218), and (**C**) mRNA expression levels of *Col1a1*, *Fn1*, and *Ccl2* in harvested lungs. (**D**) Flow cytometric analysis of lymphocyte (left) and other immune cell (right) populations in the BALF (*P* values for WT BMDM vs. MDA5 KO BMDM: lymphocytes, *P* = 0.0087). (**E**–**I**) Bleomycin-treated MDA5 KO mice were intraperitoneally administered either the CCR2 antagonist INCB3344 or vehicle (DMSO), as described in the Methods. (**E**) Differential cell counts from BALF in each group. (**F**) Measurement of the amount of hydroxyproline in the right main lung of each mouse (*P* value for DMSO vs. INCB3344: *P* = 0.0343). (**G**) Representative lung sections of DMSO-treated mice (left) and INCB3344-treated mice (right). Masson’s trichrome staining: upper panel, original magnification, ×40; lower panel, original magnification, ×100. Scale bar, 50 µm. (**H**) mRNA expression levels of *Col1a1*, *Fn1*, and *Ccl2* in harvested lungs, as assessed by qPCR (exact *P* values for DMSO vs. INCB3344: *Col1a1*, *P* = 0.0483; *Fn1*, *P* = 0.0184; *Ccl2*, *P* = 0.0276). (**I**) Inspiratory capacity (IC), elastance (Ers), tissue damping (**G**), tissue elastance (**H**), and static compliance (Cst) as indicators of lung fibrosis were measured using a flexiVent system (exact *P* values for DMSO vs. INCB3344: Ers, *P* = 0.0335; G, *P* = 0.0232; Cst, *P* = 0.0287). Data are presented as means ± SEM. *P < 0.05, as determined by Student’s *t* test. *n* = 6 in the bleomycin+DMSO-treated group and *n* = 5 in the bleomycin+INCB3344-treated group. Source data are available online for this figure.

To directly demonstrate that macrophages are the primary cellular drivers of the exacerbated fibrotic phenotype in MDA5 KO mice, we performed an in vivo adoptive transfer experiment. WT or MDA5 KO BMDMs were stimulated with bleomycin and intratracheally transferred into recipient WT mice alongside only a low dose of bleomycin. Notably, mice receiving MDA5 KO BMDMs exhibited exacerbated inflammation and fibrosis compared with those receiving WT BMDMs, as evidenced by CT imaging, histological analysis, the hydroxyproline assay, and lung qPCR (Fig. 5A–C). Furthermore, flow cytometric analysis detected a significant increase in CD8^+^ T cell populations in the BALF of mice transplanted with MDA5 KO BMDMs (Fig. 5D).

Given the established role of CCR2 ligands in lung fibrosis, we hypothesized that blocking CCR2 might attenuate the MDA5-associated aggravation of lung fibrosis. We administered the CCR2 antagonist INCB3344 or vehicle to bleomycin-treated MDA5 KO mice and evaluated its effects on lung inflammation and fibrosis. Differential cell counts in BALF showed a slight but non-significant decrease in the number of macrophages in the bleomycin + INCB3344 group compared with the bleomycin + vehicle group (Fig. 5E). However, the hydroxyproline assay and lung histopathology revealed significantly decreased collagen deposition in the lung tissues of INCB3344-treated mice (Fig. 5F,G). Furthermore, qPCR analysis showed decreased expression of *Col1a1*, *Fn1*, and *Ccl2* in the lungs of these mice (Fig. 5H). Respiratory parameters demonstrated that INCB3344 reduced tissue elastance and tissue damping while increasing inspiratory capacity and static lung compliance (Fig. 5I), suggesting that CCR2 antagonism can ameliorate lung inflammation and fibrosis in bleomycin-treated MDA5 KO mice. These results collectively demonstrate that chemokines involved in CCR2 signaling, released by macrophages, are key mediators in the MDA5-dependent aggravated lung fibrosis induced by bleomycin.

### MDA5 behaves as a scavenger-like receptor to prevent TLR8-mediated chemokine production through lysosomal RNA clearance

**Figure 6:**
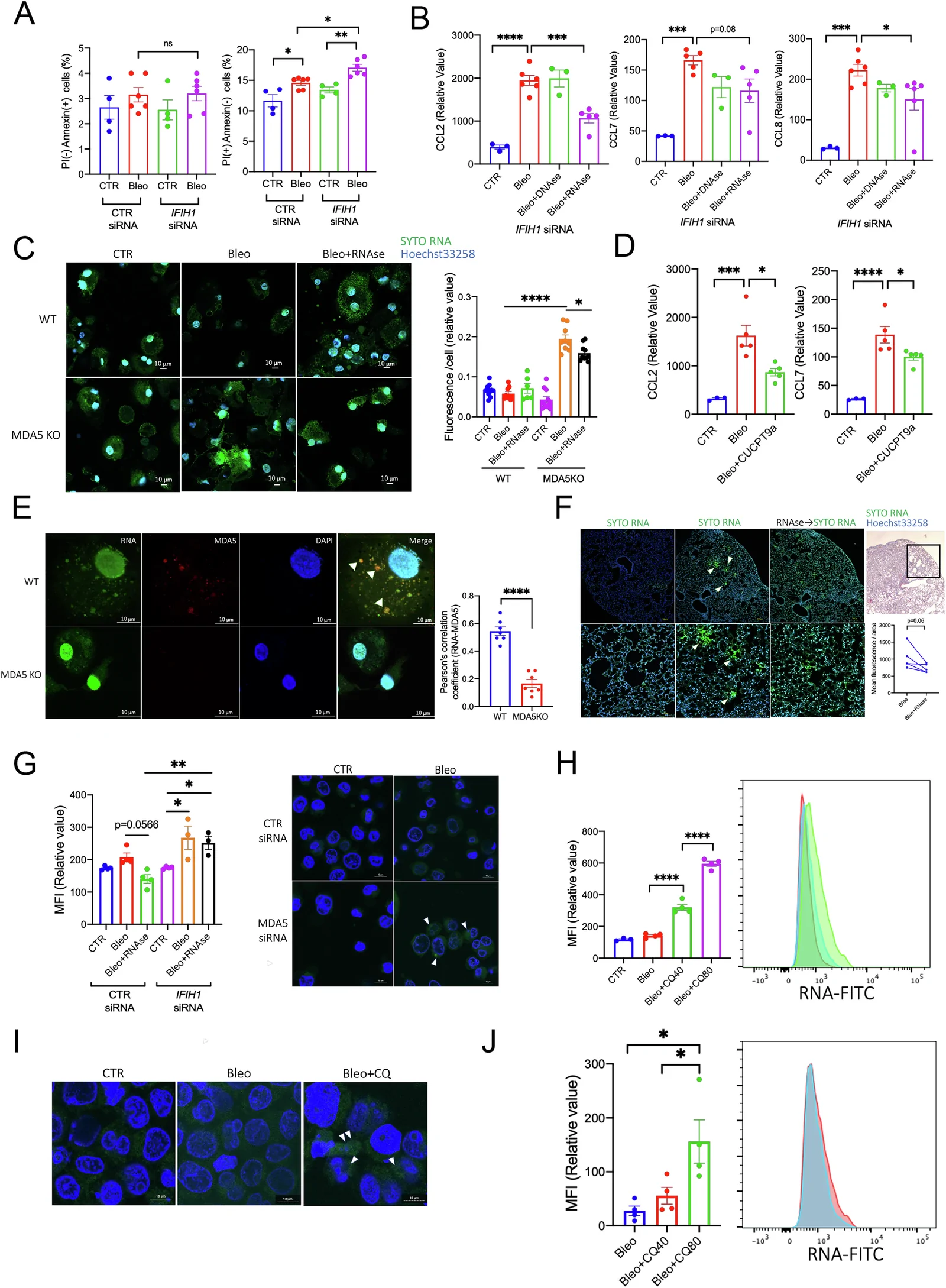
MDA5-mediated lysosomal clearance of extracellular RNA suppresses TLR8-driven inflammatory responses. (**A**) U937 cells with or without *IFIH1* knockdown were treated with control or bleomycin. The proportions of apoptotic cells (Annexin-V ( + ) PI (−)) and necrotic cells (Annexin-V (−) PI (+)) were evaluated (*P* values for necrotic cells: control siRNA vs. *IFIH1* siRNA under bleomycin treatment, *P* = 0.0255; control siRNA CTR vs. control siRNA Bleomycin, *P* = 0.0178; *IFIH1* siRNA CTR vs. *IFIH1* siRNA Bleomycin, *P* = 0.0034). (**B**) *IFIH1*-suppressed U937 cells were treated with control, bleomycin, bleomycin + DNase, or bleomycin + RNase. The expression levels of *CCL2*, *CCL7*, and *CCL8* were measured by qPCR (*P* values for indicated comparisons: Control vs. Bleomycin; *CCL2*, *P* < 0.0001; *CCL7*, *P* = 0.0004; *CCL8*, *P* = 0.0002. Bleomycin vs. Bleomycin + RNase; *CCL2*, *P* = 0.0005; *CCL8*, *P* = 0.0428). (**C**) BMDMs derived from WT or MDA5 KO mice were treated with control, bleomycin, or bleomycin + RNase. Cells were stained with SYTO RNA and Hoechst 33258, and evaluated by confocal fluorescence microscopy (left). Quantification of RNA fluorescence in cytoplasmic + extracellular area is demonstrated (right) (*P* values for WT Bleomycin vs. MDA5 KO Bleomycin, *P* < 0.0001; MDA5 KO Bleomycin vs. MDA5 KO Bleomycin + RNase, *P* = 0.0181). (**D**) *IFIH1*-suppressed U937 cells were treated with control, bleomycin, or bleomycin + CUCPT-9a. The expression levels of *CCL2* and *CCL7* were measured by qPCR (*P* values for Control vs. Bleomycin: *CCL2*, *P* = 0.0007; *CCL7*, *P* < 0.0001. Those for Bleomycin vs. Bleomycin + CUCPT-9a: *CCL2*, *P* = 0.0102; *CCL7*, *P* = 0.0447). (**E**) Super-resolution microscopy of bleomycin-treated WT and MDA5 KO BMDMs without cell permeabilization. Cells were stained for RNA (green), MDA5 (red), and nuclei (DAPI, blue). Arrowheads indicate the spatial colocalization of MDA5 and RNA. Scale bars, 10 µm. Pearson’s Correlation coefficient between RNA and MDA5 in the corresponding cells is indicated (right, *P* < 0.0001). (**F**) In situ staining of RNA (SYTO RNA) in lung sections from bleomycin-treated MDA5 KO mice, with or without prior RNase treatment. Arrows indicate RNA accumulation mainly in the extracellular region. Tissue RNA accumulation was calculated (right lower panel). Original magnification, ×20 (upper panel), ×100 (lower panel). Scale bars, 100 µm. (**G**) U937 cells treated with control or *IFIH1* siRNA were incubated with fluorescently labeled lung tissue-derived RNA (fRNA) or unlabeled control RNA, followed by control or bleomycin treatment for 48 h, with or without RNase treatment immediately prior to cell harvest. Intracellular or cell-surface RNA accumulation was evaluated by measuring the MFI after subtracting the background of the non-labeled control RNA under the same treatment conditions (left), and visualized by confocal microscopy (right). Arrowheads indicate fRNA accumulation. Data are representative of three independent experiments (*P* values for *IFIH1* siRNA CTR vs. *IFIH1* siRNA Bleomycin; *P* = 0.0111; *IFIH1* siRNA CTR vs. *IFIH1* siRNA Bleomycin + RNase; *P* = 0.0418; Control siRNA Bleomycin + RNase vs. *IFIH1* siRNA Bleomycin + RNase; *P* = 0.0022). (**H**–**J**) WT U937 cells were treated with bleomycin and the lysosomal inhibitor chloroquine (CQ; 40 or 80 µM) along with fRNA. (**H**) Accumulation of intracellular and cell-surface RNA was measured by flow cytometry. MFI (left) and a representative histogram (right; red, control; blue, bleomycin; green, bleomycin + CQ 80 µM) are shown. Data are representative of three independent experiments (*P* values for Bleomycin vs. Bleomycin + CQ 40 µM: *P* < 0.0001, Bleomycin + CQ 40 µM vs. Bleomycin + CQ 80 µM: *P* < 0.0001). (**I**) Representative fluorescence microsco*p*y images of the treated cells. Arrowheads indicate fRNA accumulation. (**J**) Evaluation of the augmented reduction in MFI by subsequent RNase treatment under low- or high-dose CQ conditions was evaluated. MFI (left) and a representative histogram (right; blue, bleomycin; red, bleomycin + CQ 80 µM) are shown (*P* values for Bleomycin vs. Bleomycin + CQ 80 µM: *P* = 0.0149, Bleomycin + CQ 40 µM vs. Bleomycin + CQ 80 µM: *P* = 0.0497). Original magnification, ×100. Scale bars, 10 µm for (**C**, **G**, **I**). Data are presented as means ± SEM. *P < 0.05, *P* < 0.01, ***P < 0.001, ns, not significant, by one-way ANOVA with Tukey’s post-hoc test or Welch’s ANOVA followed by Dunnett’s T3 multiple comparisons test (**B**). *n* = 3–5 in each group. Source data are available online for this figure.

MDA5 is an innate immune receptor that specifically recognizes RNA. Since damaged cells release a mixture of both RNA and DNA into the extracellular space, we combined bleomycin treatment with either DNase or RNase to evaluate the potential indirect contribution of extracellular DNA to the inflammatory response. Under these conditions, U937 cells were transfected with control or *IFIH1* siRNA to knock down MDA5 expression, and the levels of inflammatory mediators were determined. We first evaluated cell viability using Annexin-V and propidium iodide (PI) staining. *IFIH1*-suppressed U937 cells showed an increased proportion of PI^+^ cells 24 h after bleomycin treatment and exhibited a progressively larger proportion of PI^+^ cells compared with control siRNA counterparts after 48 h (Fig. EV4C). *IFIH1*-suppressed U937 cells were significantly more susceptible to Annexin-V^−^ / PI^+^ necrotic changes, but not apoptosis, 72 h after bleomycin treatment, compared with control siRNA-treated cells (Fig. 6A). Neither DNase nor RNase influenced bleomycin-triggered necrotic cell death in U937 cells with or without *IFIH1* knockdown (Fig. EV4C, D). However, regarding chemokine production, RNase suppressed *CCL2* and *CCL8* expression only in *IFIH1*-suppressed U937 cells treated with bleomycin (Figs. 6B and EV4E). These results indicate that extracellular RNA, rather than DNA, is the primary trigger for the exaggerated chemokine response.

We then evaluated the distribution of RNA in bleomycin-treated WT and MDA5 KO BMDMs via fluorescence microscopy. Increased RNA fluorescence was detected in MDA5 KO BMDMs compared with their WT counterparts (Fig. 6C). However, this increase was abrogated by RNase treatment, suggesting the existence of extracellular RNA components induced by bleomycin treatment (Fig. 6C). We also measured RNA fluorescence in U937 cells with or without *IFIH1* knockdown. PI^+^ cells showed fluorescence approximately eight-fold higher than PI^-^ cells. Bleomycin-treated *IFIH1*-suppressed U937 cells showed increased RNA fluorescence compared with control siRNA counterparts in both PI-negative and PI-positive cells (Fig. EV4F).

A previous study demonstrated that neutrophil extracellular traps (NETs) released from primary human polymorphonuclear neutrophils (PMNs) trigger a cytokine response that is dependent on NET-associated RNA recognized by TLR8 (Herster et al, 2020). To explore the involvement of TLR8 in the self-RNA-induced chemokine response, we used the TLR8 inhibitor CUCPT9a and found that it suppressed *CCL2* and *CCL7* expression in *IFIH1*-suppressed U937 cells treated with bleomycin (Fig. 6D). Furthermore, similar effects of TLR8 blockade on chemokine production were also observed in experiments using both MDA5 and TLR8 siRNAs (Fig. EV4G). These results suggest that extracellular self-RNA drives chemokine production via the TLR8 pathway, which is aberrantly hyperactivated in the absence of MDA5.

These findings raised the mechanistic question of how MDA5, which lacks intrinsic RNase activity, controls the accumulation of extracellular RNA leading to an innate immune response. We hypothesized that surface-translocated MDA5 acts as a scavenger receptor to actively clear self-RNA. Utilizing super-resolution microscopy on bleomycin-treated WT BMDMs without cell permeabilization, we observed spatial colocalization of MDA5 and RNA signals at the cellular periphery, providing evidence of their interaction (Fig. 6E). We next sought to visualize the distribution of RNA in the fibrotic lung. Consistent with this in vitro finding, in situ staining of lung sections from bleomycin-treated MDA5 KO mice revealed significant RNA accumulation in foci of severe inflammation and fibrosis. The susceptibility of this signal to RNase treatment demonstrated its extracellular localization, suggesting that tissue damage leads to a substantial buildup of extracellular RNA (Figs. 6F and EV4H, left). Similar extracellular RNA accumulation was observed in the severely inflamed and fibrotic lung in WT mice, although in a more limited area compared with MDA5 KO mice (Fig. EV4H, right).

To functionally prove that MDA5 is involved in the process of the cellular uptake and turnover of RNA, we performed a quantitative in vitro clearance assay. We labeled lung tissue-derived RNA with SYTO RNAselect and administered this fluorescent, or non-fluorescent RNA as a control, to siRNA-treated U937 cells. Little fluorescence was detected in the cell culture supernatant 48 h after any of the treatments (Fig. EV4I). Flow cytometric analysis of PI-negative cells revealed that in control siRNA-treated cells, the bleomycin-induced increase in cellular fluorescence was modest, and a portion of the signal was susceptible to RNase digestion, reflecting normal turnover. In stark contrast, *IFIH1* knockdown resulted in a significant and sustained increase in cellular fluorescence (MFI) that was resistant to RNase degradation (Fig. 6G), indicating that in the absence of MDA5, extracellular RNA is not properly processed and degraded, leading to its abnormal accumulation.

Finally, to determine the exact degradation pathway, we treated WT U937 cells with chloroquine (CQ), a lysosomal inhibitor. CQ treatment dose-dependently increased the intracellular accumulation of fluorescent RNA (Fig. 6H,I). Furthermore, under high-dose CQ conditions, the reduction of MFI by subsequent RNase treatment was augmented (Fig. 6J). This indicates that when lysosomal degradation is blocked by CQ, the internalized fluorescent RNA markedly accumulates inside the cells, and consequently, uninternalized RNA stagnates and builds up on the cell surface, becoming highly susceptible to external RNase. Collectively, these comprehensive data demonstrate that MDA5 captures extracellular RNA at the cell surface and shuttles it into the lysosomal degradation pathway; failure of this clearance mechanism results in RNA accumulation and subsequent TLR8-driven chemokine production.

### Essential role of CCR2 ligands and relevance of MDA5 in patients with ILDs or anti-MDA5 autoantibody-positive dermatomyositis

**Figure EV5:**
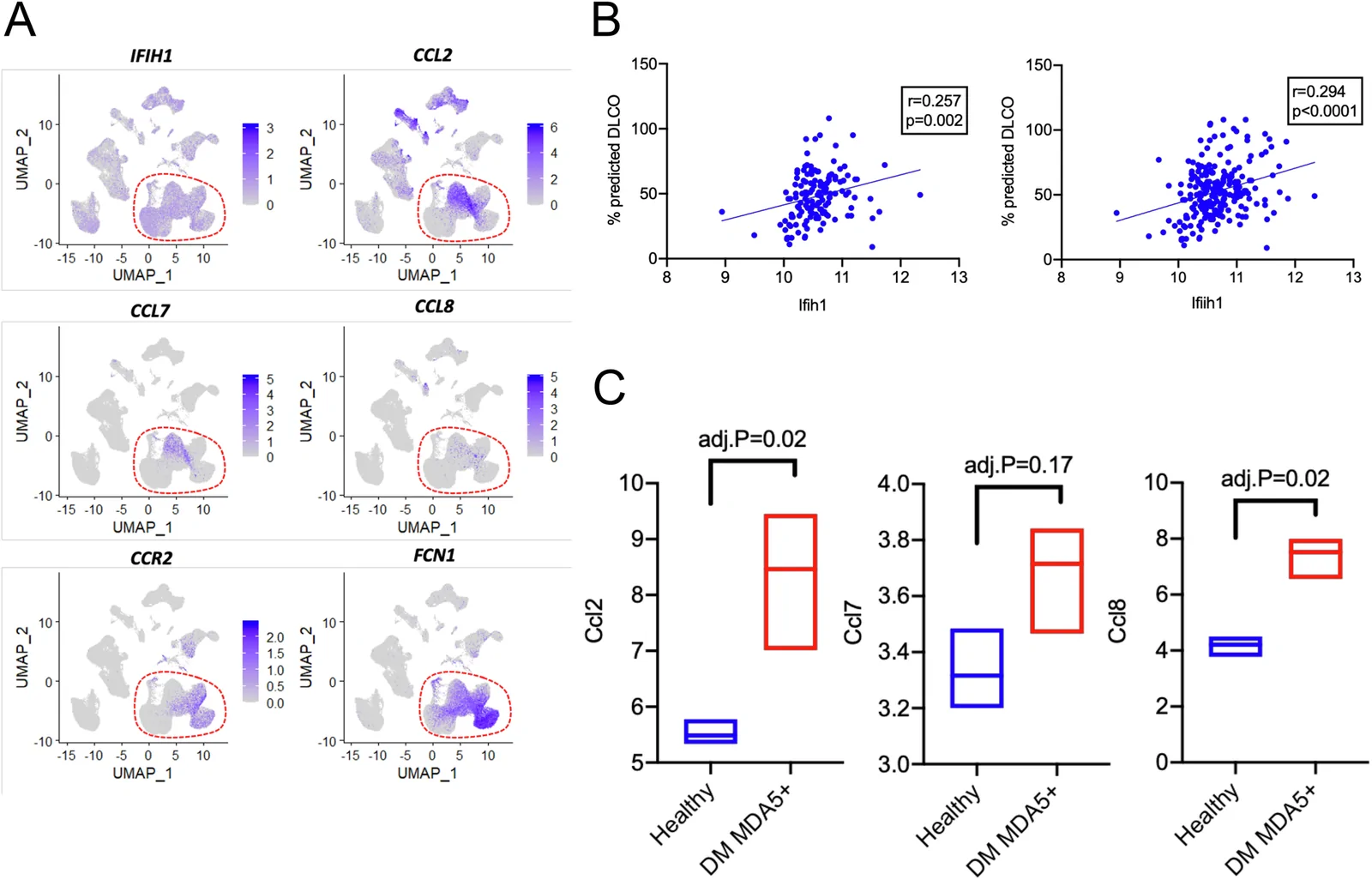
Gene expression of *IFIH1* and related genes with clinical lung function. (**A**) UMAP plots from a single-cell RNA-seq dataset (GSE135893) showing the expression of indicated genes in whole lung cells. The plot showing the expression of *IFIH1* is also shown in Fig. EV1A. The red dashed circle indicates the myeloid cell population. (**B**) Correlation between *IFIH1* expression and percent predicted DLCO (% predicted DLCO) in patients with IPF (left, *n* = 128) and patients with ILD including IPF (right, *n* = 231) from the Lung Genomics Research Consortium (GSE47460) (Spearman correlation: left [IPF], *r* = 0.257, *P* = 0.002; right [ILD], *r* = 0.294, *P* < 0.0001). The Spearman correlation coefficient (*r*) and *P* value are shown. (**C**) Microarray data showing the expression of *CCL2*, *CCL7*, and *CCL8* in skin tissue from healthy controls (*n* = 6) or patients with MDA5 antibody-positive dermatomyositis (DM MDA5 + , *n* = 3) (GSE128314). Box plots show the median and min/max values. Adjusted *P* values (adj.P) are shown. Source data are available online for this figure

**Figure 7:**
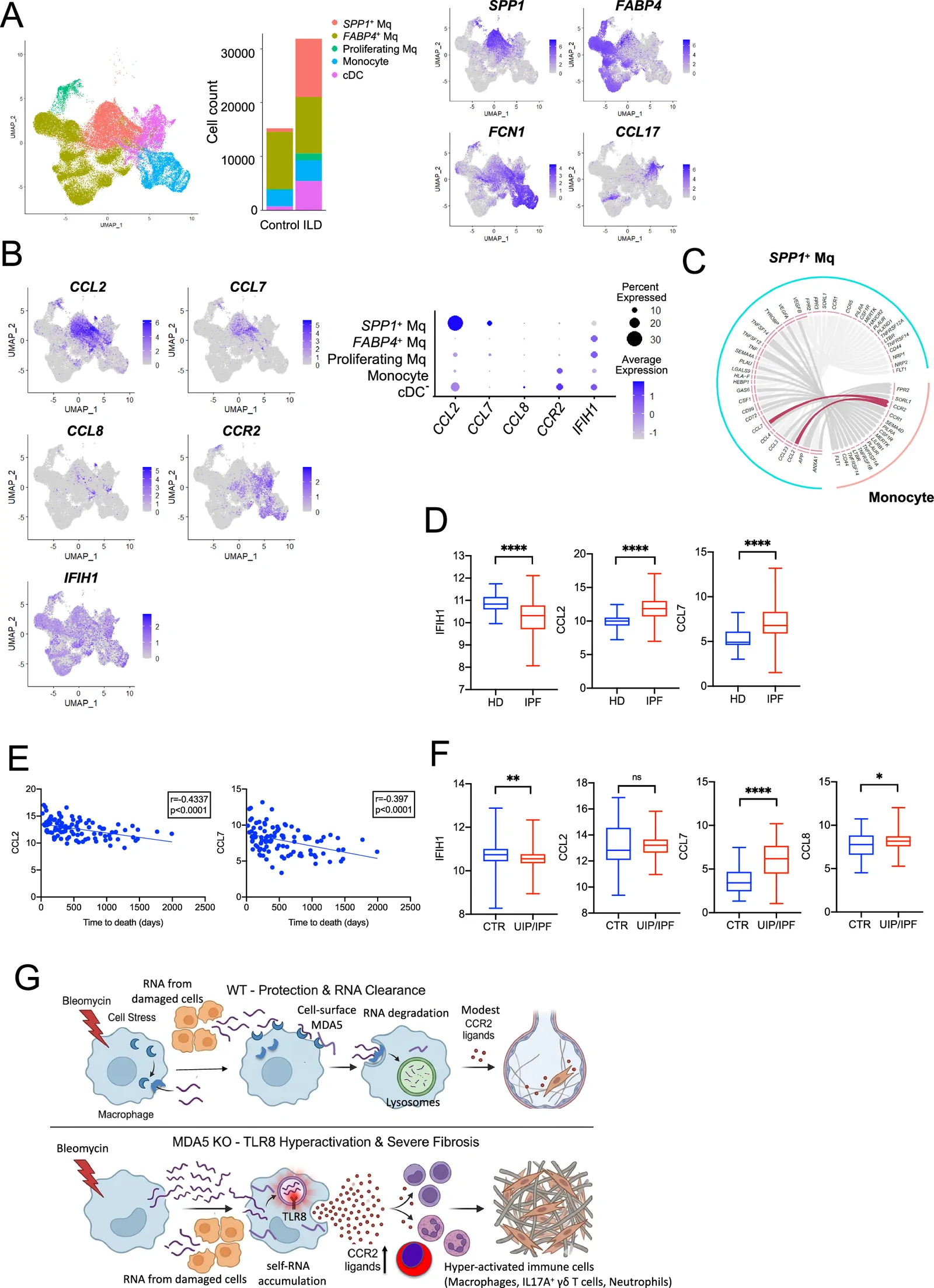
Expression of *IFIH1* and CCR2-related chemokines in patients with ILD and their correlation with clinical characteristics. (**A**–**C**) Single-cell RNA sequencing datasets (GSE135893), consisting of 114,396 cells from 20 ILD patients (Idiopathic Pulmonary Fibrosis (IPF; *n* = 12), chronic hypersensitivity pneumonitis (cHP; *n* = 3), nonspecific interstitial pneumonia (NSIP; *n* = 2), sarcoidosis (*n* = 2), and unclassifiable ILD (*n* = 1)) and 10 control lungs, were subjected to UMAP embedding with new annotations. (**A**) Immune cell clusters representing macrophages, monocytes, and cDCs were re-annotated by specific cell types (*SPP1*^+^ Mq, *FABP*^+^ Mq, proliferating Mq, monocytes, and cDC, left), and the respective cell counts were calculated (middle). Expressions of specific markers (*SPP1, FCN1, FABP4*, and *CCL17*) are illustrated (right). (**B**) Expressions of *CCL2, CCL7, CCL8, CCR2*, and *IFIH1* in the corresponding clusters are presented (left), with proportions and expression levels indicated for each cell type (right). (**C**) Interactions between *SPP1*^+^ macrophages and monocytes were analyzed using CellphoneDB. Only significant interactions (*P* < 0.05) were visualized using a circus plot. (**D**, **E**) Microarray datasets (GSE70866) of the expression analysis in BALF cells from healthy donors or patients with IPF (*n* = 20 and *n* = 175, respectively) were obtained. Expression levels of *CCL2, CCL7*, and *IFIH1* in each group are shown. (**D**) Expression levels of *CCL2*, *CCL7*, and *IFIH1* in BALF cells from healthy donors (HD) and patients with IPF (HD, *n* = 20; IPF, *n* = 175. Each *P* value, *P* < 0.0001. (**E**) Correlation between the expression of *CCL2* (left) or *CCL7* (right) and survival time in patients with IPF (*n* = 100). (**F**) Gene expression profiling of chronic lung disease from the Lung Genomics Research Consortium (GSE47460), using lung homogenates from 108 controls and 160 patients with IPF, was analyzed. The expression of *CCL2, CCL7, CCL8*, and *IFIH1* in each group is presented (*P* values for CTR vs. UIP/IPF: *CCL7*, *p* < 0.0001; *CCL8*, *P* = 0.0135; *IFIH1*, *P* = 0.0013). (**G**) Schematic representation of the proposed mechanism of MDA5-mediated protection against lung fibrosis. (**D**, **F**) Data are presented as box-and-whisker plots. The central line represents the median (50th percentile), and the upper and lower bounds of the box indicate the 75th and 25th percentiles, respectively. The whiskers extend to the minimum and maximum values. *P < 0.05, **P < 0.01, ***P < 0.001. ****P < 0.0001, as determined by Mann–Whitney test. Source data are available online for this figure.

We analyzed the distribution of *IFIH1* in the lungs of patients with ILDs using a public database of single-cell RNA-seq (Data ref: NCBI Gene Expression Omnibus GSE135893, 2019). *IFIH1* was broadly expressed in immune cells, whereas its expression in epithelial cells was relatively low (Fig. EV1A and EV5A). *CCR2*, *CCL2, CCL7*, and *CCL8* were also expressed in various immune cell types (Fig. EV5A). We further analyzed the immune cell populations exhibiting high *IFIH1* expression, identifying specific subsets of macrophages, monocytes, and conventional dendritic cells (cDCs) (Fig. 7A, right panel). In patients with ILDs, the proportions of *SPP1*^+^ macrophages as well as cDCs and proliferating macrophages were increased (Fig. 7A, left panel). Within *SPP1*^+^ macrophages, *CCL2 and CCL7* were broadly and highly expressed compared with other cell types, and a portion of them also expressed *CCL8* (Fig. 7B). *CCR2* was highly expressed in monocytes, and cell-to-cell interaction analysis revealed a strong interaction between *CCL2* and *CCL7* produced by *SPP1*^+^ macrophages and *CCR2* on monocytes (Fig. 7B,C).

Subsequently, we analyzed the expression of *IFIH1* and ligands for CCR2 in BAL cells from patients with IPF using a public microarray dataset (Data ref: NCBI Gene Expression Omnibus GSE70866, 2018). *CCL2* and *CCL7* expression levels were higher, while *IFIH1* expression was lower, in the BAL cells of patients with IPF than in those derived from healthy donors (Fig. 7D). Meanwhile, *CCL2* and *CCL7* expression levels were inversely correlated with patient survival with IPF (Fig. 7E). Another microarray dataset derived from lung homogenates (Data ref: NCBI Gene Expression Omnibus GSE47460, 2013) showed a similar tendency for *CCL7* and *IFIH1* (Fig. 7D), whereas *CCL8* expression, but not *CCL2*, was higher in patients with IPF than in controls (Fig. 7F). Regarding physiological lung function, the percent-predicted diffusing capacity of the lungs for carbon monoxide (%DLCO), which reflects the disease severity of ILDs, slightly correlated with *IFIH1* expression (Fig. EV5B). We also analyzed microarray data of skin tissues from patients with anti-MDA5 autoantibody-positive dermatomyositis. Specifically, *CCL2* and *CCL8* expression levels were significantly elevated in these patients compared with healthy controls (Fig. EV5C). In summary, macrophage-derived CCR2 ligands are crucial for the activation of monocytes in patients with ILDs, and these chemokines, along with MDA5, may be closely associated with the pathogenesis of ILDs. Furthermore, these ligands might also be associated with the pathogenesis of anti-MDA5 autoantibody-positive dermatomyositis.

## Discussion

We have demonstrated that a novel protective role of MDA5 in lung fibrosis is achieved by regulating the clearance of extracellular RNA released during tissue damage. Traditionally regarded almost exclusively as a cytosolic RNA sensor that induces type I interferon signaling for viral defense, MDA5 is shown here to also localize to the cell surface and extracellular region. Notably, bleomycin treatment altered the dynamics and distribution of MDA5. Mechanistically, bleomycin-induced cellular stress triggers the translocation of cytoplasmic MDA5 to the cell surface. Because this surface expression is completely abolished by Brefeldin A (BFA), an inhibitor of ER-to-Golgi transport, the classical ER-to-Golgi secretory pathway is likely involved in this process. Activated MDA5 is known to be redistributed from the cytosol to the mitochondria-associated membrane fraction to trigger the downstream MAVS pathway (Lin et al, 2019). In human neutrophils, RIG-I and MDA5 were detected both in secretory vesicles and on the cell surface as well as in the cytoplasm (Berger et al, 2012). Although their significance has remained largely unknown, their surface expression could be due to plasma membrane reorganization events after fusion with secretory vesicles. Historically, extracellular MDA5 has not been a focal point, partly because only a small amount is detectable in serum according to protein databases. Intriguingly, we found abundant MDA5 in BALF, and its levels decreased following intratracheal bleomycin treatment. MDA5 protein in BALF was likely derived from alveolar macrophages, considering the relatively low MDA5 expression in bronchial and alveolar epithelial cells. An apparent paradox in our findings is the inverse correlation between the decreased extracellular MDA5 levels in the BALF following bleomycin treatment and its concurrently enhanced cell-surface anchoring. We propose that in the steady state, lung macrophages constitutively release a basal level of MDA5 into the extracellular space. However, upon bleomycin-induced tissue injury, cellular dynamics undergo a stress-adaptive shift. Our data indicate that under these conditions, MDA5 actively translocates to and is retained on the plasma membrane via the ER-Golgi pathway to function as an anchored scavenger receptor, which consequently reduces its shedding or free release into the BALF. Furthermore, as our in vivo BALF analysis was conducted at a single time point (day 14 post-bleomycin), this reduction may also reflect the specific time-course kinetics of extracellular protein distribution during the subacute inflammatory phase. Crucially, our direct biochemical isolation of surface proteins confirmed the presence of full-length MDA5 (~140 kDa), ensuring that these observations reflect genuine membrane relocalization rather than the presentation of small MDA5-derived peptides on MHC class I molecules.

Upon bleomycin treatment, MDA5 KO mice exhibited an exacerbated fibrotic phenotype, with elevated proinflammatory mediators in their lungs compared with WT and MAVS KO mice. We demonstrated that bleomycin-treated dKO mice exhibited fibrotic characteristics similar to those of MDA5 KO mice, confirming that the phenotype is independent of the canonical MDA5-MAVS pathway. Furthermore, bleomycin-treated IFNAR KO mice showed fibrotic changes that were partially comparable in severity to MDA5 KO mice. A previous study reported that IFNAR KO mice exhibit exacerbated inflammation and fibrosis due to the loss of the anti-inflammatory roles of type I interferon signaling (Feng et al, 2024). While the overall severity aligned with these previous reports, our detailed analysis revealed distinct underlying mechanisms. Specifically, the expression of *Ccl2* and the interferon-stimulated gene *Mx1*, as well as the proportions of specific macrophage subsets in the lungs, were significantly different between MDA5 KO and IFNAR KO mice. This clearly indicates that the pathogenesis in MDA5 KO mice is not exclusively regulated by the canonical type I IFN-related pathway, but rather driven by distinct signaling pathways and immune profiles. In addition, we observed an increase in the population of IL-17A+ γδ T cells and neutrophils in the lungs of MDA5 KO mice. While other types of γδ T cells producing IFN-γ and IL-22 counteract pulmonary fibrosis (Segawa et al, 2016; Simonian et al, 2010), IL-17A produced by CD4^+^ and γδ T cells induces significant neutrophilia and pulmonary fibrosis when combined with TGF-β (Wilson et al, 2010). The IL-17A-producing γδ T cells-neutrophil axis is associated with pneumonia (Xu et al, 2023) and cancer metastasis (Coffelt et al, 2015), underscoring the pathogenicity of IL-17A-producing γδ T cells. Herein, we observed a significant increase in the number of neutrophils and levels of their relevant proinflammatory markers, namely MIP-2 and CXCL1, which are involved in bleomycin-induced lung fibrosis (Keane et al, 1999), in bleomycin-treated MDA5 KO mice.

We focused on CD11b^+^ macrophages as the primary targets in the MDA5-associated pathogenesis of lung fibrosis, as *Ifih1* was highly expressed in this population, and CD11b^+^ macrophages are known to be involved in the development of bleomycin-induced lung fibrosis (McCub**b**rey et al, 2018; Wan et al, 2023). We observed that in MDA5 KO CD11b^+^ macrophages, the expression of fibrosis-related genes, including those in the collagen family and *Sparc* (Sangaletti et al, 2011), was increased. *Col1a1* expression in pro-fibrotic airway macrophages is a marker of disease progression and poor survival in ILDs (Tsitoura et al, 2021). Furthermore, cytokine and chemokine production and the subsequent activation of signaling pathways were also observed. Similar results regarding chemokines and their signaling pathways were obtained via RNA-seq using WT and MDA5 KO BMDMs. Considering that ligands for CCR2 were common mediators upregulated in both MDA5 KO CD11b^+^ macrophages and BMDMs, we demonstrated that these ligands played an essential role in MDA5-dependent aggravated lung fibrosis. Furthermore, the functional significance of MDA5 within the myeloid compartment was directly demonstrated by our adoptive transfer experiments. The transfer of MDA5 KO BMDMs into WT mice significantly exacerbated bleomycin-induced lung fibrosis compared with the transfer of WT BMDMs. This result provides definitive evidence that the loss of MDA5 specifically in macrophages is a primary driver of the aggravated pathogenesis observed in our model.

CCL2 is an established mediator involved in lung fibrosis through the recruitment of fibrocytes and profibrotic macrophages (Moore et al, 2005; Moore et al, 2001; Suga et al, 1999), while CCL7 is implicated in the pathogenesis of IPF (Choi et al, 2004). Although CCR2 deficiency attenuated lung fibrosis in animal models (Okuma et al, 2004), the blockade of CCL2 failed to demonstrate a clinical advantage in patients with IPF (Raghu et al, 2015). Pulmonary CCR2^+^ CD4^+^ T cells are immunosuppressive and could attenuate lung inflammation and fibrosis, suggesting multifaceted roles for CCR2 (Milger et al, 2017). As shown in Fig. 7, CCR2 blockade could be effective in patients with ILDs in whom *IFIH1* expression is relatively low. In patients with anti-MDA5 autoantibody-associated ILD, overactivated macrophages are key players in the pathogenesis; a previous study demonstrated that *CCL2* expression in circulating monocytes from anti-MDA5-associated ILD was higher than that from anti-aminoacyl-tRNA synthetase (ARS)-associated ILD or healthy controls (Gono et al, 2022), suggesting a similar pathogenesis to our bleomycin-treated MDA5 KO mice. Based on our finding that MDA5 can localize to the cell surface and mediate RNA clearance, one potential hypothesis, although further validation is required, is that these autoantibodies might directly target surface-exposed MDA5. The binding of such antibodies could potentially interfere with the extracellular RNA-scavenging function of MDA5, perhaps via steric hindrance. This dysfunction might lead to the aberrant accumulation of extracellular and intracellular RNA and subsequent activation of TLR8-dependent pro-inflammatory cascades, mirroring the aggravated phenotype observed in our MDA5-deficient models.

Analysis of single-cell RNA-seq data from the lungs of patients with ILDs showed that the characteristics of *SPP1*^+^ macrophages, such as a higher population and increased expression of *CCL2* and *CCL7*, were similar to those of mouse lung CD11b^+^ macrophages. The role of proliferative *SPP1*^*+*^ macrophages in the activation of IPF myofibroblasts during lung fibrosis has been shown (Morse et al, 2019), and *SPP1*^+^ profibrotic monocyte-derived macrophages have been detected across multiple lung diseases, including in COVID-19, pulmonary fibrosis, and lung carcinoma (Sikkema et al, 2023). Our data support the involvement of *IFIH1* in the activation of *SPP1*^+^ macrophages, and that robust production of chemokines, including CCR2 ligands, results in the activation of monocytes and subsequent differentiation into macrophages, leading to the activation of myofibroblasts and fibrosis formation.

Regarding the molecular role of MDA5 in bleomycin-induced aggravated inflammation and fibrosis, cellular RNA released from damaged cells can induce RNA accumulation and increase the amount of CCR2 ligands, revealing a novel role of MDA5 in RNA clearance and the control of these ligands. MDA5 has a high affinity for both single-stranded RNA (ssRNA) and double-stranded RNA (dsRNA), whereas only dsRNA binding triggers filament formation and subsequent ATP hydrolysis (Peisley et al, 2011). Another study showed a low concentration of ssRNA could bridge multiple MDA5 molecules, allowing oligomerization of their CARD domains, while an increased amount of RNA induced RNA redistribution and the bridging effect disappeared, although their combination still existed (Ahmad et al, 2018).

Rather than merely sensing RNA, our in vitro clearance assays utilizing fluorescently labeled RNA and lysosomal inhibition clearly indicate that MDA5 physically shuttles exogenous RNA into the lysosomal degradation pathway. In the presence of MDA5, exogenous RNA is properly captured at the cell surface, and this surface-associated retention renders the RNA highly susceptible to exogenous RNase digestion. Disruption of this mechanism—either via *IFIH1* knockdown or lysosomal blockade with chloroquine—leads to the aberrant influx and intracellular accumulation of RNA, overwhelming the degradation machinery (Fig. 7G). The slight increase in necrotic cells observed around 48 h after bleomycin administration in the MDA5-suppressed group may be partially attributed to this detrimental RNA accumulation. However, it is also important to note the possibility that RNA may be degraded by membrane-bound RNases without intracellular internalization. Additionally, the process by which MDA5 captures and transports cell-surface RNA likely involves other RNA-binding proteins or intracellular transport proteins. A previous study demonstrated that extracellular circular RNA is actively internalized through MSR1-mediated scavenger receptor phagocytosis and macropinocytosis, with TLRs and mTOR signaling serving as key regulators of this uptake (Amaya et al, 2024). While our proposed TLR8 pathway partly relies on extrapolation from previous literature, the exact alternative RNA internalization routes operating under MDA5-deficient conditions remain to be fully defined. Future studies employing specific endocytosis inhibitors, such as Pitstop 2, will be essential to directly test whether MDA5-dependent or alternative endocytic RNA uptake is broadly required for downstream signaling. Identifying these interacting partners and uptake machinery, as well as determining whether other RNA sensors fulfill similar roles, will be critical topics for future research.

Recent evidence has fundamentally expanded our understanding of cell-surface RNAs (csRNAs) and RNA-binding proteins (RBPs) as integral components of the plasma membrane (Perr et al, 2025) (Li et al, 2025). These molecules are organized into highly regulated nanoclusters that govern cellular interactions with the extracellular environment. A primary mechanism for the stable presentation of csRNA involves the formation of a ternary complex consisting of RNA, cell-surface RBPs, and heparan sulfate (Li et al, 2025). Another study demonstrates that RNA-RBP complexes are enriched with glycoRNAs and their structural integrity is functionally dependent on the presence of RNA (Perr et al, 2025). Furthermore, specific RNA species presented on the surface, such as snoRNAs, possess distinct structural traits including frequent UU motifs. These motifs are known agonists for TLR7, suggesting that csRNAs possess the necessary molecular signatures to activate innate immune sensing pathways.

Our results suggest that RNA released from damaged cells upon tissue injury could be bound and cleared by MDA5-associated machinery without triggering downstream signaling. Conversely, MDA5 deficiency leads to pathological RNA accumulation. Similar to how RNA externalized by neutrophil extracellular traps (NETs) promotes inflammatory pathways through endosomal TLRs such as TLR8 (Herster et al, 2020; Blanco et al, 2021), the aberrant accumulation of extracellular and intracellular nucleic acids in the absence of MDA5 activates TLR8 signaling. This ultimately drives the robust production of CCR2-related chemokines, resulting in lung inflammation and fibrosis through the recruitment and activation of multiple immune cell types (Fig. 7G).

In the context of extracellular nucleic acid-driven pathologies, it is worth noting that alongside the extracellular self-RNA focused on in this study, NETs represent another critical source of pathogenic extracellular nucleic acids. Physiologically, NETs are highly vital for host antimicrobial defense via the process of NETosis. However, dysregulated or excessive NETosis can trigger sterile inflammation and contribute centrally to the initiation and perpetuation of systemic autoimmune diseases (Thiam et al, 2020; Wigerblad and Kaplan, 2023). Furthermore, crucial roles of NETosis have been recognized in a wide spectrum of other severe disorders, such as the dysregulated host inflammatory responses in sepsis (Yaykasli et al, 2024), as well as excessive immunothrombosis and acute respiratory distress syndrome during severe viral infections like COVID-19 (Potaczek et al, 2024). Given that MDA5 deficiency significantly exacerbated neutrophil infiltration in our bleomycin-induced lung injury model, the potential interplay between extracellular RNA clearance defects and aberrant NETs-derived DNA and RNA buildup in amplifying pulmonary fibrosis warrants further investigation.

This study has some limitations. Although the role of extracellular RNA in lung fibrosis was independent of the MDA5-MAVS pathway and partially dependent on TLR8, other intracellular MDA5 signaling pathways without MAVS may have been induced. For example, MDA5 inhibits enteric virus replication by inducing a non-canonical IFN-like response that is partially dependent on the JAK-STAT cascade (Li et al, 2020). Mutual regulation between NOD1, which is an innate immune receptor, and MDA5 isoforms may also play a crucial role in the innate immune response (Wu et al, 2020), suggesting the possibility of the influence of other innate immune receptors due to MDA5 deficiency. Although the precise dependence on type I IFN signaling in MDA5 KO-associated ILD pathogenesis remains of high interest, we did not examine MDA5/IFNAR dKO mice in this study. Directly comparing MDA5 KO mice with MDA5/IFNAR dKO mice would be highly informative for definitively dissecting the specific, cell-type-dependent contributions of type I IFN signaling versus alternative innate nucleic acid-sensing pathways in chemokine production and downstream fibrotic signaling. Investigating these crosstalk mechanisms using such double-deficient models remains an important and necessary direction for future work to fully elucidate the multi-layered innate immune networks regulating pulmonary fibrosis. Furthermore, while the re-analysis of publicly available transcriptomic datasets provided valuable human validation, this approach carries inherent limitations. These include a lack of direct control over sample processing, potential batch effects across different clinical cohorts, and the unavailability of complete clinical metadata for all utilized subjects. Finally, although we validated the core molecular mechanisms using a human monocyte cell line and primary mouse macrophages, our study lacks data obtained from primary human lung cells or advanced in vitro*/*ex vivo systems. Future validation utilizing modern models, such as human lung organoids or air-liquid interface cultures, will be essential to further bridge the gap toward clinical translation. Similarly, as we used bleomycin for the induction of lung fibrosis in mice and proinflammatory and profibrotic mediators for in vitro experiments, careful interpretation is required when directly extrapolating in vitro and animal data to human clinical settings, even though our analysis of human ILD datasets remains highly consistent with our model.

In summary, these collective findings allow us to propose a comprehensive model for the novel protective role of MDA5 against lung fibrosis (Fig. 7G). Our work highlights a previously unexpected role of MDA5 in RNA clearance during the pathogenesis of lung fibrosis. Controlling the increased amounts of extracellular RNA upon tissue injury could represent a novel therapeutic strategy for ILD treatment, which may also be applicable to other inflammatory and fibrotic diseases.

## Methods

**Table:** Reagents and tools table

Reagent/resource	Reference or source	Identifier or catalog number
**Experimental models**		
Mouse: C57BL/6N	Sankyo Laboratory	Strain code: C57BL/6N
Mouse: MDA5 KO (C57BL/6N background)	Oriental Bioservice Inc.	N/A (Nature, 2006 May 4;441(7089):101-5)
Mouse: MAVS KO (C57BL/6N background)	RIKEN BioResource Center	Cat# RBRC04546
Mouse: IFNAR1 KO (C57BL/6N background)	Dr. R. Fukui (University of Chiba)	N/A (Gift)
**Cell lines**		
Human: U937 cells	ATCC	Cat# CRL-1593.2
**Antibodies**		
Rabbit polyclonal anti-MDA5 (for IHC)	Proteintech	Cat# 21813-1-AP
Rabbit polyclonal anti-MDA5 (for WB/FCM)	Abcam	Cat# ab79055
Anti-CD16/32 (Fc Block)	BioLegend	Cat# 101302
APC anti-mouse CD11b	BioLegend	Cat# 101212
BV421 anti-mouse CD11c	BioLegend	Cat# 117330
PerCP/Cy5.5 anti-mouse Ly-6C	BioLegend	Cat# 128012
PE/Cy7 anti-mouse MHC Class II	BioLegend	Cat# 107630
PE anti-mouse Ly-6G	BioLegend	Cat# 127608
FITC anti-mouse Siglec-F	BD Biosciences	Cat# 552126
PE/Cy7 anti-mouse CD3	BioLegend	Cat# 100220
PE anti-mouse CD4	BioLegend	Cat# 100408
APC/Cy7 anti-mouse CD8a	BioLegend	Cat# 100714
APC anti-mouse CD19	BioLegend	Cat# 115512
FITC anti-mouse CD45	BioLegend	Cat# 103108
BV421 anti-mouse TCR γ/δ	BioLegend	Cat# 118124
PerCP/Cy5.5 anti-mouse NK-1.1	BioLegend	Cat# 108728
BV510 anti-mouse CD4	BioLegend	Cat# 100533
PE anti-mouse CCR6	BioLegend	Cat# 129804
APC anti-mouse IL-17A	BioLegend	Cat# 506916
Alexa Fluor® 647 Goat anti-Rabbit IgG H&L	Abcam	Cat# ab150079
Goat anti-Rabbit IgG (H + L) Secondary Antibody	Invitrogen	Cat# 65-6120
**Oligonucleotides and other sequence-based reagents**		
Silencing gene expression		
Human IFIH1 siRNA	Sigma-Aldrich/Merck	Cat# SASI_Hs01_00171929
Human TLR8 siRNA	Sigma-Aldrich/Merck	Cat# SASI_Hs01_00064920
MISSION® siRNA Universal Negative Control #1	Sigma-Aldrich/Merck	Cat# SIC001
RT-qPCR primerrs		
hCCL7_QF (Human CCL7 forward qPCR primer)	IDT	Sequence: 5’-GAGGGGCTGAGACCAAACC-3'
hCCL7_QR (Human CCL7 reverse qPCR primer)	IDT	Sequence: 5’-TGAAGTATTAATCCCAACTGGCT-3'
mCCL7_QF (Mouse CCL7 forward qPCR primer)	IDT	Sequence: 5’-CCCTGGGAAGCTGTTATCTTCAA-3'
mCCL7_QR (Mouse CCL7 reverse qPCR primer)	IDT	Sequence: 5’-CTCGACCCACTTCTGATGGG-3'
hTLR8_QF (Human TLR8 forward qPCR primer)	IDT	Sequence: 5’-TTCTGCGCTGCTGCAAGTTA -3'
hTLR8_QR (Human TLR8 reverse qPCR primer)	IDT	Sequence: 5’-GGGTCTTGCTGATGTTCCCT -3'
mIFNa_QF (Mouse IFNa forward qPCR primer)	IDT	Sequence: 5’-TCTGATGCAGCAGGTGGG -3'
mIFNa_QR (Mouse IFNa reverse qPCR primer)	IDT	Sequence: 5’- AGGGCTCTCCAGACTTCTGCTCTG-3'
mMx1_QF (Mouse Mx1 forward qPCR primer)	IDT	Sequence: 5’- GATCCGACTTCACTTCCAGATGG-3'
mMx1_QR (Mouse Mx1 reverse qPCR primer)	IDT	Sequence: 5’- CATCTCAGTGGTAGTCAACCC-3'
mIL17A_QF (Mouse IL17A forward qPCR primer)	IDT	Sequence: 5’-CGTCACCCTGGACTCTCCA -3'
mIL17A_QR (Mouse IL17A reverse qPCR primer)	IDT	Sequence: 5’- CAGAATTCATGTGGTGGTCCA-3'
hCCL2_Fwd (Human CCL2 forward qPCR primer)	IDT	Sequence: 5’-AGTCTCTGCCGCCCTTCT-3'
hCCL2_Rev (Human CCL2 reverse qPCR primer)	IDT	Sequence: 5’-GTGACTGGGGCATTGATTG-3'
hIFIH1_Fwd (Human IFIH1 forward qPCR primer)	IDT	Sequence: 5’-AGGCACCATGGGAAGTGAT-3'
hIFIH1_Rev (Human IFIH1 reverse qPCR primer)	IDT	Sequence: 5’- ATTTGGTAAGGCCTGAGCTG-3'
mFN1_Fwd (Mouse FN1 forward qPCR primer)	IDT	Sequence: 5’-CGGAGAGAGTGCCCCTACTA-3'
mFN1_Rev (Mouse FN1 reverse qPCR primer)	IDT	Sequence: 5’-CGATATTGGTGAATCGCAGA-3'
mCOL1A1_Fwd (Mouse Col1a1 forward qPCR primer)	IDT	Sequence: 5’-CATGTTCAGCTTTGTGGACCT-3'
mCOL1A1_Rev (Mouse Col1a1 reverse qPCR primer)	IDT	Sequence: 5’-GCAGCTGACTTCAGGGATGT-3'
mTGFb1_Fwd (Mouse TGFb1 forward qPCR primer)	IDT	Sequence: 5’- GGAAAGGGCCCAGCACCTGCA-3'
mTGFb1_Rev (Mouse TGFb1 reverse qPCR primer)	IDT	Sequence: 5’- GGTCCTTCCTAAAGTCAATGTA-3'
mCCL2_Fwd (Mouse/Human Ccl2 forward primer)	IDT	Sequence: 5’- ATCCCAATGAGTAGGCTGGAGA-3'
mCCL2_Rev (Mouse/Human Ccl2 reverse primer)	IDT	Sequence: 5’- TAATGTATGTCTGGACCCATTCC-3'
MIP2_Fwd (Mouse Cxcl2 forward primer)	IDT	Sequence: 5’- AAAATCATCCAAAAGATACTGAACAA-3'
MIP2_Rev (Mouse Cxcl2 reverse primer)	IDT	Sequence: 5’-CTTTGGTTCTTCCGTTGAGG -3'
CXCL1_Fwd (Mouse Cxcl1 forward primer)	IDT	Sequence: 5’- GACTCCAGCCACACTCCAAC-3'
CXCL1_Rev (Mouse Cxcl1 reverse primer)	IDT	Sequence: 5’-TGACAGCGCAGCTCATTG-3'
Genotyping Primers		
MAVS genotyping reverse primer	Depositor’s primer	Sequence: 5’-AGGTAGCTCTGATAAACTCG-3'
MAVS genotyping forward primer	Depositor’s primer	Sequence: 5’-TCTGGAGATCCTGCCTTACC-3'
MAVS genotyping neo-cassette primer	Depositor’s primer	Sequence: 5’-TGGATTGCACGCAGGTTCTC -3'
MDA5 targeted-gene-specific primer	Depositor’s primer	Sequence: 5’- CTCTTCTAAGCGTTCCCTGGCTAGTGT-3'
MDA5 upstream-specific primer	Depositor’s primer	Sequence: 5’-CTTGGGAAACAGCTCAGTAAAACTGCC-3'
MDA5 neo-resistance gene-specific primer	Depositor’s primer	Sequence: 5’- CTAAAGCGCATGCTCCAGACTGCCTTG-3'
**Chemicals, enzymes, and other reagents**		
Bleomycin sulfate	Toronto Research Chemicals	Cat# B595800
INCB3344	Selleck Biotech	Cat# S7310
CUCPT9a	Invivogen	Cat# inh-cc9a
Chloroquine (CQ)	FUJIFILM Wako Pure Chemical Corporation	Cat# 038-17971
Brefeldin A (BFA)	BioLegend	Cat# 420601
Recombinant Mouse M-CSF	FUJIFILM Wako Pure Chemical Corporation	Cat# 135-14391
Recombinant Mouse PDGF-BB	BioLegend	Cat# 558802
Ribonuclease A from bovine pancreas (RNase A)	Sigma-Aldrich	Cat# R6513
Deoxyribonuclease I	Sigma-Aldrich	Cat# AMPD1 (or 10104159001)
SYTO™ RNASelect™ Green Fluorescent Cell Stain	Invitrogen/Thermo Fisher Scientific	Cat# S32703
Hoechst 33258	Thermo Fisher Scientific	Cat# H3569
Citrate buffer (pH 6)	Merck Millipore	Cat# 101309
Fluoro-KEEPER Antifade Reagent (Non-Hardening)	Nacalai Tesque	Cat# 12593-64
4x Laemmli Sample Buffer	Bio-Rad	Cat# 1610747
Extra PAGE One Precast Gel	Nacalai Tesque	Cat# 11115-44 (series)
Blocking One	Nacalai Tesque	Cat# 03953-95
Chemi-Lumi One Ultra	Nacalai Tesque	Cat# 11644-40
Hydroxyproline Colorimetric Assay Kit	BioVision (now Abcam)	Cat# K555 (ab222941)
Mouse IFIH1/MDA5 ELISA Kit	LSBio	Cat# LS-F19794
Mouse TNF-alpha ELISA MAX Standard	BioLegend	Cat# 430901
NucleoSpin® RNA Plus	Macherey-Nagel/Takara Bio	Cat# 740984.50
GenomONE®-Si siRNA Transfection Kit	Ishihara Sangyo/Cosmo Bio	Cat# ISK-GS-001-EX
FITC Annexin V Apoptosis Detection Kit with PI	BioLegend	Cat# 640914
Histofine® Simple Stain MAX PO (R)	Nichirei Biosciences	Cat# 424131
Peroxidase Stain DAB Kit	Nacalai Tesque	Cat# 07312-34
Pierce™ Cell Surface Biotinylation and Isolation Kit	Thermo Fisher Scientific	Cat# A44390
Pierce™ 660 nm Protein Assay Reagent	Thermo Fisher Scientific	Cat# 22660
Phosphate-Buffered Saline (PBS)	Nacalai Tesque	Cat# 14249-24
DMEM/Ham’s F-12 Medium	Nacalai Tesque	Cat# 05177-15
RPMI 1640 Medium	Nacalai Tesque	Cat# 30264-56
Hanks’ Balanced Salt Solution (HBSS)	Nacalai Tesque	Cat# 17461-05
Normal Saline “Otsuka”	Otsuka Pharmaceutical Factory	N/A (for clinical use)
Fetal Bovine Serum (FBS)	Biowest	Cat# S1810
RBC Lysis Buffer	BioLegend	Cat# 420301
DNase I (from bovine pancreas)	Sigma-Aldrich	Cat# DN25
Propidium Iodide (PI)	Sigma-Aldrich	Cat# P4170
Collagenase	FUJIFILM Wako Pure Chemical	Cat# 037-17603
Recombinant RNase Inhibitor	Takara Bio	Cat# 2311 A
PrimeScript Reverse Transcriptase	Takara Bio	Cat# 2680 A
Amicon Ultra-2 Centrifugal Filter Unit (3 kDa MWCO)	Merck/Sigma-Aldrich	Cat# UFC200324
Glass Bottom Dish (No. 1S Collagen-coated)	Matsunami Glass	Cat# D11134H
Oligo dT Primer	Takara Bio	Cat# 3805
dNTP Mixture (10 mM)	Toyobo	Cat# NTP-301
TURBO DNase	Thermo Fisher Scientific/Ambion	Cat# AM2238
**Software**		
FlowJo software (v10 or relevant)	BD Biosciences	RRID:SCR_008520
CLC Genomics Workbench	QIAGEN	RRID:SCR_011853
ComplexHeatmap	Bioconductor	RRID:SCR_017270
Enrichr	Web tool	RRID:SCR_001575
DAVID	Web tool	RRID:SCR_018803
Seurat	R package	RRID:SCR_007322
CellPhoneDB	Python/Web tool	RRID:SCR_017120
GraphPad Prism 8	GraphPad Software	RRID:SCR_002798
**Other**		

### Animals

Male C57BL/6N mice (8–12 weeks old) were purchased from Sankyo Laboratory, and age-matched MDA5 KO male mice were purchased from Oriental Bioservice Inc. MAVS KO mice (Oshiumi et al, 2011) were purchased from RIKEN BioResource Center with the approval of Dr. T. Seya, who originally generated this strain. Interferon alpha receptor 1 (IFNAR) KO mice (Fukui et al, 2018) were kindly provided by Dr. R. Fukui (The University of Chiba, Chiba, Japan). All genetically engineered mice were based on a C57BL/6N background. All mice were housed in a specific-pathogen-free environment with free access to food and water. All animal care and experimental procedures were conducted in strict compliance with the ethical guidelines for animal experiments under the approval of the Institutional Animal Experiments Committee of Musashino University (Approval reference number: 05-A-2024), and the care and use of the animals followed the Principles of Laboratory Animal Care. A randomization procedure was performed for allocating animals to treatment.

### Murine ILD model with bleomycin treatment

Bleomycin sulfate was obtained from Toronto Research Chemicals. Mice were intratracheally administered bleomycin (0.33 mg/kg, unless specified) in 50 µL normal saline on day 0 under anesthesia with ketamine (90 mg/kg) and medetomidine (1 mg/kg) administered via intraperitoneal injection. Mice treated with normal saline were used as controls. On day 21, mice were euthanized under anesthesia after undergoing chest CT scanning (LaTheta LCT-200) was performed. BALF was collected to determine cell counts (Ishii et al, 2017). RNA extraction from the lungs was performed using Trizol reagent. cDNA synthesis and qPCR were performed as described previously (Ishii et al, 2018). Lung hydroxyproline assay was performed using the appropriate kit (Biovision). In some experiments, mice were euthanized on day 14, and their BALF was used to measure MDA5 levels by ELISA (LSBio).

### Assessment of the effect of INCB3344 in bleomycin-induced lung fibrosis in MDA5 KO mice

A CCR2 antagonist INCB3344 (Selleck Biotech) dissolved in saline containing 10% dimethylsulfoxide (DMSO) was administered intraperitoneally with a dosage of 5 mg/kg body weight/day. The control group received an equivalent amount of 10% DMSO only. MDA5 KO mice treated with bleomycin on day 0 were treated with INCB3344 or vehicles on days 0, 3, 7, 10, 14, and 17. On day 21, physiological lung function was assessed before BALF, and lung tissues were harvested. Differential cell counts of BALF, as well as hisopathological analysis, qPCR analysis, and hydroxyproline assay using harvested lung tissues, were performed.

### Assessment of physiological lung function

Physiological lung function was assessed on day 21 using a Flexivent system (SCIREQ). Mice underwent tracheostomy while under anesthesia with ketamine and medetomidine. The animals were ventilated at a tidal volume of 8 mL/kg and a respiratory frequency of 150/min with a positive end-expiratory pressure of 2 cm H_2_O. Rocuronium bromide was used for muscle relaxation. Airway resistance (Rrs), inspiration capacity (IC), tissue damping (G), tissue elastance (H), and lung static compliance (Cst) were measured by using the Snapshot-150, the Quick-prime 3, and the pressure-volume loops perturbation maneuver.

### Lung histopathology and immunohistochemistry staining

For histopathology, mice were anesthetized, and a tracheal cannula was inserted. The lungs were then inflated with 4% paraformaldehyde (for fixation), embedded in paraffin, and cut into 5-µm sections. To assess the histopathological characteristics of lung fibrosis, Masson-trichrome was used to stain the lung sections. For immunohistochemistry staining, lung sections were immersed in a citrate buffer (pH 6; Merk Millipore) after deparaffinization and rehydration. After blocking with 3% H_2_O_2_ in methanol, the slides were incubated overnight with the anti-MDA5 antibody (1:400; Proteintech) at 4 °C. Immunoreactivity was detected using Histofine® Simple Stain Max Pro (R). Finally, the slides were stained using the peroxidase stain DAB kit (Nacalai Tesque) and counterstained in Mayer’s hematoxylin solution.

### Flow cytometric analysis and cell sorting

Preparation of single-cell suspensions using enzyme-digested lung tissue from treated mice and staining with antibodies for flow cytometric analysis (Ishii et al, 2017), as well as intracellular staining, is described (Murakami et al, 2020). The following antibodies were used for analysis of monocytes, dendritic cells, and macrophages: APC-conjugated anti-CD11b (1:400), BV421-conjugated anti-CD11c (1:250), PerCP-Cy5.5-conjugated anti-Ly6c (1:400), PE-Cy7-conjugated anti-MHC II (1:400), PE-conjugated anti-Ly6G (1:400), and FITC-conjugated anti-siglec-F (1:400). For analysis of lymphocytes, the following antibodies were used: PE-Cy7-conjugated anti-CD3 (1:400), PE-conjugated anti-CD4 (1:400), APC-Cy7-conjugated anti-CD8a (1:400), APC-conjugated anti-CD19 (1:400), FITC-conjugated anti-CD45 (1:400), BV421-conjugated anti-γδTCR (1:250), and PerCP-Cy5.5-conjugated anti-NK1.1 (1:400). For intracellular IL-17A staining, the following antibodies were used: PE-Cy7-conjugated anti-CD3 (1:400), BV510-conjugated anti-CD4 (1:400), FITC-conjugated anti-CD45 (1:400), PE-conjugated anti-CCR6 (1:400), BV421-conjugated anti-γδTCR (1:250), and APC-conjugated anti-IL-17A (1:1000). Anti-CD16/32 antibody (1:400) was used for blocking of Fc receptors. For analysis of MDA5 expression, anti-MDA5 antibody (Abcam, 1:500) and Alexa Fluor® 647-conjugated anti-Rabbit IgG H&L (1:1000), as the secondary antibody, were used. During evaluation, cells stained with only the secondary antibody were also used. Prepared cells were subjected to flow cytometric analysis using FACSLyric (BD Biosciences). Data were analyzed using FlowJo software. A cell sorter (Cell Sorter SH800) was used to isolate CD11c^int^ Ly6G^high^ lung neutrophils and CD11b^+^ lung macrophages with characteristics of CD11c^+^ CD11b^+^ siglecF^+^ Ly6c^int-+^. Their RNA was extracted using Nucleospin® RNA plus kit for subsequent qPCR or RNA-seq analysis.

### Culture and siRNA treatment of U937 cells

The monocyte leukemia cell line U937 which were tested for mycoplasma contamination at the initial assessment (American Type Culture Collection) was cultured in RPMI medium containing 10% FBS and antibiotic–antimycotic mixed stock solution. Plated cells were incubated with the MDA5 gene *IFIH1* siRNA (SASI_Hs01_00171929) *TLR8* siRNA (SASI_Hs01_00064920), or control siRNA using the GenomONE®-Si siRNA transfection kit for 24 h before treatment with 0.33 μg/mL of bleomycin or bleomycin with 5 μg/mL of deoxyribonuclease I RNase-free solution or 20 μg/mL of ribonuclease A solution from bovine pancreas (Sigma-Aldrich). To evaluate TLR8 involvement, 10 μM of CUCPT9a (Invivogen) was used together with bleomycin. Cells were harvested to evaluate apoptotic or necrotic cell death (FITC Annexin V Apoptosis Detection Kit with PI, Biolegend) using qPCR analysis 72 h after the treatments. Their RNA was extracted using the Nucleospin® RNA plus kit.

### Preparation of fluorescent RNA and in vitro clearance assay

To visualize and quantify extracellular RNA uptake and degradation, total RNA was first extracted from wild-type (WT) mouse lungs using TRIzol reagent (Invitrogen). The isolated lung RNA was incubated with SYTO™ RNASelect™ Green Fluorescent Cell Stain (Invitrogen, 1:5000 dilution) for 30 min to generate fluorescent RNA (fRNA). Unbound dye was removed via standard ethanol precipitation, and the fRNA pellet was dissolved in RNase-free water. Control RNA was prepared in parallel by treating lung RNA with phosphate-buffered saline (PBS) instead of the SYTO dye. Both fRNA and control RNA were adjusted to a concentration of 1000 ng/μL. The fluorescence intensity of the fRNA was validated by adding it to the culture medium and measuring the fluorescence in the FITC channel using a Tristar LB941 multimode microplate reader (Berthold Technologies).

For the in vitro clearance assay, U937 cells were transfected with control or *IFIH1* siRNA as described above. The cells were then seeded into 96-well plates at a density of 1 × 10^5^ cells/well. The cells were treated with either PBS (control group) or 6 μg/mL bleomycin, and simultaneously administered 2 μg of either control RNA or fRNA per well. After 48 h of incubation, the culture supernatants were collected to measure residual extracellular fluorescence using the multimode plate reader.

The cells were subsequently harvested, washed with PBS, and stained with propidium iodide (PI) to distinguish live and dead cells. To evaluate the specific intracellular degradation of RNA, a subset of the bleomycin-treated cells was subjected to RNase digestion by incubating with RNase A (40 μg/mL, R6513, Sigma-Aldrich) at 37 °C for 30 min, followed by washing. The intracellular accumulation of fRNA was then quantified by measuring the mean fluorescence intensity (MFI) in the FITC channel specifically within the PI-negative live cell population using a BD FACSLyric flow cytometer (BD Biosciences). To account for background fluorescence, the final MFI for each fRNA-treated group was calculated by subtracting the MFI of the corresponding control RNA-treated group. Data were analyzed using FlowJo software (BD Biosciences).

### Evaluation of fRNA dynamics using chloroquine

To investigate the role of the lysosomal pathway in extracellular RNA clearance, an in vitro fRNA assay was performed using WT U937 cells. The experimental procedure was largely identical to the fRNA clearance assay described above. Briefly, WT U937 cells (1×105 cells/well) were administered 2 μg/well of fRNA along with 6 μg/mL bleomycin, in the presence or absence of chloroquine (CQ; 40 or 80 μM, 038-17971, FUJIFILM Wako Pure Chemical Corporation). After 48 h of incubation, the cells were harvested, and the intracellular fluorescence intensity was measured using flow cytometry as described above.

For the morphological evaluation of RNA accumulation, a subset of the harvested cells was centrifuged onto glass slides using a cytocentrifuge at 250 rpm for 2 min. The cells were then fixed with methanol and counterstained with Hoechst for nuclear visualization. The intracellular accumulation and localization of fRNA were observed and imaged using a Nikon AX confocal laser scanning microscope (Nikon).

### Evaluation of MDA5 intracellular transport using Brefeldin A

To investigate whether the cell surface translocation of MDA5 depends on the classical ER-to-Golgi secretory pathway, Brefeldin A (BFA) treatment was performed. Wild-type (WT) U937 cells were first stimulated with 6 μg/mL of bleomycin for 24 h. Subsequently, the cells were treated with BFA (420601, BioLegend) at a final concentration of 5 μg/mL (1:1000 dilution) or PBS as a vehicle control. After an additional 12 h of incubation, the cells were harvested. The intracellular and surface distribution of MDA5 and RNA were analyzed by flow cytometry and confocal microscopy. For morphological evaluation, cytospin preparations were generated by centrifuging the cells onto glass slides at 250 rpm for 2 min, followed by methanol fixation and staining as described above.

### Isolation and incubation of murine BMDMs and alveolar macrophages

Bone marrow cells derived from each mouse were collected and incubated with DMEM/F-12 containing 10% FBS, antibiotic–antimycotic mixed stock solution, and 2 mg/mL mouse recombinant M-CSF (Wako-Fujifilm) (Toda et al, 2021). Over 90% of the cells were then confirmed to be positive for CD11b by flow cytometry. On day 7, cells were treated with 1 μg/mL of bleomycin. On day 9, the cells were harvested for qPCR and RNA-seq analysis or subjected to RNA staining. To evaluate the cellular response to growth factor stimulation, BMDMs derived from WT, MDA5 KO, and dKO mice were subjected to serum starvation for 24 h. Subsequently, the cells were stimulated with 40 ng/mL of recombinant mouse PDGF-BB (558802, BioLegend) for 6 h. After the treatment, total RNA was extracted from the cells and used for qPCR analysis. The isolation and culture of alveolar macrophages were performed as previously described (Ishii et al, 2017). Seventy-two hours after PBS or bleomycin (6 μg/mL) treatment, the culture supernatants were harvested to measure TNF-α levels using an ELISA kit (430901, BioLegend).

### Adoptive transfer of BMDMs

For the BMDM transplantation experiments, BMDMs derived from WT or MDA5 KO mice were cultured in 10-cm dishes and stimulated with 6 μg/mL bleomycin on day 7. On day 10, the cells were harvested, washed with PBS, and the cell number was determined. Recipient WT male mice (10–12 weeks old) were anesthetized with a combination of ketamine and medetomidine. Each recipient mouse received an intratracheal administration of 4 × 10^5^ BMDMs (either WT or MDA5 KO) co-administered with 2 μg of bleomycin in a total volume of 60 μL PBS. Two weeks after the administration, the mice were sacrificed, and lung tissues and BALF were harvested for CT imaging, histological analysis, and biochemical assays to compare the effects of WT and MDA5 KO BMDM transplantation.

### Fluorescence imaging and RNA staining

For super-resolution analysis of MDA5 and RNA colocalization, cultured BMDMs or U937 cells were washed with PBS and blocked with 5% BSA in PBS for 1 h at room temperature. To specifically label cell-surface MDA5, the cells were incubated with an anti-MDA5 primary antibody (1:250, ab79055, Abcam) for 1 h at room temperature prior to fixation. Subsequently, the cells were fixed with methanol. This specific sequence and fixative were chosen because conventional paraformaldehyde (PFA) fixation interferes with the RNA Select dye. Following fixation, the cells were simultaneously incubated with an Alexa Fluor 647-conjugated anti-rabbit IgG secondary antibody (Abcam), SYTO™ RNA Select™ green fluorescent cell stain (1:5000, Thermo Fisher Scientific), and Hoechst 33258 for nuclear counterstaining. Super-resolution images were acquired using a Nikon AX confocal microscope equipped with an NSPARC (Nikon Spatial Array Confocal) detector (Nikon), which enables structural observation beyond the conventional diffraction limit.

For the in situ assessment of RNA accumulation in vivo, lung tissues were fixed with methanol, embedded in paraffin, and cut into 5-µm sections. After deparaffinization, the tissue sections were stained with SYTO™ RNASelect™ and observed under a confocal fluorescent microscope (Nikon-AX/AXR, Nikon).

In addition, for standard confocal analysis of total MDA5 expression, BMDMs were fixed with 4% PFA, incubated with the anti-MDA5 primary antibody (1:1000), and subsequently stained with the Alexa Fluor 647-conjugated secondary antibody prior to observation. To determine RNA fluorescence intensity in the cytoplasmic and extracellular regions per cell, FITC signal intensity per unit area was measured using the Nikon AX/AXR analysis software. Nuclear regions were excluded based on fluorescence intensity thresholding. The measured fluorescence intensity was then divided by the total number of cells within the corresponding unit area to calculate the average intensity per single cell in the non-nuclear region. To evaluate the colocalization of RNA and MDA5, Pearson’s correlation coefficient between FITC and Alexa Fluor 647 fluorescence signals was calculated on a single-cell basis using the software.

### RNA staining in lung tissues

For in situ RNA detection, 5-μm-thick paraffin-embedded sections of methanol-fixed lung tissues were used. Following deparaffinization, the sections were incubated with SYTO™ RNASelect™ Green Fluorescent Cell Stain (1:5000) and Hoechst 33258 for 45 min at room temperature. To confirm the specificity of the RNA staining, a subset of sections was treated with RNase A (40 μg/mL, Sigma-Aldrich) at 37 °C for 30 min prior to the SYTO staining. After washing with PBS, the slides were mounted using Fluoro-KEEPER Antifade Reagent (Non-Hardening Type; Nacalai Tesque) and observed under a Nikon AX confocal microscope (Nikon). For RNA fluorescence quantification in lung tissue sections, FITC fluorescence intensity per unit area was measured. The obtained values were spatially matched and paired between RNase-treated and PBS-treated) conditions at identical microscopic locations.

### Isolation of cell surface proteins

To evaluate the surface translocation of MDA5, cell surface proteins were isolated using the Pierce™ Cell Surface Biotinylation and Isolation Kit (A44390, Thermo Fisher Scientific) according to the manufacturer’s instructions. Briefly, U937 cells were transfected with control or *IFIH1* siRNA. The following day, the cells were treated with 6 μg/mL bleomycin and incubated for 48 h. The cells were then harvested and washed with PBS. Cell viability was confirmed to be 85–90% using trypan blue exclusion to ensure that intracellular proteins were not aberrantly biotinylated due to compromised membrane integrity. For each condition, 1 × 10^7^ intact cells were surface-biotinylated, lysed, and the labeled surface proteins were isolated using standard avidin-agarose affinity chromatography. The eluted cell surface protein fractions were subsequently concentrated using Amicon® Ultra-2 mL centrifugal filters with a 3 kDa molecular weight cutoff (Merck Millipore). The protein concentrations of the concentrated samples were determined using the Pierce™ 660 nm Protein Assay Reagent, as described in the Western blotting section, and the samples were subsequently subjected to Western blot analysis.

### Western blotting

Protein concentrations of the samples were determined using the Pierce™ 660 nm Protein Assay Reagent (Thermo Fisher Scientific) to ensure equal loading. For cell surface (CS) protein fractions, samples were prepared from an equal number of initial cells (1 × 10^7^ cells), and their protein concentrations were subsequently equalized. Protein samples (5 μg for CS protein fractions and 20 μg for bronchoalveolar lavage fluid (BALF) proteins) were mixed with 4× Laemmli Sample Buffer (Bio-Rad, #1610747) containing a reducing agent, and denatured by heating at 75 °C for 10 min. The samples were then separated by SDS-PAGE using Extra PAGE One Precast Gels (13-well, Nacalai Tesque) at 110 V for 90 min. The separated proteins were transferred onto PVDF membranes using a semi-dry transfer system (WSE-4025 HorizeBlot 2 M, ATTO) at 24 V for 20 min. After blocking with Blocking One (Nacalai Tesque) for 30 min at room temperature, the membranes were incubated with an anti-MDA5 primary antibody (1:250 dilution, ab79055, Abcam) overnight at 4 °C. Following three washes with 0.1% Tween-20 in Tris-buffered saline (TBS-T), the membranes were incubated with a Goat anti-Rabbit IgG (H + L) Secondary Antibody (1:5000 dilution, 65-6120, Invitrogen) for 1 h at room temperature. Protein bands were visualized using Chemi-Lumi One Ultra (Nacalai Tesque), and chemiluminescent images were acquired using the FUSION Absolute imaging system (Vilber Bio Imaging). Due to the lack of appropriate internal standard proteins for BALF and CS fractions, equal protein loading was strictly ensured by the aforementioned precise protein quantification and initial cell count standardization.

### RNA sequencing

For RNA sequencing of CD11b^+^ macrophages or BMDMs, total RNA was extracted using the Nucleospin® RNA plus kit. The preparation of cDNA libraries with poly A selection and sequencing was performed for us by Macrogen Japan Inc. using an Illumina NovaSeq 6000 system (2 × 150 bp configuration, 6 Gb/sample). The quality score (Q30) was ≥95% in each sequenced sample. Trimming, mapping, calculating the abundance of transcripts as FPKM (fragments per kilobase of exon per million fragments mapped) values in each raw data, and analyzing DEGs were performed using the CLC genomics workbench. Heatmaps were generated using ComplexHeatmap while focusing on the 50 genes with the highest variance in the set, with a *P* value of <0.05 from the DEG analysis. Gene ontology and pathway analyses were performed using EnrichR (Chen et al, 2013; Kuleshov et al, 2016) or DAVID (Huang da et al, 2009a, b). The datasets have been deposited in the Gene Expression Omnibus database (GSE272953).

### Analysis of human samples on public databases

Gene expression profiling data of lung cells obtained by single-cell RNA-seq from healthy people (*n* = 10) and patients with ILD (*n* = 20, consisting of Idiopathic Pulmonary Fibrosis (IPF; *n* = 12), chronic hypersensitivity pneumonitis (cHP; *n* = 3), nonspecific interstitial pneumonia (NSIP; *n* = 2), sarcoidosis (*n* = 2), and unclassifiable ILD (*n* = 1)) were obtained from the public database (GSE135893) (Habermann et al, 2020). Cells annotated as immune cells in the paper were extracted and further categorized based on their expression profiles: *SPP1* (*SPP1*^+^ macrophages), *FABP4* (*FABP4*^+^ macrophages), *FCN* (monocytes), and *CCL17* (conventional dendritic cells, cDCs) (Morse et al, 2019) using the Seurat pipeline. Interactions between *SPP1*+ macrophages and monocytes were analyzed using CellphoneDB (Efremova et al, 2020), and only those interactions deemed significant (P < 0.05) were visualized using a circus plot. Microarray data on the expression analysis of BALF cells and relevant clinical data in healthy donors or patients with IPF were obtained (GSE70866, *n* = 20 and *n* = 175, respectively) (Prasse et al, 2019) from the public database. Gene expression profiling of patients with chronic lung disease from the Lung Genomics Research Consortium (GSE47460, 108 controls and 254 patients with ILD, including 160 patients with IPF) using lung homogenates and relevant clinical data was also obtained. Microarray data (GSE128314) (Cassius et al, 2020) on gene expression analysis using skin tissues from patients with anti-MDA5 autoantibody-associated (*n* = 3) and healthy donors (*n* = 5) were also analyzed to evaluate *CCL2*, *CCL7*, and *CCL8* expression. Correlations of *IFIH1* and each gene of interest were calculated. The clinical characteristics and demographics of the human subjects from the utilized public cohorts are summarized in Table EV1.

### Statistical analyses

Sample sizes were chosen based on standard practices in the field and our previous studies to provide sufficient statistical power. The exact number of biologically independent replicates (*n*) is indicated in the respective figure legends. For in vivo experiments, mice of similar age and body weight were randomly assigned to experimental groups. For in vitro experiments, cell cultures were randomly allocated to different treatment conditions. Investigators were blinded to the group allocation during histological evaluations and quantitative image analyses. No animals or samples were excluded from the analyses unless animals died prior to the predefined experimental endpoints due to procedure-related complications. All in vitro experiments were repeated three times independently. Student’s *t* test was used for the comparison between two groups. For comparisons among three or more groups, data were analyzed using regular one-way ANOVA followed by Tukey’s post hoc test. However, when the assumption of equal variances was not met, Welch’s ANOVA followed by Dunnett’s T3 multiple comparisons test was applied (GraphPad Prism 8). The Mann–Whitney test was performed for comparisons between two groups in human sample data. Spearman’s correlation coefficient (ρ) was calculated for correlation analysis. Statistical significance was set at *P* < 0.05. Data are expressed as means ± SEM.

## Supplementary information

Peer Review File

Table EV1

Source data Fig. 1

Source data Fig. 2

Source data Fig. 3

Source data Fig. 4

Source data Fig. 5

Source data Fig. 6

Source data Fig. 7

Figure EV1 Source Data

Figure EV2 Source Data

Figure EV3 Source Data

Figure EV4 Source Data

Figure EV5 Source Data

Expanded View Figures

## Data Availability

The RNA-Seq data reported in this paper have been deposited in NCBI’s Gene Expression Omnibus (GEO) database under accession number GSE272953. The source data of this paper are collected in the following database record: biostudies:S-SCDT-10_1038-S44321-026-00513-6.
