# Supplementary material for: Extracellular RNA clearance by non-canonical cell-surface MDA5 protects against lung fibrosis
Source: EMBO Mol Med. 2026 Oct 12;18(10):4001–33. doi: 10.1038/s44321-026-00513-6 (PMC13658825; doi:10.1038/s44321-026-00513-6)
Supplement: Supplementary file 1 [file 44321_2026_513_MOESM15_ESM.pdf]

Expanded View Figures

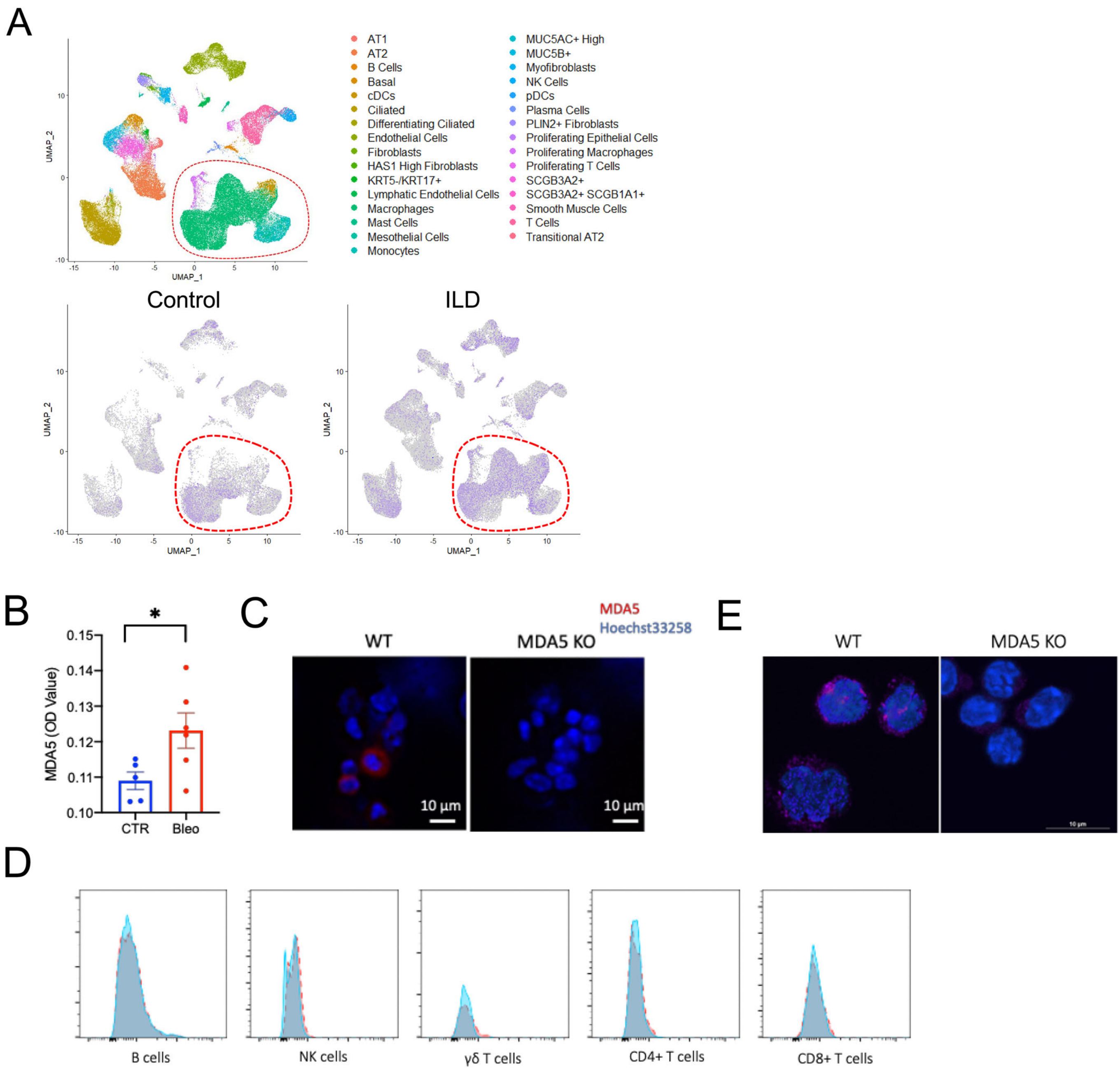

Figure EV1. Expression and dynamics of MDA5 in lung immune cells during fibrosis.

(**A**) Re-analysis of a single-cell RNA-sequencing dataset (GSE135893) from the lungs of healthy controls and patients with ILD. Upper panels show UMAP plots with new cell annotations. The red dashed circle indicates a population comprising macrophages, monocytes, and cDCs. Lower panels show the expression of *IFIH1* in this population. (**B**) U937 cells were treated with control or bleomycin for 24 h, and MDA5 levels in the supernatant were measured by ELISA (*P* value for CTR vs. Bleo: $P = 0.0409$). (**C**) Immunofluorescence staining for MDA5 (red) in single-cell suspensions from the lungs of WT and MDA5 KO mice. Nuclei were counterstained with Hoechst 33258 (blue). Scale bar, 10 μm; original magnification, ×200. (**D**) Representative flow cytometry histograms of surface MDA5 expression on lymphocyte subsets from bleomycin-treated WT (blue line) or MDA5 KO (red line, used as a negative control) mice. (**E**) Immunofluorescence staining of MDA5 (pink) in bleomycin-treated WT bone marrow-derived macrophages (BMDMs) with permeabilization using saponin. Hoechst 33258 was used for nuclear staining (blue). Scale bar, 10 μm; original magnification, ×600. Data in (**B**) is presented as mean ± SEM ($n = 3$–5 per group). $^{*}P < 0.05$, by Student's *t* test. Source data are available online for this figure

© The Author(s)

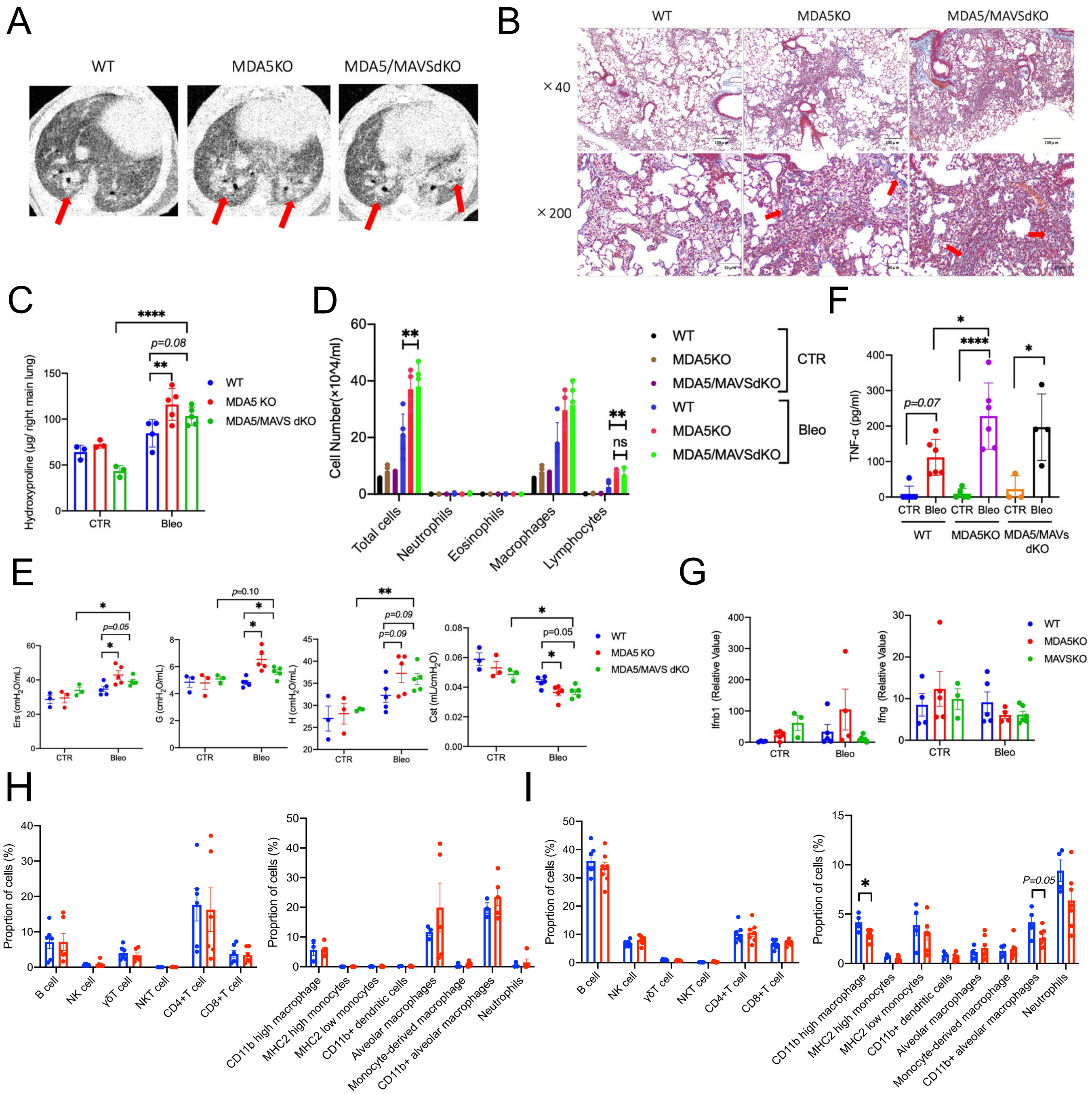

 © The Author(s)

Figure EV2. Inflammatory and fibrotic phenotype involving MDA5, MAVS, and IFNAR1.

(**A–F**) WT, MDA5 KO, and MDA5/MAVS double KO (dKO) mice were challenged with intratracheally administered bleomycin. (**A**) Representative micro-CT images of lungs on day 21. Arrows indicate areas of consolidation reflecting inflammation, edema, and fibrosis. (**B**) Representative lung sections stained with Masson's trichrome. Upper panels: original magnification, ×40; scale bar, 100 µm. Lower panels: original magnification, ×200; scale bar, 50 µm. (**C**) Hydroxyproline content in the right main lung (*P* values: MDA5/MAVS dKO Control vs. MDA5/MAVS dKO Bleomycin, $P < 0.0001$; WT Bleomycin vs. MDA5 KO Bleomycin, $P = 0.0033$). (**D**) Differential cell counts in BALF (*P* values for bleomycin-treated groups: total cells [WT vs. MDA5/MAVS dKO], $P = 0.0035$; lymphocytes [WT vs. MDA5/MAVS dKO], $P = 0.003$). ns not significant. (**E**) Lung mechanics, including elastance (Ers), tissue damping (G), tissue elastance (H), and static compliance (Cst), were measured using the flexiVent system. *P* values for Ers [WT Bleomycin vs. MDA5 KO Bleomycin, $P = 0.0144$; MDA5/MAVS dKO Control vs. MDA5/MAVS dKO Bleomycin, $P = 0.0455$]; G [WT Bleomycin vs. MDA5 KO Bleomycin, $P = 0.0202$; WT Bleomycin vs. MDA5/MAVS dKO Bleomycin, $P = 0.0391$]; H [WT Bleomycin vs. MDA5/MAVS dKO Bleomycin, $P = 0.0242$]; Cst [WT Bleomycin vs. MDA5 KO Bleomycin, $P = 0.0416$; MDA5/MAVS dKO Control vs. MDA5/MAVS dKO Bleomycin, $P = 0.0242$]). (**F**) TNF-α levels in the supernatants of control or bleomycin-treated alveolar macrophages derived from the indicated mice (*P* values: WT CTR vs. Bleo, $P = 0.07$; MDA5 KO CTR vs. Bleo, $P < 0.0001$; MDA5/MAVS dKO CTR vs. Bleo, $P = 0.011$; WT Bleo vs. MDA5 KO Bleo, $P = 0.0308$). (**G**) mRNA expression levels of *Ifnb1* (left) and *Ifng* (right) in whole lung homogenates from WT, MDA5 KO, and MAVS KO mice treated with saline (CTR) or bleomycin (Bleo), assessed by qPCR. (**H**, **I**) Flow cytometric analysis of lymphocyte (left) and other immune cell (right) populations in BALF (**H**) and lungs (**I**) from bleomycin-treated mice (*P* values in lungs (**I**) for MDA5 KO vs. IFNAR KO: CD11b high macrophages, $P = 0.0195$.). Blue, MDA5 KO; red, IFNAR KO. Data are presented as the mean ± SEM. *$P < 0.05$, **$P < 0.01$, ***$P < 0.001$, ****$P < 0.0001$, by one-way ANOVA with Tukey's post-hoc test or Welch's ANOVA followed by Dunnett's T3 multiple comparisons test (**E**, **F**). $n = 3$–5 for the control groups, $n = 4$–7 for the bleomycin groups. Source data are available online for this figure

© The Author(s)

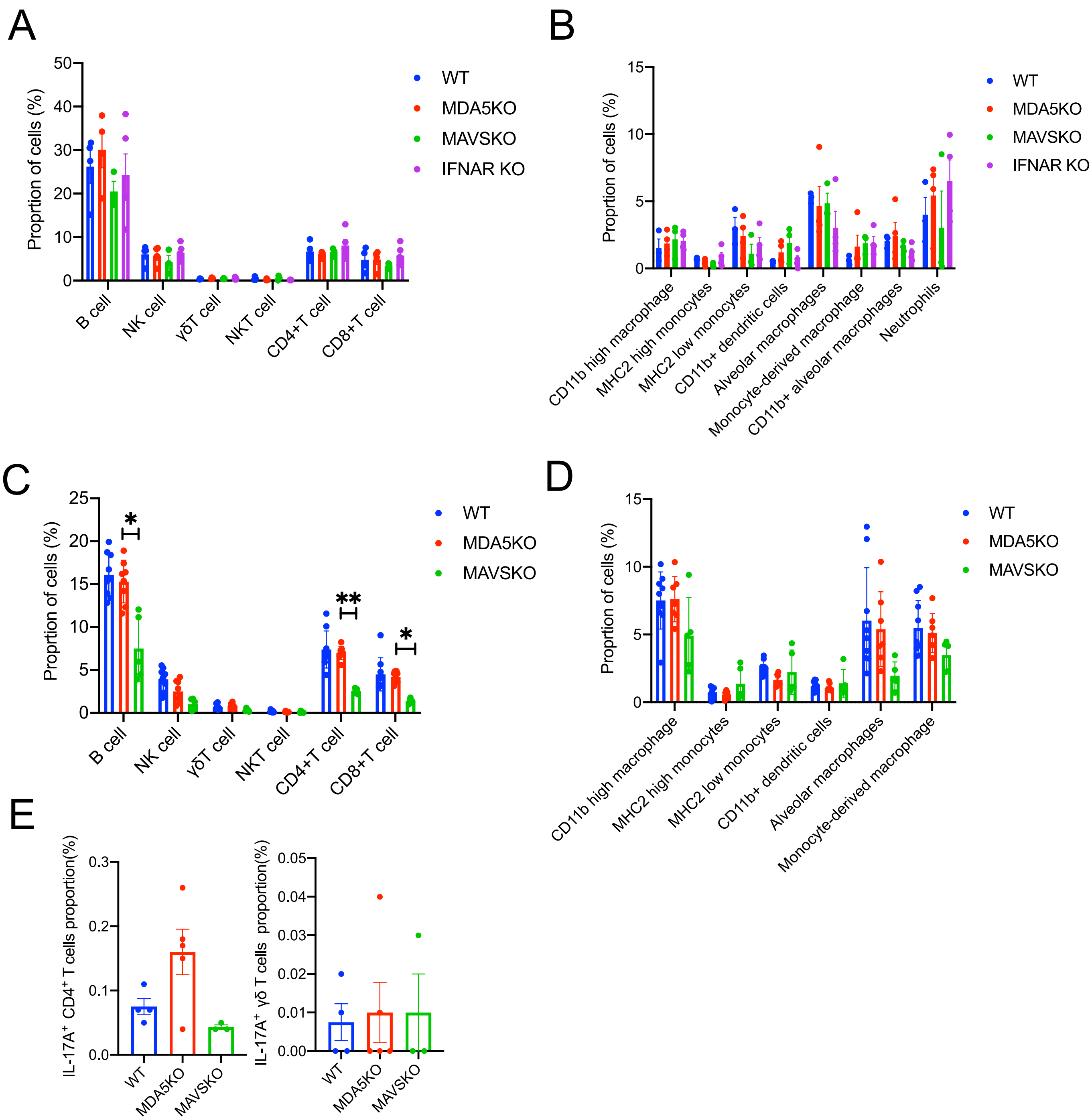

Figure EV3. Immune cell populations in the lungs of control- and bleomycin-treated mice.

(**A**, **B**) Flow cytometric analysis of lymphocyte (**A**) and other immune cell (**B**) populations in the lungs from saline-treated WT, MDA5 KO, MAVS KO, and IFNAR KO mice. (**C**, **D**) Proportions of specific lymphocyte populations (**C**) and other immune cell populations (**D**) in the lungs of bleomycin-treated WT, MDA5 KO, and MAVS KO mice, as determined by flow cytometry (exact *P* values for lymphocyte populations (**C**): B cells [MDA5 KO vs. MAVS KO], $P < 0.0001$; $CD4^+$ T cells [MDA5 KO vs. MAVS KO], $P < 0.0001$; $CD8^+$ T cells [MDA5 KO vs. MAVS KO], $P = 0.0032$). (**E**) The specific populations of IL-17A$^+$ T cells in the lungs of saline-treated WT, MDA5 KO, and MAVS KO mice. Data are presented as mean ± SEM. $^*P < 0.05$, $^{**}P < 0.01$, $^{***}P < 0.001$, $^{****}P < 0.0001$, by two-way ANOVA with Tukey's post-hoc test. $n = 3$–8 per group. Source data are available online for this figure

 © The Author(s)

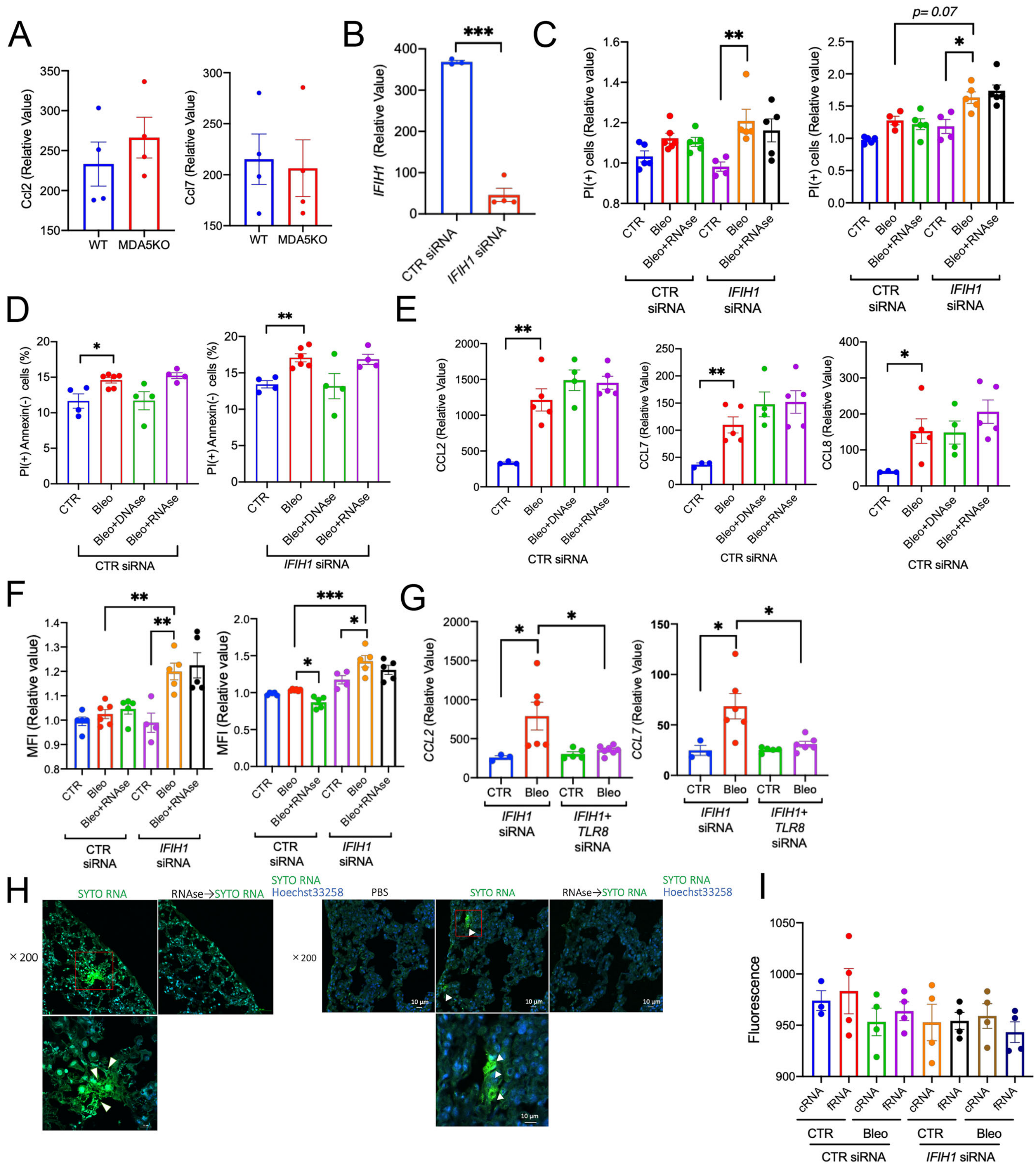
A
Ccl2 (Relative Value)
Ccl7 (Relative Value)
WT
MDA5KO
B
IFIH1 (Relative Value)
***
CTR siRNA
IFIH1 siRNA
C
PI(+) cells (Relative value)
p= 0.07
CTR
Bleo
Bleo+RNAse
CTR siRNA
IFIH1 siRNA
D
PI(+) Annexin(-) cells (%)
Bleo+DNAse
Bleo+RNAse
CTR siRNA
IFIH1 siRNA
E
CCL2 (Relative Value)
CCL7 (Relative Value)
CCL8 (Relative Value)
CTR siRNA
F
MFI (Relative value)
CTR siRNA
IFIH1 siRNA
G
CCL2 (Relative Value)
CCL7 (Relative Value)
IFIH1 siRNA
IFIH1+ TLR8 siRNA
H
SYTO RNA
RNAse→SYTO RNA
SYTO RNA Hoechst33258
PBS
×200
10 μm
I
Fluorescence
cRNA
fRNA
CTR
Bleo
CTR siRNA
IFIH1 siRNA

Figure EV4. In vitro validation of the MDA5–RNA–TLR8 axis.

(**A**) mRNA expression levels of *Ccl2* and *Ccl7* in PBS-treated BMDMs, assessed by qPCR. (**B**) *IFIH1* mRNA expression in U937 cells 24 h after transfection with control or *IFIH1* siRNA, measured by qPCR ($P < 0.0001$). (**C**) Proportions of PI-positive (necrotic) U937 cells 24 h (left) and 48 h (right) after stimulation. Cells were pre-treated with control or *IFIH1* siRNA, and then stimulated with PBS, bleomycin, or bleomycin + RNase (P value at 24 h: *IFIH1* siRNA [Control vs. Bleomycin], $P = 0.0090$; and similarly at 48 h: *IFIH1* siRNA [Control vs. Bleomycin], $P = 0.0133$). (**D**) U937 cells with or without *IFIH1* knockdown were treated with control, bleomycin, bleomycin + DNase, or bleomycin + RNase. The proportions of necrotic cells (Annexin-V^- PI^+) were evaluated (P values: CTR siRNA [Control vs. Bleomycin], $P = 0.0178$; *IFIH1* siRNA [Control vs. Bleomycin], $P = 0.0034$). (**E**) mRNA expression levels of *CCL2*, *CCL7*, and *CCL8* in U937 cells stimulated with PBS, bleomycin, bleomycin + DNase, or bleomycin + RNase (P values: *CCL2* [Control vs. Bleomycin, $P = 0.0025$]; *CCL7* [Control vs. Bleomycin, $P = 0.0086$]; *CCL8* [Control vs. Bleomycin, $P = 0.0299$]). (**F**) Cellular RNA levels in U937 cells, measured by flow cytometry. MFI of an RNA-specific dye is shown for PI-negative (live, left) and PI-positive (necrotic, right) cells 24 h post-stimulation, as in (**C**) (P values for live cells: *IFIH1* siRNA [Control vs. Bleomycin], $P = 0.0024$; Bleomycin [CTR siRNA vs. *IFIH1* siRNA], $p = 0.0056$. P values for necrotic cells: CTR siRNA [Bleomycin vs. Bleomycin + RNase], $P = 0.0001$; *IFIH1* siRNA [Control vs. Bleomycin], $P = 0.0367$; Bleomycin [CTR siRNA vs. *IFIH1* siRNA], $P < 0.0001$). (**G**) mRNA expression levels of *CCL2* (left) and *CCL7* (right) in U937 cells transfected with control, *IFIH1* siRNA, or *IFIH1* + *TLR8* siRNAs, and then stimulated with bleomycin (P values for *IFIH1* siRNA Control vs. *IFIH1* siRNA Bleomycin: *CCL2*, $P = 0.0303$; *CCL7*, $P = 0.0156$. Those for *IFIH1* siRNA Bleomycin vs. *IFIH1* + *TLR8* siRNA Bleomycin: *CCL2*, $P = 0.0232$; *CCL7*, $P = 0.0108$). (**H**) Left: High-magnification images of RNA staining in the bleomycin-treated lungs of MDA5 KO mice shown in Fig. 6F. Upper panels, original magnification, ×200. Lower panel, original magnification, ×600; scale bar, 10 µm. Right: RNA staining with SYTO RNAselect in lung tissues from WT mice treated with bleomycin (0.5 mg/kg) to induce severe lung fibrosis. Hoechst 33258 was used for nuclear staining. Images show treatments with PBS (left), SYTO RNA (middle), and SYTO RNA after RNase treatment (right). Upper panels, original magnification, ×200. Lower panel (SYTO RNA), original magnification, ×600; scale bar, 10 µm. Arrows indicate RNA depositions. (**I**) Fluorescence in the supernatants of U937 cells treated with control RNA (cRNA) or fluorescent RNA (fRNA) were measured. Data are presented as mean ± SEM. *$P < 0.05$, $P < 0.01$, ***$P < 0.001$, by Student's t test or one-way ANOVA. $n = 3$–6 per group. Source data are available online for this figure

© The Author(s)

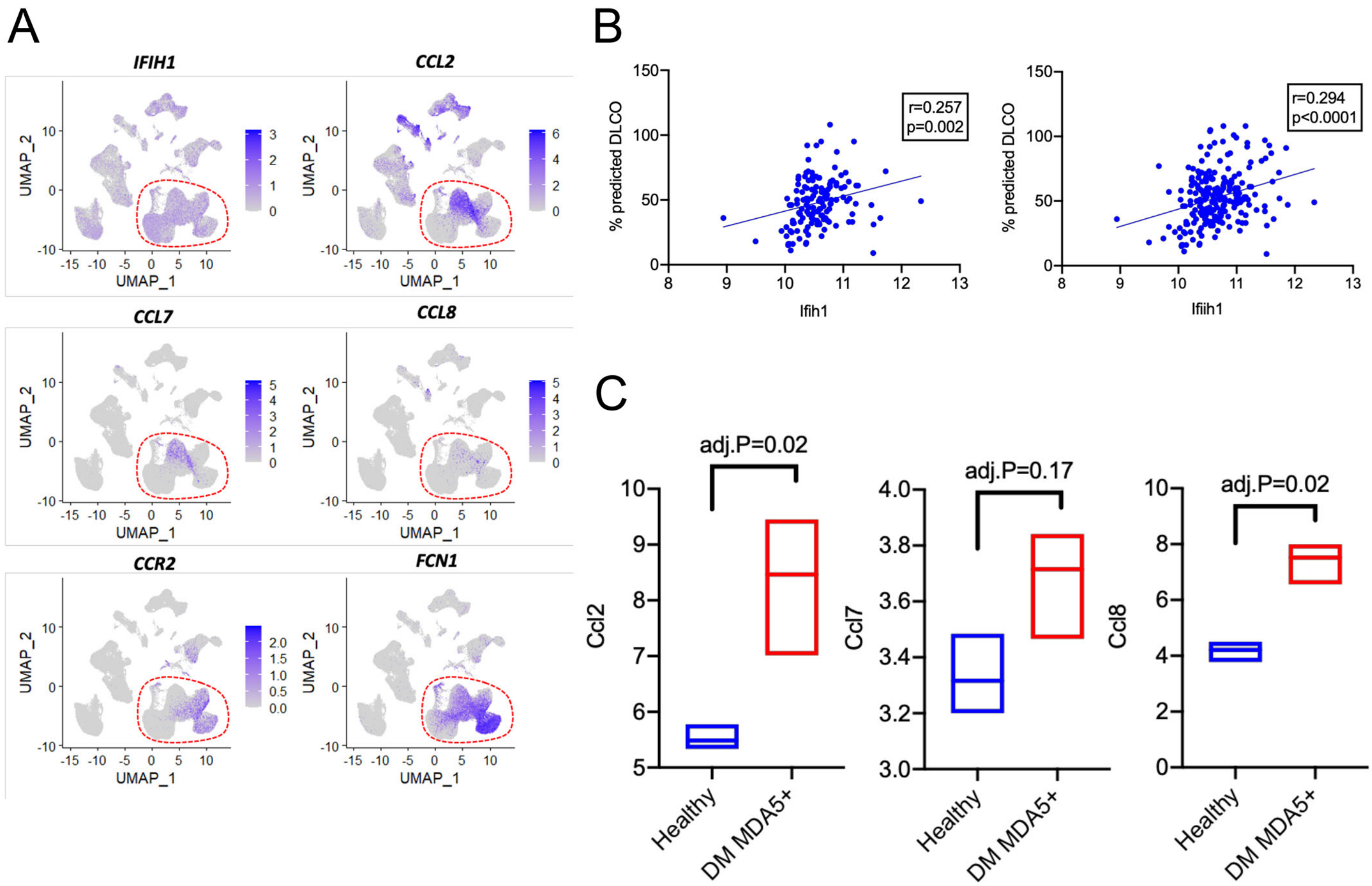

Figure EV5. Gene expression of *IFIH1* and related genes with clinical lung function.

(**A**) UMAP plots from a single-cell RNA-seq dataset (GSE135893) showing the expression of indicated genes in whole lung cells. The plot showing the expression of *IFIH1* is also shown in Fig. EV1A. The red dashed circle indicates the myeloid cell population. (**B**) Correlation between *IFIH1* expression and percent predicted DLCO (% predicted DLCO) in patients with IPF (left, $n = 128$) and patients with ILD including IPF (right, $n = 231$) from the Lung Genomics Research Consortium (GSE47460) (Spearman correlation: left [IPF], $r = 0.257$, $P = 0.002$; right [ILD], $r = 0.294$, $P < 0.0001$). The Spearman correlation coefficient (r) and P value are shown. (**C**) Microarray data showing the expression of *CCL2*, *CCL7*, and *CCL8* in skin tissue from healthy controls ($n = 6$) or patients with MDA5 antibody-positive dermatomyositis (DM MDA5 +, $n = 3$) (GSE128314). Box plots show the median and min/max values. Adjusted P values (adj.P) are shown. Source data are available online for this figure

© The Author(s)
