# Supplementary material for: Extracellular RNA clearance by non-canonical cell-surface MDA5 protects against lung fibrosis
Source: EMBO Mol Med. 2026 Oct 12;18(10):4001–33. doi: 10.1038/s44321-026-00513-6 (PMC13658825; doi:10.1038/s44321-026-00513-6)
Supplement: Supplementary file 2 [file 44321_2026_513_MOESM1_ESM.pdf]

EMBO Molecular Medicine

Extracellular RNA clearance by non-canonical cell-surface MDA5 protects against lung fibrosis.

Takashi Ishii, Masafumi Horie, Hirokazu Urushiyama, Yusuke Murakami, Tomoya Narita, Ryutaro Fukui, Kensuke Miyake, Akira Saito, Naomi Yamashita, Keiki Kumano, and Hidenori Kage

Corresponding author: Takashi Ishii (taishii-tky@umin.ac.jp, tkishii1123@gmail.com)

Review Timeline:

Submission Date:	14th Sep 25
Editorial Decision:	23rd Oct 25
Revision Received:	23rd May 26
Editorial Decision:	26th Jun 26
Revision Received:	27th Jul 26
Accepted:	19th Aug 26

Editor: Lise Roth

Transaction Report:

(Note: With the exception of the correction of typographical or spelling errors that could be a source of ambiguity, letters and reports are not edited. Depending on transfer agreements, referee reports obtained elsewhere may or may not be included in this compilation. Referee reports are anonymous unless the Referee chooses to sign their reports.)

23rd Oct 2025

Dear Dr. Ishii,

Thank you for submitting your manuscript to EMBO Molecular Medicine. Please accept my apologies for the delay in getting back to you as one of the referees needed more time to complete his/her review. We have now heard back from the three referees. As you will see below, the referees raise significant concerns about your manuscript, which unfortunately preclude its publication in EMBO Molecular Medicine in its current form.

The referees mention the interest of the findings, but also raise a number of partially overlapping major concerns that should be addressed to strengthen the conclusions and increase the robustness of the findings. After further cross-commenting with the referees, we agreed that experimental revisions should focus on:
- Providing adequate controls (MDA5 ko independent from bleomycin)
- Strengthening the mechanistic understanding regarding MAD5 secretion, MAVS independence and RNA degradation.

If you feel that you can satisfactorily address the points raised by the reviewers, you may wish to submit a revised version of your manuscript. Please include a covering letter detailing how you have addressed each of the points raised by the reviewers. A revised manuscript will be reviewed again, and we cannot guarantee at this stage that the final outcome will be favorable.

Revising the manuscript according to the reviewers' recommendations appears to require a lot of additional work and experimentation, and given the potential interest of your findings, we are prepared to extend the deadline to 6 months.

If you would like to discuss further the points raised by the referees or your revision plan, I am available to do so via email or video. Let me know if you are interested in this option.

EMBO Molecular Medicine encourages a single round of revision only and therefore, acceptance or rejection of the manuscript will depend on the completeness of your responses included in the next, final version of the manuscript. For this reason, and to save you from any frustrations in the end, I would strongly advise against returning an incomplete revision. Should you find that the requested revisions are not feasible within the constraints outlined here and prefer, therefore, to submit your paper elsewhere, we would welcome a message to this effect.

When submitting your revised manuscript, please carefully review the instructions that follow below. We perform an initial quality control of all revised manuscripts before re-review; failure to include requested items will delay the evaluation of your revision.

1) A .docx formatted version of the manuscript text (including legends for main figures, EV figures and tables). Please make sure that the changes are highlighted to be clearly visible.

2) Individual production quality figure files as .eps, .tif, .jpg (one file per figure). For guidance, download the 'Figure Guide PDF' (https://www.embopress.org/page/journal/17574684/authorguide#figureformat).

3) At EMBO Press we ask authors to provide source data for the main figures. Our source data coordinator will contact you to discuss which figure panels we would need source data for and will also provide you with helpful tips on how to upload and organize the files.

4) A .docx formatted letter INCLUDING the reviewers' reports and your detailed point-by-point responses to their comments. As part of the EMBO Press transparent editorial process, the point-by-point response is part of the Review Process File (RPF), which will be published alongside your paper.

5) A complete author checklist, which you can download from our author guidelines (https://www.embopress.org/page/journal/17574684/authorguide#submissionofrevisions). Please insert information in the checklist that is also reflected in the manuscript. The completed author checklist will also be part of the RPF.

6) All Materials and Methods need to be described in the main text using our 'Structured Methods' format. According to this format, the Methods section includes a Reagents and Tools Table (listing key reagents, experimental models, software and relevant equipment and including their sources and relevant identifiers) followed by a Methods and Protocols section describing the methods, ideally using a step-by-step protocol format. The aim is to facilitate adoption of the methodologies across labs. Please download and fill our Reagents and Tools Table template (.docx), which you can find in our author guidelines: https://www.embopress.org/page/journal/14693178/authorguide#structuredmethods.
When submitting your revised manuscript, please do not include the Reagents and Tools Table in the Methods section of the manuscript but upload it as a separate file choosing the file type "Reagent Table".
An example of a Method paper with Structured Methods can be found here:

https://www.embopress.org/doi/10.15252/msb.20178071

7) Please note that all corresponding authors are required to supply an ORCID ID for their name upon submission of a revised manuscript.

8) It is mandatory to include a 'Data Availability' section after the Materials and Methods. Before submitting your revision, primary datasets produced in this study need to be deposited in an appropriate public database, and the accession numbers and database listed under 'Data Availability'. Please remember to provide a reviewer password if the datasets are not yet public (see https://www.embopress.org/page/journal/17574684/authorguide#dataavailability).

In case you have no data that requires deposition in a public database, please state so in this section. Note that the Data Availability Section is restricted to new primary data that are part of this study.

9) For data quantification: please specify the name of the statistical test used to generate error bars and P values, the number (n) of independent experiments (specify technical or biological replicates) underlying each data point and the test used to calculate p-values in each figure legend. The figure legends should contain a basic description of n, P and the test applied. Graphs must include a description of the bars and the error bars (s.d., s.e.m.). Please provide exact p values.

10) Our journal encourages inclusion of *data citations in the reference list* to directly cite datasets that were re-used and obtained from public databases. Data citations in the article text are distinct from normal bibliographical citations and should directly link to the database records from which the data can be accessed. In the main text, data citations are formatted as follows: "Data ref: Smith et al, 2001" or "Data ref: NCBI Sequence Read Archive PRJNA342805, 2017". In the Reference list, data citations must be labeled with "[DATASET]". A data reference must provide the database name, accession number/identifiers and a resolvable link to the landing page from which the data can be accessed at the end of the reference. Further instructions are available at .

11) We replaced Supplementary Information with Expanded View (EV) Figures and Tables that are collapsible/expandable online. EV Figures should be cited as 'Figure EV1, Figure EV2" etc... in the text and their respective legends should be included in the main text after the legends of regular figures.
- For the figures that you do NOT wish to display as Expanded View figures, they should be bundled together with their legends in a single PDF file called *Appendix*, which should start with a short Table of Content. Appendix figures should be referred to in the main text as: "Appendix Figure S1, Appendix Figure S2" etc.
- Additional Tables/Datasets should be labeled and referred to as Table EV1, Dataset EV1, etc. Legends have to be provided in a separate tab in case of .xls files. Alternatively, the legend can be supplied as a separate text file (README) and zipped together with the Table/Dataset file.
See detailed instructions here:

12) The paper explained: EMBO Molecular Medicine articles are accompanied by a summary of the articles to emphasize the major findings in the paper and their medical implications for the non-specialist reader. Please provide a draft summary of your article highlighting
- the medical issue you are addressing,
- the results obtained and
- their clinical impact.
This may be edited to ensure that readers understand the significance and context of the research. Please refer to any of our published articles for an example.

13) Author contributions: CRediT has replaced the traditional author contributions section because it offers a systematic machine readable author contributions format that allows for more effective research assessment. Please remove the Authors Contributions from the manuscript and use the free text boxes beneath each contributing author's name in our system to add specific details on the author's contribution. More information is available in our guide to authors.

14) Disclosure statement and competing interests: We updated our journal's competing interests policy in January 2022 and request authors to consider both actual and perceived competing interests. Please review the policy https://www.embopress.org/competing-interests and update your competing interests if necessary.

15) Every published paper now includes a 'Synopsis' to further enhance discoverability. Synopses are displayed on the journal webpage and are freely accessible to all readers. They include a short stand first (maximum of 300 characters, including space) as well as 2-5 one-sentences bullet points that summarizes the paper. Please write the bullet points to summarize the key NEW findings. They should be designed to be complementary to the abstract - i.e. not repeat the same text. We encourage inclusion of key acronyms and quantitative information (maximum of 30 words / bullet point). Please use the passive voice. Please attach these in a separate file or send them by email, we will incorporate them accordingly.

Please also suggest a visual abstract to illustrate your article as a PNG file 550 px wide x 300-600 px high. A cropped portion of

this image will serve as thumbnail for the table of content on our webpage.

16) As part of the EMBO Publications transparent editorial process initiative (see our Editorial at http://embomolmed.embopress.org/content/2/9/329), EMBO Molecular Medicine will publish online a Review Process File (RPF) to accompany accepted manuscripts.
In the event of acceptance, this file will be published in conjunction with your paper and will include the anonymous referee reports, your point-by-point response and all pertinent correspondence relating to the manuscript. Let us know whether you agree with the publication of the RPF and as here, if you want to remove or not any figures from it prior to publication.
Please note that the Authors checklist will be published at the end of the RPF.

EMBO Molecular Medicine has a "scooping protection" policy, whereby similar findings that are published by others during review or revision are not a criterion for rejection. Should you decide to submit a revised version, I do ask that you get in touch after six months if you have not completed it, to update us on the status.

I look forward to receiving your revised manuscript.

Yours sincerely,

Lise Roth

Lise Roth, PhD
Senior Editor
EMBO Molecular Medicine

***** Reviewer's comments *****

Referee #1 (Comments on Novelty/Model System for Author):

limited in vitro human cell systems and requirements for an additional KO mouse line (IFNAR KO)

Referee #1 (Remarks for Author):

Ishii et al. explore the role of the innate RNA sensor MDA5 in bleomycin-induced lung fibrosis. Using genetic knockout mouse models, they show that MDA5, particularly in macrophages, relocalizes to the plasma membrane upon bleomycin treatment, coinciding with the accumulation of RNA. MDA5-deficient mice displayed exacerbated inflammation and fibrosis, phenotypes absent in MAVS-deficient mice but retained in MDA5/MAVS double knockouts, suggesting a non-canonical, MAVS-independent mechanism. Consistent with this, BALF and lung cell profiling revealed elevated IL-17 (likely from γδT cells), increased neutrophil activation, and enrichment of fibrosis-related transcripts in macrophages. In vitro, MDA5-deficient mouse BMDMs and human U937 cells showed upregulated CCR2-activating chemokines, and CCR2 blockade ameliorated disease in vivo. Furthermore, MDA5 silencing enhanced cell death and CCL expression in U937 cells, which was reduced by RNase or TLR8 silencing, pointing to possible crosstalk. Lastly, correlative analyses in ILD, IPF, and anti-MDA5 autoantibody-positive dermatomyositis datasets revealed a link between CCL expression, CCR2 signalling, and MDA5. While the study raises an interesting and clinically relevant question and the idea of extracellular MDA5 contributing to sterile inflammation is intriguing, the data presented remain largely descriptive and the mechanistic model is highly speculative, with several key conclusions lacking sufficient experimental validation. The manuscript is also poorly organized, which makes it difficult to follow the central hypothesis.

Major points:

1. More rigorous experimental approaches such as cell fractionation and super-resolution microscopy are needed to substantiate the claim that MDA5 redistributes to the plasma membrane following bleomycin treatment. To this reviewer, the data suggest that intracellular MDA5, itself an ISG, is clearly upregulated by bleomycin, as indicated in the RNA-seq datasets (Figs 3F and 4A). However, in Fig 1D, when comparing control and bleomycin-treated conditions in the MDA5 KO group, a similar shift as WT

is observed, and the differences between WT and KO appear marginal. Antibody staining also appears comparable in WT and KO cells under control conditions (Fig 6D), and the reported membrane localization upon bleomycin treatment resembles a general increase in staining intensity throughout the cell (Fig S1C and Fig 6D).

2. It is unclear why bleomycin treatment reduced MDA5 levels in BALF and sera (Figs 1B and S1B) while at the same time being associated with enhanced membrane anchoring. A clear explanation is needed to reconcile these findings. An alternative possibility is that the observed staining reflects MHC-I presentation of MDA5-derived peptides rather than true membrane relocalization. In addition, the proposed mechanism by which MDA5 mediates self-RNA clearance is not addressed experimentally and remains only suggested in the title. At a minimum, colocalization studies of RNA and MDA5 would be required to begin to interrogate this mechanism.

3. In Fig 2D, IL-17A also appears upregulated in MAVS KO cells, which seems inconsistent with the statement in lines 142-143. This discrepancy should be clarified.

4. An important aspect that remains to be addressed is the contribution of interferon responses to the development of fibrosis. In Fig S2A, bleomycin treatment induced noticeably higher IFN-beta levels in MDA5 KO cells, which may reflect compensation by alternative RNA sensors such as TLR8. Conversely, the RNA-seq data in Fig 3F show that MDA5 deficiency blunted IFN responses, as indicated by downregulation of multiple ISGs, suggesting that the canonical MDA5-MAVS axis remains functional under bleomycin treatment. The seemingly opposing effects on fibrotic gene induction may therefore arise from the action of alternative sensors in the absence of a negative feedback loop normally mediated by MDA5-driven IFN responses. A similar mechanism could underlie the regulation of CCL expression, which depends on NF-kappaB activation and may be exaggerated when MDA5-dependent negative feedback is absent. The inclusion of IFNAR KO mice in the study would help clarify these possibilities and disentangle the contribution of IFN signalling from other pathways.

5. In Fig 4D, MDA5 KO cells appear to display elevated baseline levels of CCL expression even prior to treatment. This raises the question of why the knockout condition itself induces such an effect. Without clarifying this baseline difference, it becomes difficult to interpret the extent to which bleomycin specifically upregulates CCL expression.

6. A model diagram that integrates the relevant cell types and molecular events underlying the pathogenesis of lung fibrosis would substantially improve clarity and help readers better grasp the proposed central mechanism.

Referee #2 (Comments on Novelty/Model System for Author):

Overall, a good and needed work. Some care should be taken of the results obtained re-using publicly available data.

Referee #2 (Remarks for Author):

With real pleasure and interest, I read the manuscript entitled "The RNA sensor MDA5 protects against lung fibrosis by clearing extracellular self-RNA" (manuscript ID: EMM-2025-22609), written by Ishii and colleagues. This nicely written work is based on a solid study performed by renowned researchers. Furthermore, the topic is intriguing and innovative. As a result, I have only a few comments, mostly minor and/or facultative:

1. The Authors quite effectively use publicly available datasets. Usually, I am against a massive usage of such data, and I accept only initial, fishing expedition, replication, or data expansion usage if no other option is available. In this very case, the Authors used publicly data at the very beginning of their work, while starting their investigations, and to expand their results by inclusion of data gathered in human cohorts. In addition, the amount of the public data used is rather well counterbalanced by their Authors' own data, which make a huge majority of the manuscript.
Therefore, I am fine with application of publicly available datasets in this work. Still, I would recommend that the Authors widely discuss usage of publicly available data as a limitation of the study in the respective part of the discussion section.
2. Please, make it clear also in the abstract which parts of the data derive from your won experiments, and which were obtained by re-usage of publicly available datasets.
3. Results obtained by re-analysis of publicly available datasets should be accompanied by clear description of biomaterials used (cells, tissues, etc.). In case of human data, characteristics of the patients should be given in the supplement.
4. Another limiting factor is lack of Authors' own data obtained in primary human cells and/or tissues, especially using modern in vitro/ex vivo such as organoids, air-liquid interface and/or others (PMID: 37626876, 37770004). Inclusion of such data would substantially further increase the value of this work. If such data are not available, please, at least discuss as a limitation of the study.
5. NETs. Please, expand on the role of NETs in physiology and pathology (PMID: 32663035, 36257987). Please, mention some other disorders in which NETosis plays a crucial role (PMID: 39512344, 38928183, etc.).
6. Please, consider further uniformization and unification of quantitative data presentation between the figures.
7. I am sorry if I overlooked something, but I was not able to see a graphical abstract. I would recommend adding it.

Referee #3 (Remarks for Author):

General Comments:
Ishii et al. present an original manuscript looking at the RNA sensor MDA5 in protecting against lung fibrosis by clearing extracellular self-RNA. This study looks at MDA5, a cytosolic RNA sensor, in lung fibrosis through use of transgenic MDA5 KO mice and in vitro cell culture systems. After bleomycin, KO mice had exacerbated fibrosis with increased IL-17A gd T cell and neutrophil infiltration, suggesting MDA5 has a protective role independent of MAVS pathway. Mechanistically, authors suggest MDA5 contributes to clearance of extracellular RNA, thereby suppressing macrophage CCR2 ligand expression in a TLR8-dependent manner. Lung macrophages from MDA5 KO mice (resemble SPP1+ mac in humans), show elevated CCR2 ligands. The KO phenotype is interesting and convincing, but the mechanisms proposed are vague and not as convincing because of the global nature of the knockout, lack of cell specific KO mice, and lack of controls showing the impact of MDA5 KO without bleomycin. Also, the link to ILD is confusing, because MDA5+ antibodies are circulating, not MDA5 itself. It is not clear what MDA5 itself does in myositis ILD, nor is it clear whether antibodies are present in bleomycin induced lung fibrosis. The authors do not look at MDA5 antibodies in any of their studies. Overall, while the study is interesting, there are some major limitations that should be addressed.
Major Comments:
1. The global nature of MDA5 KO makes it difficult to tease apart the specific cellular contributions to the exacerbated phenotype. The authors should consider a more cell-specific targeting strategy to determine the cells involved in MDA5 expression.
2. There is not a clear mechanism which explains specifically how MDA5 would prevent lung fibrosis through regulation of RNA degradation. There is no evidence showing clearance, many mechanisms regulate extracellular deposition of RNA, could also just be due to lack of binding, shifting of binding, re-localization, or increased cell death. It is also not clear how MDA5 gets to the cell membrane of what the significance of this is in the development of disease. Additionally, MDA5 itself has no RNase activity.
3. KO of MDA5 causes apoptosis and increased inflammation, which may have nothing to do specifically with fibrosis or RNA clearance. This is not considered in the manuscript.
4. In Figure 1B, only levels of MDA5 in BAL are shown pre/post bleomycin, but not in BAL from MDA5 KO mice both with and without bleomycin, the mice should lose the effect if specific to MDA5. This is an important control.
5. In Figure 1E, changes in fluorescence are not equivalent to RNA clearance, and are not specific to degradation and are also not quantitative.
6. In Figure 1E, lung tissue is shown after bleomycin showing RNA in the cytoplasm and extracellular spaces, without histology staining it is not clear whether these are fibrotic areas. Adjacent sections of HE or trichrome should be shown for context. Also, authors should show this experiment in KO mice not treated with bleomycin as an important control.
7. In Figure 3 (and throughout the paper), there is a lack of important controls showing that the immune phenotype is not induced just by MDA5 KO, independent of bleomycin. This would be clearer if they looked at MDA5 KO with and without bleomycin. This holds true both in mice and in vitro KO as well.
8. Use of BMDMs from mice may not translate into lung specific macrophage findings. In addition, direct exposure to bleomycin may not represent ILD or even bleomycin in vivo. RNAseq data therefore may not relate to lungs. Human validation with monocyte cell line helps, but again bleomycin used rather than profibrotic cytokines.
9. There is increased surface distribution of MDA5 with bleomycin, but it is not explained why or how this occurs.
10. It may be that increased RNA is released after bleomycin in MDA5 KO mice and cells, but this is not quantified, and it could be due to increased cell apoptosis releasing RNA, this is not considered, and cell death itself is a relatively non-specific mechanism.
11. The authors link MDA5 to ILD, but the link is not clear, there are no measurements of MDA5 antibodies in the models, or levels of MDA5 in circulation from ILD patients.
Minor Comments:
1. In Figure 1C, the MDA5 KO mouse histology does not look worse than WT, although OHP shows it is more fibrotic in Figure 2. It would be good to use representative images that match other figures quantification.
2. In Figure 2A, authors report CT scan shows worse disease in MDA5 KO, but invasive shadows and GGOs are very non-specific, and could represent edema, inflammation or fibrosis. This should be explained.
3. The analysis of scRNAseq is vague, "some structural cells", "patients with ILDs" there are so many questions here like what cells are being compared and what ILDs are being compared.

Referee #1 (Remarks for Author):

Ishii et al. explore the role of the innate RNA sensor MDA5 in bleomycin-induced lung fibrosis. Using genetic knockout mouse models, they show that MDA5, particularly in macrophages, relocalizes to the plasma membrane upon bleomycin treatment, coinciding with the accumulation of RNA. MDA5-deficient mice displayed exacerbated inflammation and fibrosis, phenotypes absent in MAVS-deficient mice but retained in MDA5/MAVS double knockouts, suggesting a non-canonical, MAVS-independent mechanism. Consistent with this, BALF and lung cell profiling revealed elevated IL-17 (likely from γδT cells), increased neutrophil activation, and enrichment of fibrosis-related transcripts in macrophages. In vitro, MDA5-deficient mouse BMDMs and human U937 cells showed upregulated CCR2-activating chemokines, and CCR2 blockade ameliorated disease in vivo. Furthermore, MDA5 silencing enhanced cell death and CCL expression in U937 cells, which was reduced by RNase or TLR8 silencing, pointing to possible crosstalk. Lastly, correlative analyses in ILD, IPF, and anti-MDA5 autoantibody-positive dermatomyositis datasets revealed a link between CCL expression, CCR2 signalling, and MDA5. While the study raises an interesting and clinically relevant question and the idea of extracellular MDA5 contributing to sterile inflammation is intriguing, the data presented remain largely descriptive and the mechanistic model is highly speculative, with several key conclusions lacking sufficient experimental validation. The manuscript is also poorly organized, which makes it difficult to follow the central hypothesis.

We thank Referee #1 for the constructive and insightful comments on our manuscript. We sincerely apologize for the lack of clarity and the organizational shortcomings in our initial submission. We have taken this comment very seriously and have thoroughly reorganized the structure of the manuscript to ensure a logical flow that clearly highlights our central hypothesis. We have significantly revised the manuscript and added several new sets of experimental data (including super-resolution microscopy, biochemical cell-surface protein isolation, and in vivo experiments using IFNAR KO mice) to address the reviewer's concerns rigorously. Below are our point-by-point responses to the major points raised.

Major points:

1. More rigorous experimental approaches such as cell fractionation and super-resolution microscopy are needed to substantiate the claim that MDA5 redistributes to the plasma membrane following bleomycin treatment. To this reviewer, the data suggest that intracellular MDA5, itself an ISG, is clearly upregulated by bleomycin, as indicated in the RNA-seq datasets (Figs 3F and 4A). However, in Fig 1D, when comparing control and bleomycin-treated conditions in the MDA5 KO group, a similar shift as WT is observed, and the differences between WT and KO appear marginal. Antibody staining also appears comparable in WT and KO cells under control conditions (Fig 6D), and the reported membrane localization upon bleomycin treatment resembles a general increase in staining intensity throughout the cell (Fig S1C and Fig 6D).

Response: We fully agree with the Reviewer that rigorous experimental approaches are required to substantiate the cell-surface translocation of MDA5. To provide definitive biochemical and spatial evidence, we have performed several additional experiments as suggested:

1. **Biochemical Isolation of Cell-Surface Proteins (Fig. 1F):** We isolated cell-surface proteins from U937 cells using a cell-surface biotinylation and isolation kit. Western blot analysis of these purified cell-surface fractions clearly demonstrated that bleomycin stimulation substantially increased the amount of MDA5 anchored to the plasma membrane, providing direct biochemical evidence independent of imaging techniques [Page 8, Lines 134-140; Fig. 1F].
2. **Inhibition of Vesicular Transport (Fig. 1G):** To elucidate the mechanism of this translocation, we pretreated cells with Brefeldin A (BFA), a classical inhibitor of ER-to-Golgi vesicular transport. BFA treatment completely abolished the bleomycin-induced surface translocation of MDA5, confirming that this redistribution is an active, regulated secretory process rather than a passive artifact of overall cellular upregulation [Page 8, Lines 141-145; Fig. 1G].
3. **Super-Resolution Microscopy (Fig. 6E):** As suggested, we performed super-resolution microscopy (using the Nikon NSPARC system) on bleomycin-treated bone marrow-derived macrophages (BMDMs) without cell permeabilization. This approach clearly visualized the precise, punctate localization of MDA5 clustered at the cell periphery (plasma

membrane) [Page 16-17, Lines 340-346; Fig. 6E].

Regarding the Reviewer's critical note on flow cytometry (Fig. 1D) and immunofluorescence (now Fig. 1E and Appendix Fig. S1D), we would like to emphasize that these experiments were conducted without cell permeabilization. The image of Immunofluorescence staining with permeabilization is also shown in Appendix Fig. S1E. Therefore, the detected signals exclusively represent the cell-surface-exposed pool, not the abundant intracellular MDA5. While a minor baseline signal shift can occur due to slight variations in background autofluorescence under stress, the significant, robust increase in surface signal upon bleomycin treatment is specific to WT cells. The baseline-like, flat profile maintained in MDA5 KO cells validates both the specificity of our antibody and our non-permeabilized protocol. We believe these multi-pronged, rigorous datasets now firmly support our model of active MDA5 translocation under cellular stress.

2. It is unclear why bleomycin treatment reduced MDA5 levels in BALF and sera (Figs 1B and S1B) while at the same time being associated with enhanced membrane anchoring. A clear explanation is needed to reconcile these findings. An alternative possibility is that the observed staining reflects MHC-I presentation of MDA5-derived peptides rather than true membrane relocalization. In addition, the proposed mechanism by which MDA5 mediates self-RNA clearance is not addressed experimentally and remains only suggested in the title. At a minimum, colocalization studies of RNA and MDA5 would be required to begin to interrogate this mechanism.

Response: Regarding the dynamic inverse correlation between BALF levels and cell-surface anchoring of MDA5, we offer the following result-based explanation and discussion in our revised manuscript: In the steady state (control condition), lung macrophages constitutively release a certain amount of MDA5 into the extracellular space (BALF). However, upon tissue injury induced by bleomycin, cellular dynamics shift to adapt to the stress environment. Our results suggest that under such conditions, MDA5 actively translocates to and is retained on the plasma membrane via the ER-Golgi pathway (as shown by BFA sensitivity in Fig. 1G) to function as an anchored scavenger receptor. Consequently, the shedding or free release of MDA5 into the BALF decreases. Additionally, since our BALF analysis was conducted at a single time point (2 weeks post-bleomycin), this

reduction may also reflect the specific time-course kinetics of extracellular protein distribution during the subacute phase of inflammation. Furthermore, our direct biochemical evidence using whole-protein cell-surface isolation (Fig. 1F) detects full-length MDA5 (approx. 140 kDa), ruling out the possibility that the observed membrane signal is merely due to the presentation of small MDA5-derived peptides on MHC class I.

Regarding the experimental validation of the self-RNA clearance mechanism, we have performed extensive new functional assays to fully support the hypothesis suggested in the title:

1. **Colocalization Studies (Fig. 6E):** Using super-resolution microscopy without cell permeabilization, we have successfully demonstrated the precise spatial colocalization of MDA5 and RNA signals at the cellular periphery of BMDMs, proving their physical interaction on the cell surface [Page 16-17, Lines 340-346; Fig. 6E].
2. **Quantitative In Vitro Clearance Assay (Fig. 6G):** We fed cells with fluorescently labeled lung tissue-derived self-RNA. While control siRNA-treated U937 cells properly processed and cleared the internalized RNA, *IFIH1*(MDA5) knockdown led to an abnormal, massive accumulation of intracellular RNA that was resistant to external RNase degradation, demonstrating a fundamental defect in extracellular RNA turnover [Page 17-18, Lines 355-366; Fig. 6G].
3. **Lysosomal Degradation Pathway (Fig. 6H–J):** Treatment with Chloroquine (CQ), a lysosomal inhibitor, dose-dependently mimicked the MDA5-deficient phenotype by accumulating fluorescent RNA inside the cells and causing uninternalized RNA to stagnate on the cell surface [Page 18, Lines 367-374; Fig. 6H–J].

Together, these results provide solid, direct experimental evidence that MDA5 physically binds extracellular self-RNA at the cell surface and shuttles it into the lysosomal degradation pathway.

3. In Fig 2D, IL-17A also appears upregulated in MAVS KO cells, which seems inconsistent with the statement in lines 142-143. This discrepancy should be clarified.

Response: We appreciate the Reviewer's careful evaluation of the whole-lung

qPCR data (Fig. 2D). We agree that the description of MAVS KO mice in the original text required further clarification to avoid misunderstanding.

While the whole-lung qPCR (Fig. 2D) captured a statistically significant baseline-to-bleomycin increase in *Il17a* transcript within the MAVS KO group, the absolute induction levels remained statistically comparable to the WT group, and were noticeably lower than the massive upregulation seen in MDA5 KO lungs.

To resolve this nuance at the single-cell level, we characterized specific immune cell profiles:

- **Baseline T-cell Proportions (Appendix Fig. S3E):** In the steady state (saline control), there was no significant difference in the baseline proportion of IL-17A-producing T cells among WT, MDA5 KO, and MAVS KO mice.
- **Bleomycin-induced T-cell Responses (Fig. 3B):** Following bleomycin treatment, the proportions of lung-infiltrating IL-17A$^+$ T cells, and specifically pathogenic IL-17A$^+$ γδ T cells, were significantly lower in MAVS KO mice compared to MDA5 KO mice, remaining at levels equivalent to WT mice.

Therefore, the profound activation of the pathogenic IL-17A$^+$ γδ T cell–neutrophil axis is uniquely specific to MDA5 deficiency. The mild increase observed in the whole-lung qPCR of MAVS KO mice likely originates from alternative, non-pathogenic resident cell types that do not contribute significantly to the core fibrotic phenotype. We have revised our text to describe these cellular nuances more accurately [Page 11, Lines 203-216].

4. An important aspect that remains to be addressed is the contribution of interferon responses to the development of fibrosis. In Fig S2A, bleomycin treatment induced noticeably higher IFN-beta levels in MDA5 KO cells, which may reflect compensation by alternative RNA sensors such as TLR8. Conversely, the RNA-seq data in Fig 3F show that MDA5 deficiency blunted IFN responses, as indicated by downregulation of multiple ISGs, suggesting that the canonical MDA5-MAVS axis remains functional under bleomycin treatment. The seemingly opposing effects on fibrotic gene induction may therefore arise from the action of alternative sensors in the absence of a negative feedback loop normally mediated by MDA5-driven IFN responses. A similar mechanism could underlie the regulation of CCL expression, which depends on NF-kappaB activation and may

be exaggerated when MDA5-dependent negative feedback is absent. The inclusion of IFNAR KO mice in the study would help clarify these possibilities and disentangle the contribution of IFN signaling from other pathways.

Response: We highly appreciate this insightful mechanistic hypothesis regarding the potential role of an MDA5-driven type I IFN negative feedback loop. To definitively disentangle the contribution of type I IFN signaling from the MDA5-mediated proinflammatory pathway, we newly included IFNAR KO mice in our study and conducted comprehensive *in vivo* and *in vitro* comparative analyses:

- **Phenotypic and Molecular Divergence In Vivo (Fig. 2G–I):** Consistent with previous literature, IFNAR KO mice displayed exacerbated lung fibrosis (histopathology and hydroxyproline content) partially comparable to MDA5 KO mice, confirming the general protective roles of baseline type I IFN. However, their underlying molecular profiles were fundamentally distinct. Crucially, unlike the massive chemokine surge observed in MDA5 KO mice, the induction of the key pro-fibrotic chemokine *Ccl2* and the classic ISG *Mx1* was significantly suppressed in IFNAR KO lungs [Page 10 Lines 185-199].
- **Distinct Inflammatory Landscapes (Appendix Fig. S2H):** Detailed flow cytometric profiling revealed that although the baseline profile of lung immune cells was comparable between MDA5 KO and IFNAR KO mice (Appendix Fig. S3A, B), the frequencies of pathogenic lung CD11b^(high) macrophages and $CD11b^+$ alveolar macrophages were notably lower in IFNAR KO mice than in MDA5 KO mice, highlighting distinct pathogenic cellular mechanisms [Page 10, Lines 185-199; Appendix Fig. S2H].
- **In Vitro Chemokine Regulation (Fig. 4F):** In BMDMs, the exaggerated induction of *Ccl2* and *Mx1* seen in MDA5 KO cells upon bleomycin treatment was significantly blunted in IFNAR KO BMDMs [Page 13, Lines 268-270; Fig. 4F].

These new findings demonstrate that the hyper-activation of the CCR2-ligand axis observed in MDA5 deficiency cannot be explained by the loss of an IFN-dependent negative feedback loop, because direct disruption of IFN signaling (IFNAR KO) actually suppresses rather than exaggerates CCL2 production. Instead, these data strongly reinforce our model: MDA5 deficiency leads to a primary defect in extracellular RNA clearance, which directly over-activates an alternative endosomal sensor—specifically TLR8—driving the pro-fibrotic CCR2

ligand cascade independent of canonical type I IFN signaling. We have expanded our Discussion to address these points extensively [Page 22, Lines 443-455].

.

5. In Fig 4D, MDA5 KO cells appear to display elevated baseline levels of CCL expression even prior to treatment. This raises the question of why the knockout condition itself induces such an effect. Without clarifying this baseline difference, it becomes difficult to interpret the extent to which bleomycin specifically upregulates CCL expression.

Response: We understand the Reviewer's concern regarding the baseline interpretation of CCL expression. To address this rigorously, we performed additional precise baseline validation experiments in BMDMs.

As now demonstrated in our new Appendix Fig. S4A, after careful re-examination with expanded sample sizes, the baseline expression levels of both *Ccl2* and *Ccl7* in control unstimulated BMDMs showed no statistically significant differences between WT and MDA5 KO cells [Page 13, Lines 257-258; Appendix Fig. S4A]. We also confirmed that the baseline profile of lung immune cells was highly comparable between WT and MDA5 KO mice (Appendix Fig. S3A, B).

6. A model diagram that integrates the relevant cell types and molecular events underlying the pathogenesis of lung fibrosis would substantially improve clarity and help readers better grasp the proposed central mechanism.

Response: We highly appreciate this valuable suggestion and apologize for the missing diagram. Conforming to the Reviewer's recommendation, we have newly constructed and included a comprehensive central schematic model diagram as Fig. 7G (Graphical Abstract). This diagram successfully integrates the key cell types (damaged cells releasing self-RNA, T cells, neutrophils, and pro-fibrotic SPP1+ / CD11b+ macrophages) and illustrates the molecular switch between normal MDA5-mediated lysosomal RNA clearance versus pathological extracellular RNA accumulation, subsequent TLR8 activation, and the CCR2 ligand-driven fibrotic cascade.

Referee #2 (Comments on Novelty/Model System for Author):

Overall, a good and needed work. Some care should be taken of the results obtained re-using publicly available data.

Referee #2 (Remarks for Author):

With real pleasure and interest, I read the manuscript entitled "The RNA sensor MDA5 protects against lung fibrosis by clearing extracellular self-RNA" (manuscript ID: EMM-2025-22609), written by Ishii and colleagues. This nicely written work is based on a solid study performed by renowned researchers. Furthermore, the topic is intriguing and innovative. As a result, I have only a few comments, mostly minor and/or facultative:

We would like to express our sincere gratitude to Referee #2 for the valuable evaluation of our manuscript and the constructive suggestions. We also deeply appreciate the constructive suggestions, which have undoubtedly helped us improve the clarity, rigor, and comprehensiveness of our manuscript. Below, we provide point-by-point responses to your comments.

1. The Authors quite effectively use publicly available datasets. Usually, I am against a massive usage of such data, and I accept only initial, fishing expedition, replication, or data expansion usage if no other option is available. In this very case, the Authors used publicly data at the very beginning of their work, while starting their investigations, and to expand their results by inclusion of data gathered in human cohorts. In addition, the amount of the public data used is rather well counterbalanced by their Authors' own data, which make a huge majority of the manuscript.
Therefore, I am fine with application of publicly available datasets in this work. Still, I would recommend that the Authors widely discuss usage of publicly available data as a limitation of the study in the respective part of the discussion section.

Response: We sincerely appreciate your positive feedback regarding our approach to integrating publicly available datasets. We fully agree that the

reliance on such datasets carries inherent limitations, including the lack of control over sample processing, potential batch effects, and the unavailability of complete clinical metadata for all cohorts. As recommended, we have now added a paragraph in the limitation section of the Discussion addressing the limitations associated with the re-analysis of publicly available data [Page 28, Lines 584-588].

2. Please, make it clear also in the abstract which parts of the data derive from your won experiments, and which were obtained by re-usage of publicly available datasets.

Response: Thank you for pointing this out. We have revised the Abstract to distinguish between our original experimental data (e.g., *in vitro* mechanistic assays, mouse models of bleomycin-induced lung fibrosis) and the data derived from the re-analysis of publicly available human transcriptomic databases (single-cell RNA-seq and microarray datasets of patients with ILDs/IPF).

3. Results obtained by re-analysis of publicly available datasets should be accompanied by clear description of biomaterials used (cells, tissues, etc.). In case of human data, characteristics of the patients should be given in the supplement.

Response: We agree with this important suggestion. In the revised manuscript, we have ensured that the specific biomaterials used in each public dataset (e.g., whole lung single-cell suspensions, BALF cells, lung homogenates, or skin tissues) are clearly stated in both the Results section and the Figure Legends. Furthermore, we have newly created and added Supplementary Table S1, which summarizes the clinical characteristics and demographics of the human subjects from the utilized public cohorts (GSE135893, GSE70866, GSE47460, etc.), to the extent that such metadata was available in the original databases.

4. Another limiting factor is lack of Authors' own data obtained in primary human cells and/or tissues, especially using modern in vitro/ex vivo such as organoids, air-liquid interface and/or others (PMID: 37626876, 37770004). Inclusion of

such data would substantially further increase the value of this work. If such data are not available, please, at least discuss as a limitation of the study.

Response: We completely agree with the Reviewer. While we successfully validated key molecular mechanisms using the human U937 monocyte cell line and primary mouse bone marrow-derived macrophages (BMDMs), we acknowledge that the lack of primary human cell experiments or advanced 3D *ex vivo* models (such as human lung organoids or precision-cut lung slices) is a limitation of this study. Validating our findings in such advanced human primary models will be an essential next step to bridge the gap toward clinical translation. Following your suggestion, we have discussed this limitation in the revised Discussion section and cited the relevant recent advances in such modeling technologies [Page 28, Lines 588-592].

5. NETs. Please, expand on the role of NETs in physiology and pathology (PMID: 32663035, 36257987). Please, mention some other disorders in which NETosis plays a crucial role (PMID: 39512344, 38928183, etc.).

Response: Thank you for this insightful suggestion and for providing the highly relevant literature. We have expanded our Discussion regarding the role of neutrophil extracellular traps (NETs). Specifically, we have added text detailing that while NETs are physiologically crucial for antimicrobial defense, dysregulated NETosis is deeply involved in the pathology of various disorders, including autoimmune diseases, systemic inflammation, and thrombosis. We have incorporated the suggested concepts to provide a more comprehensive context for extracellular nucleic acid-driven pathologies [Page 27, Lines 561-575].

6. Please, consider further uniformization and unification of quantitative data presentation between the figures.

Response: We appreciate your careful review of our figures. We have thoroughly reviewed all the figures and supplementary figures to ensure the uniformization of quantitative data presentation. Specifically, we have unified the format of our bar/scatter dot plots (e.g., standardizing the display of mean ±

SEM, statistical significance indicators, axis label phrasing, and font sizes across all panels).

7. I am sorry if I overlooked something, but I was not able to see a graphical abstract. I would recommend adding it.

Response: We apologize for the unexpected omission in the initial submission. We have now added a comprehensive Graphical Abstract that clearly illustrates our proposed central mechanism—integrating cellular damage, extracellular RNA release, MDA5-mediated lysosomal clearance vs. TLR8 activation, and subsequent CCR2 ligand-driven fibrosis. This diagram has been added as Fig. 7G.

Referee #3 (Remarks for Author):

General Comments:

Ishii et al. present an original manuscript looking at the RNA sensor MDA5 in protecting against lung fibrosis by clearing extracellular self-RNA. This study looks at MDA5, a cytosolic RNA sensor, in lung fibrosis through use of transgenic MDA5 KO mice and in vitro cell culture systems. After bleomycin, KO mice had exacerbated fibrosis with increased IL-17A gd T cell and neutrophil infiltration, suggesting MDA5 has a protective role independent of MAVS pathway. Mechanistically, authors suggest MDA5 contributes to clearance of extracellular RNA, thereby suppressing macrophage CCR2 ligand expression in a TLR8-dependent manner. Lung macrophages from MDA5 KO mice (resemble SPP1+ mac in humans), show elevated CCR2 ligands.

The KO phenotype is interesting and convincing, but the mechanisms proposed are vague and not as convincing because of the global nature of the knockout, lack of cell specific KO mice, and lack of controls showing the impact of MDA5 KO without bleomycin. Also, the link to ILD is confusing, because MDA5+ antibodies are circulating, not MDA5 itself. It is not clear what MDA5 itself does in myositis ILD, nor is it clear whether antibodies are present in bleomycin induced lung fibrosis. The authors do not look at MDA5 antibodies in any of their studies. Overall, while the study is interesting, there are some major limitations that should be addressed.

Response

We sincerely thank Referee #3 for the comprehensive, thoughtful, and highly constructive evaluation of our manuscript. We appreciate that the Reviewer found our knockout (KO) phenotype interesting and convincing. We have carefully addressed each of your concerns by highlighting key experiments embedded in our revision (such as the macrophage adoptive transfer, Brefeldin A secretion blocking, fluorescent tissue RNA uptake assays with lysosomal inhibition, and baseline homeostatic controls). Below are our point-by-point responses.

Major Comments:

1. The global nature of MDA5 KO makes it difficult to tease apart the specific cellular contributions to the exacerbated phenotype. The authors should consider a more cell-specific targeting strategy to determine the cells involved in MDA5

expression.

Response: We completely agree that relying solely on a global knockout model leaves questions regarding cell-specific contributions. To directly address this limitation without a conditional knockout line, we performed an *in vivo* adoptive transfer experiment (Fig. 5A–D). We isolated BMDMs from either WT or MDA5 KO mice, stimulated them with bleomycin *in vitro*, and intratracheally transferred them into recipient WT mice. Remarkably, the recipient WT mice that received MDA5 KO macrophages exhibited significantly exacerbated lung inflammation and fibrosis compared to those receiving WT macrophages, as demonstrated by micro-CT, histopathology, hydroxyproline assay, and qPCR. This definitive cellular transplantation assay functionally demonstrates that MDA5 deficiency specifically within the myeloid/macrophage compartment is sufficient to drive the exacerbated fibrotic phenotype. We have described this cell-specific evidence in the revised Results [Page 14, Lines 276-284] and Discussion sections [Page 23, Lines 477-482].

2. There is not a clear mechanism which explains specifically how MDA5 would prevent lung fibrosis through regulation of RNA degradation. There is no evidence showing clearance, many mechanisms regulate extracellular deposition of RNA, could also just be due to lack of binding, shifting of binding, re-localization, or increased cell death. It is also not clear how MDA5 gets to the cell membrane of what the significance of this is in the development of disease. Additionally, MDA5 itself has no RNase activity.

Response: We thank the Reviewer for these critical comments, which allow us to clarify the precise molecular and cellular mechanisms underlying our findings:

- **Role as a Scavenger-like Receptor:** We explicitly acknowledge that MDA5 lacks intrinsic RNase catalytic activity. Instead, our data demonstrate that cell-surface MDA5 functions as a scavenger-like shuttle receptor that physically binds extracellular self-RNA and internalizes it into the cell (Fig. 6E, 6G).
- **Evidence of Lysosomal Clearance:** To definitively prove active clearance and subsequent degradation, we performed a quantitative *in vitro* fluorescent RNA (fRNA) clearance assay. By treating cells with RNase immediately prior to harvest, we successfully differentiated and

quantified intracellular versus cell-surface fRNA. Crucially, when we applied the lysosomal inhibitor Chloroquine (CQ), the intracellular clearance of internalized RNA was significantly blocked, resulting in a marked intracellular accumulation of fRNA (Fig. 6H–J). This directly demonstrates that MDA5 transports captured extracellular RNA into the lysosomal pathway for degradation by intrinsic lysosomal RNases. In the absence of MDA5, this RNA escapes degradation, accumulates, and aberrantly hyperactivates the endosomal TLR8 pathway.

- **Mechanism of Surface Translocation:** To determine how cytoplasmic MDA5 reaches the plasma membrane, we performed a Brefeldin A (BFA) experiment (Fig. 1G). BFA, an inhibitor of ER-to-Golgi vesicle transport, completely abolished the bleomycin-induced cell-surface translocation of MDA5. This indicates that cellular stress actively triggers MDA5 translocation via the classical exocytic secretory pathway, anchoring it to the membrane to act as a frontline scavenger.

3. KO of MDA5 causes apoptosis and increased inflammation, which may have nothing to do specifically with fibrosis or RNA clearance. This is not considered in the manuscript.

Response: Thank you for raising this critical pathophysiological question. We thoroughly characterized the nature of cell death in our model. In Fig.6A, we showed that MDA5 suppression significantly increases susceptibility to necrotic cell death (Annexin-V^-/PI^+), but not apoptosis, at 72 hours post-bleomycin. To disentangle whether the pro-fibrotic inflammation is merely a non-specific byproduct of cell death, we performed a crucial rescue experiment using exogenous RNase treatment. Remarkably, while RNase treatment did not alter the rates of bleomycin-induced necrotic cell death (Appendix Fig. S4C, D), it completely rescued and suppressed the hyper-production of CCR2 chemokines (CCL2/8) in MDA5-suppressed cells (Fig. 6B). This uncoupling proves that the hyper-inflammatory cascade is driven specifically by the aberrant accumulation of extracellular self-RNA DAMPs due to a clearance defect, rather than being a non-specific consequence of cellular necrosis.

4. In Figure 1B, only levels of MDA5 in BAL are shown pre/post bleomycin, but not in BAL from MDA5 KO mice both with and without bleomycin, the mice should

lose the effect if specific to MDA5. This is an important control.

Response: We sincerely thank the Reviewer for this highly constructive and important comment. To address this point, we have revised Fig. 1B by utilizing the average measurement values from the BALF of MDA5 KO mice (both under control and bleomycin-treated conditions) as background levels and subtracting them from the corresponding values of WT mice. Reassuringly, even after subtracting these background levels, the overall experimental conclusion remained unchanged, demonstrating a distinct and significant decrease in BALF MDA5 levels upon bleomycin treatment.

Furthermore, regarding the serum MDA5 data (previously presented in Appendix Fig. S1B), since the absolute levels were originally low, needed further investigation, and held minimal relevance to the central theme of our study (i.e., local extracellular RNA clearance pathways in the lung), we have decided to remove the serum dataset from the revised manuscript to maintain a clear and streamlined focus.

We have updated the corresponding description in the Figure Legends (Fig. 1B) and the Results section accordingly.

5. In Figure 1E, changes in fluorescence are not equivalent to RNA clearance, and are not specific to degradation and are also not quantitative.

Response: We thank the Reviewer for this valid critique regarding the descriptive nature of static immunofluorescence imaging in tissue sections. We agree that changes in tissue fluorescence alone do not definitively demonstrate active clearance or specific degradation, nor are they strictly quantitative.

Accordingly, we have made the following revisions to address this concern:

1. **Terminology Revision:** We have replaced the term "RNA clearance" with "extracellular RNA accumulation" in the corresponding parts of the text to more accurately reflect the descriptive nature of the *in situ* staining.
2. **Clarification of Histological Data:** Regarding the quantification of *in vivo* tissue fluorescence, precise *in situ* quantification is technically challenging due to the structural complexity and high spatial heterogeneity of fibrotic lung lesions. To provide a clearer qualitative assessment, we have provided representative images of the lung sections at both low and high magnifications in the main figure (Fig. 6F).

3. **Integration with Quantitative Evidence:** To supply the rigorous, quantitative, and specific evidence of RNA turnover requested, we designed and executed the *in vitro* fluorescent RNA (fRNA) clearance assay (Fig. 6G–J). By utilizing flow cytometry to measure mean fluorescence intensity (MFI) immediately prior to harvest, we successfully quantified internalization kinetics. When combined with the Chloroquine-mediated lysosomal blockade (which halted fluorescence decay), this platform provides definitive, quantitative evidence of lysosome-dependent RNA degradation.

We believe that the qualitative *in situ* tissue localization, when supported by these rigorous quantitative *in vitro* kinetics, collectively validates our conclusions.

6. In Figure 1E, lung tissue is shown after bleomycin showing RNA in the cytoplasm and extracellular spaces, without histology staining it is not clear whether these are fibrotic areas. Adjacent sections of HE or trichrome should be shown for context. Also, authors should show this experiment in KO mice not treated with bleomycin as an important control.

Response: We thank the Reviewer for this highly constructive suggestion, which has significantly improved the quality and clarity of our histological data.
As suggested, we performed the same RNA staining experiments using MDA5 KO mice. As expected, the MDA5 KO mice displayed more prominent RNA staining that was successfully diminished by RNase treatment. Therefore, we have relocated the WT mouse data to Appendix Figure S4H (right panel) and presented the comprehensive MDA5 KO mouse data in the revised Fig. 6F.
To provide a definitive histological context, we have now added representative Masson's trichrome-stained images of adjacent sections in the right panels of Fig. 6F. These adjacent sections clearly demonstrate that the areas with intense RNA accumulation morphologically correspond to dense fibrotic and inflammatory foci. Furthermore, to address the request for an important control, we have included both untreated (saline) and bleomycin-treated MDA5 KO mouse lung sections in Fig. 6F (left and middle/right panels, respectively). This comparison confirms that there is no aberrant extracellular RNA accumulation under baseline (untreated) conditions.

7. In Figure 3 (and throughout the paper), there is a lack of important controls

showing that the immune phenotype is not induced just by MDA5 KO, independent of bleomycin. This would be clearer if they looked at MDA5 KO with and without bleomycin. This holds true both in mice and in vitro KO as well.

Response: We thank the Reviewer for this important comment regarding baseline homeostasis. We have added these crucial control data in the manuscript.

Specifically, in Appendix Fig. S3, we characterized the baseline, steady-state immune profiles of untreated (saline-control) WT, MDA5 KO, MAVS KO, and IFNAR KO mice. Our comprehensive flow cytometric quantification demonstrated that under steady-state baseline conditions, there are no significant differences in the total proportions or specific subsets of lung lymphocytes (including IL-17A$^+$ γδ T cells), macrophages, or neutrophils across all genotypes.

Similarly, *in vitro* BMDMs showed comparable baseline expression profiles of CCR2 ligands without stimulation (Appendix Fig. S4A). Furthermore, baseline CT images and Masson's trichrome-stained tissue images of control MDA5 KO mice are also included in Fig. 2G.

These internal controls definitively confirm that the hyper-inflammatory immune phenotype is not a developmental or spontaneous artifact of MDA5 deficiency, but is specifically triggered by tissue injury.

.

8. Use of BMDMs from mice may not translate into lung specific macrophage findings. In addition, direct exposure to bleomycin may not represent ILD or even bleomycin in vivo. RNAseq data therefore may not relate to lungs. Human validation with monocyte cell line helps, but again bleomycin used rather than profibrotic cytokines.

Response: We understand the Reviewer's concerns regarding the translational fidelity of bone marrow-derived cells and *in vitro* drug exposure. We have addressed these points through a multi-tiered validation approach:

- **Primary Lung Macrophages:** To ensure our findings translate to lung-specific contexts, we validated our core mechanism using **primary lung alveolar macrophages**. We confirmed that bleomycin induces a comparable hyper-inflammatory TNF-α response in MDA5 KO primary lung cells (Appendix Fig. S2F), demonstrating that the phenotype is not

restricted to bone marrow-derived cells.

- **Stimulus Specificity:** To address why we utilized cell-injury models rather than cytokine stimulation, we treated cells with the profibrotic cytokine PDGF-BB (Fig. 4G). Crucially, PDGF-BB did not elicit differential chemokine production between WT and KO cells. This specificity underscores that MDA5's protective mechanism is uniquely designed to sense and clear damage-associated molecular patterns (DAMPs) arising from tissue injury, rather than serving as a universal cytokine repressor.

These results indicate that reagents triggering active tissue or cellular injury are far more physiologically relevant than pure cytokines for analyzing this specific MDA5-dependent pathway. Nevertheless, we agree that caution is required when directly extrapolating *in vitro* and animal data to human clinical settings, even though our analysis of human ILD datasets (Fig. 7) remains highly consistent with our model. We have explicitly detailed this translational limitation in the revised Discussion [Page 28, Lines 593-597].

9. There is increased surface distribution of MDA5 with bleomycin, but it is not explained why or how this occurs.

Response: We agree that the translocation mechanism requires deeper elucidation. As detailed in our response to Major Comment 2, we performed a mechanistic Brefeldin A (BFA) vesicle-blocking experiment (Fig. 1G). The complete blockade of surface translocation by BFA demonstrates that cellular stress and damage signals induced by bleomycin actively mobilize cytosolic MDA5 into the classical ER-to-Golgi exocytosis pathway, anchoring it to the plasma membrane.

10. It may be that increased RNA is released after bleomycin in MDA5 KO mice and cells, but this is not quantified, and it could be due to increased cell apoptosis releasing RNA, this is not considered, and cell death itself is a relatively non-specific mechanism.

We thank the Reviewer for this insightful comment. We addressed this point by carefully considering and quantifying the relationship between cell death and RNA accumulation.

Regarding apoptosis, the proportion of Annexin V^+/PI^- apoptotic cells showed no significant change between the control- and *IFIH1* siRNA-transfected U937 cells upon bleomycin treatment, and the apoptotic fraction itself was relatively small compared to the necrotic fraction. Therefore, we focused on necrotic cells for the subsequent evaluations.

To definitively rule out cell death as a confounding factor, in Appendix Fig. S4F, we utilized flow cytometry to separate live (PI^-) and necrotic (PI^+) populations and quantified their RNA fluorescence. While necrotic cells expectedly exhibited high RNA signals due to membrane permeabilization, the *IFIH1*-suppressed cells exhibited significantly higher RNA accumulation even within the live (PI^-) fraction compared to their control counterparts. This crucial finding demonstrates that the defect in RNA clearance represents an active, receptor-mediated failure present in intact, viable cells, rather than a passive mathematical artifact reflecting an increased number of dead cells.

11. The authors link MDA5 to ILD, but the link is not clear, there are no measurements of MDA5 antibodies in the models, or levels of MDA5 in circulation from ILD patients.

Response: We thank the Reviewer for this important comment. We agree that the clinical framing required clarification, and we have revised the text accordingly. First, to clarify the link to broader clinical contexts, Fig. 7 focuses on progressive ILD in general, demonstrating that the MDA5–macrophage axis is relevant to a wide spectrum of ILD pathogenesis rather than being restricted to a specific subtype.

Second, as the Reviewer correctly noted, we did not measure MDA5 autoantibodies or circulating MDA5 levels in this study. Acknowledging this limitation, we have carefully adjusted our discussion regarding anti-MDA5 autoantibody-associated ILD. Given our novel findings that MDA5 translocates to the cell surface to regulate extracellular RNA, it is highly conceivable that these surface-exposed MDA5 molecules could be directly recognized by circulating autoantibodies, thereby pathologically disrupting extracellular RNA dynamics and homeostasis.

While this represents a compelling molecular model for autoantibody-mediated pathogenesis, we agree that its experimental validation remains an important open question. We have clarified these points in the revised Discussion and

positioned the measurement of circulating clinical markers and the functional analysis of autoantibodies as a crucial future direction.

Minor Comments:

1. In Figure 1C, the MDA5 KO mouse histology does not look worse than WT, although OHP shows it is more fibrotic in Figure 2. It would be good to use representative images that match other figures quantification.

Response: We thank the Reviewer for pointing this out. We understand that the appearance of Fig. 1C may cause confusion regarding the severity of fibrosis.
We would like to clarify that Fig. 1C focuses strictly on the immunohistochemical (IHC) localization and verification of MDA5 protein expression. Because heavily fibrotic tissues are highly prone to non-specific background staining—especially in the expanded interstitial matrix—we prioritized selecting regions with clearer tissue architecture to guarantee accurate IHC interpretation.
To avoid any misunderstanding for readers, we have added a clarifying statement in the revised figure legend to explain this selection criteria. As noted, the quantitative and definitive evaluation of fibrosis severity is comprehensively provided via micro-CT, Masson's trichrome, and hydroxyproline assays in Fig. 2.
.

2. In Figure 2A, authors report CT scan shows worse disease in MDA5 KO, but invasive shadows and GGOs are very non-specific, and could represent edema, inflammation or fibrosis. This should be explained.

Response: We completely agree with the Reviewer's clinical assessment. GGOs and consolidations on micro-CT are indeed non-specific and reflect a combined landscape of active alveolar inflammation, proteinaceous edema, and progressive structural fibrosis. We have added a sentence in the Results section to explicitly clarify this interpretation [Page 9, Lines 156-158].

3. The analysis of scRNAseq is vague, "some structural cells", "patients with ILDs" there are so many questions here like what cells are being compared and what ILDs are being compared.

Response: We sincerely apologize for the lack of granularity and the vague

phrasing in our initial submission. We have revised the text in the Methods, Results, and Figure Legends to provide precise definitions of both the clinical cohorts and the specific cell types being compared.

- **Clarification of "patients with ILDs":** Our re-analysis of the single-cell RNA-sequencing dataset (GSE135893) involved a precise comparison between non-fibrotic controls (declined organ donors; n = 10) and patients with progressive fibrotic Interstitial Lung Diseases (ILDs; n = 20). We have now explicitly listed the exact clinical etiologies of these ILD patients in the revised manuscript: Idiopathic Pulmonary Fibrosis (IPF; n = 12), chronic hypersensitivity pneumonitis (cHP; n = 3), nonspecific interstitial pneumonia (NSIP; n = 2), sarcoidosis (n = 2), and unclassifiable ILD (n = 1), exactly as categorized in the landmark original study.
- **Clarification of "some structural cells" and compared cell types:** We have corrected the expression "some structural cells" and replaced it with precise cell-type designations based on our statistical analysis. While we comprehensively screened all major structural and immune cell clusters (illustrated in Appendix Fig. S1A), statistically significant upregulation of *IFIH1* expression was identified in endothelial cells (ECs) within the structural compartment, and NK cells within the immune compartment.

26th Jun 2026

Dear Dr. Ishii,

Thank you for submitting your revised manuscript, which has now been evaluated by the initial referees. As you will see below, they are overall satisfied with the revisions, and I will therefore be able to accept your manuscript once the following points have been addressed:

1/ Please address the remaining comments raised by referee #1 through additional clarification and discussion. The RNA imaging experiments should be quantified as requested. Experiments with an endocytosis inhibitor are welcome, but not mandatory for further consideration.

2/ Manuscript text:
- Please remove the red font text and only keep in track changes mode any new modification.
- The institutional email address of the corresponding author should be entered in our submission system.
- Materials and methods should be Methods. Additionally, please provide the following information:
o Animal experiments: Include a statement of compliance with ethical regulations. Provide reference number for ethic approval.
o Antibodies: Provide dilutions/concentrations.
o Cell lines: Indicate whether the cells were tested for mycoplasma contamination, and state if they were authenticated.
o Statistics: provide statements about sample size, blinding, randomization, and inclusion/exclusion criteria.
- Data Availability: Remove "All other data are available within the manuscript and its supplemental materials." Please note that deposited datasets must be publicly accessible before acceptance of the manuscript.
- Please rename "Disclosure" to "Disclosure and competing interests statement".
- Our journal encourages inclusion of *data citations in the reference list* to directly cite datasets that were re-used and obtained from public databases. Data citations in the article text are distinct from normal bibliographical citations and should directly link to the database records from which the data can be accessed. In the main text, data citations are formatted as follows: "Data ref: Smith et al, 2001" or "Data ref: NCBI Sequence Read Archive PRJNA342805, 2017". In the Reference list, data citations must be labeled with "[DATASET]". A data reference must provide the database name, accession number/identifiers and a resolvable link to the landing page from which the data can be accessed at the end of the reference.

3/ Figures:
- Figure 7 spans over 2 pages, which is not allowed. Please rearrange to have either all panels on page, or separate in Figure 7 and Figure 8.
- We would encourage you to make your Appendix figures into Expanded View (EV) Figures and Tables that are collapsible/expandable online. EV Figures should be cited as 'Figure EV1, Figure EV2" etc... in the text and their respective legends should be included in the main text after the legends of regular figures.
- For the figures that you do NOT wish to display as Expanded View figures, they should be bundled together with their legends in a single PDF file called *Appendix*, which should start with a short Table of Content and have page numbers. Appendix figures should be referred to in the main text as: "Appendix Figure S1, Appendix Figure S2" etc.
- Please correct the callouts in the manuscript text file accordingly.
- Figure 2B, 6F, Appendix Fig S2B, Appendix Fig S4H, please add arrows or highlight boxes to the smallest magnification.
- During our standard image check, a possible reuse of graph was found between Appendix Fig S1A RD and S5A 1F1H1, please clarify. Please note that figure re-use is allowed, but should be indicated in the figure legend.
- Please address the queries from our data editors in the figure legends:
1. Please define the annotated p values **/*** as well as provide the exact p-values for the same in the legend of figure 1G; as appropriate.
2. Please note that the exact p values are not provided in the legends of 2C,D,E,F,H,I; 3B,D,E; 4D,E,F,G; 5B,D,F,H,I; 6A,B,D,G,H,J; 7D-F; Appendix figure S1-B; Appendix figure S2-C-F,I; Appendix figure S3-C; Appendix figure S4-B-G; Appendix figure S5-B.
3. Please note that the box plots need to be defined in terms of minima, maxima, centre, bounds of box and whiskers, and percentile in the legends of figures 7D,F.
4. Please note that information related to n is missing in the legends 1G; 7D.
5. Please note that the error bars are not defined in the legends of figure 1G.
6. Please note that the white arrowheads are not defined in the legend of figure 6F. This needs to be rectified.

4/ Checklist:
- in the top left corner, please indicate the name of the corresponding author (not Lise Roth)
- cell materials: fill in the subsection on authentication and mycoplasma contamination
- experimental animals: check 'animal observed in or captured from the field', as I don't think it applies to your study.

5/ Source Data:
- Please provide raw FACS data
- The folders for main figures need to be uploaded separately, while the ones for the Appendix figures can stay combined.

6/ Thank you for providing The paper explained. Please provide a bit more details, in particular for the section 'Results'. Please refer to any of our recently published articles for reference.

7/ Every published paper includes a 'Synopsis' to further enhance discoverability. Synopses are displayed on the journal webpage and are freely accessible to all readers. They include a short stand first (maximum of 300 characters, including space) as well as 2-5 one-sentences bullet points that summarizes the paper. Please write the bullet points to summarize the key NEW findings. They should be designed to be complementary to the abstract - i.e. not repeat the same text. We encourage inclusion of key acronyms and quantitative information (maximum of 30 words / bullet point). Please use the passive voice. Please attach these in a separate file or send them by email, we will incorporate them accordingly.

Please also suggest a striking image or visual abstract to illustrate your article as a PNG file 550 px wide x 300 px high.

8/ As part of the EMBO Publications transparent editorial process initiative (see our Editorial at http://embomolmed.embopress.org/content/2/9/329), EMBO Molecular Medicine will publish online a Review Process File (RPF) to accompany accepted manuscripts.
This file will be published in conjunction with your paper and will include the anonymous referee reports, your point-by-point response and all pertinent correspondence relating to the manuscript. Let us know whether you agree with the publication of the RPF.
Please note that the Authors checklist will be published at the end of the RPF.

I look forward to receiving your revised manuscript.

Yours sincerely,

Lise Roth

Lise Roth, PhD
Senior Editor
EMBO Molecular Medicine

***** Reviewer's comments *****

Referee #1 (Comments on Novelty/Model System for Author):

Additional quantification of the imaging data is required

Referee #1 (Remarks for Author):

The authors have made a substantial effort to address the reviewers' concerns and have strengthened the manuscript. In particular, the additional biochemical and imaging experiments demonstrating membrane-associated MDA5 and its interaction with extracellular RNA, as well as the inclusion of IFNAR KO mice to examine the contribution of type I IFN signalling in the bleomycin-induced ILD model, have increased confidence in the core conclusions. These revisions are commendable. However, I believe several points/data still require additional clarification and quantification, or a more balanced discussion of the limitations before the manuscript is ready for publication.

- In my previous review, I asked the authors to reconcile the seemingly counterintuitive observation that MDA5 levels decrease in BALF while intracellular and membrane-associated MDA5 increase following bleomycin treatment. The explanation provided in the rebuttal is reasonable, but it is important that this interpretation also be included in the manuscript itself rather than remaining solely in the response letter.

- I would have been particularly interested to see a comparison between MDA5 KO and MDA5-IFNAR double KO mice with respect to chemokine production and fibrotic signalling. Such an experiment would help clarify the specific contribution of type I IFN signalling and determine whether additional innate sensing pathways are involved. The current comparison between MDA5 KO and IFNAR KO mice provides only limited mechanistic insight. While I do not consider this experiment essential for publication given the novelty of the extracellular MDA5 findings, this limitation should be clearly acknowledged in the Discussion and highlighted as an important direction for future work.

- The authors should revisit several statements to ensure they accurately reflect the data. For example, lines 195-196 state that IFNAR KO mice have notably fewer macrophages than MDA5 KO mice (Fig S2H), but this does not appear to be supported by the data shown.

- Lines 264-267: The data presented do not appear to have been generated using "primary lung macrophages" as stated. This should be clarified or corrected.

- Lines 268-270: Without data from MDA5-IFNAR double KO cells, I do not think the conclusion presented here is sufficiently supported. The sentence should be revised.

- Lines 304-305: The reference cited here does not justify the use of DNase, which I believe was the authors' intention. MDA5 remains an RNA sensor during "DNA virus infection". A more appropriate rationale should be provided.

- The RNA imaging experiments in Fig 6 absolutely require additional quantification analyses. When comparing Fig 6C and Fig 6E, the interpretation is not entirely clear. Based on the proposed model, one would expect bleomycin-treated MDA5 KO cells to contain more extracellular RNA. This is the case in 6C but not apparent in 6E. In addition, more representative images showing punctate colocalization between MDA5 and RNA in WT cells should be included along with appropriate quantification.

- The authors should also provide a clearer explanation of when extracellular RNA would be expected to be susceptible or resistant to exogenous RNase treatment (Fig 6C, Fig 6G, and Fig S4F). Based on the current model, I would have expected MDA5 deficiency to reduce RNA internalization, thereby rendering extracellular RNA more susceptible to RNase digestion. However, this does not seem to be the case in Fig 6G.

- The authors may wish to consider using an endocytosis inhibitor, such as Pitstop 2, to directly test whether MDA5-mediated RNA uptake is more broadly required for downstream signalling. The TLR8 aspect of the study remains largely correlative and currently relies heavily on extrapolation from previous work in neutrophils.

Referee #2 (Comments on Novelty/Model System for Author):

As addressed in my original review report

Referee #2 (Remarks for Author):

Thank you very much for addressing my comments. I have no further reservations.

Referee #3 (Remarks for Author):

The authors have adequately addressed my concerns.

Referee #1 (Remarks for Author):

The authors have made a substantial effort to address the reviewers' concerns and have strengthened the manuscript. In particular, the additional biochemical and imaging experiments demonstrating membrane-associated MDA5 and its interaction with extracellular RNA, as well as the inclusion of IFNAR KO mice to examine the contribution of type I IFN signalling in the bleomycin-induced ILD model, have increased confidence in the core conclusions. These revisions are commendable. However, I believe several points/data still require additional clarification and quantification, or a more balanced discussion of the limitations before the manuscript is ready for publication.

We sincerely thank the reviewer for their positive evaluation of our revised manuscript and for their constructive feedback. We have carefully addressed each of the remaining points by revising the text, adding the requested quantifications, and ensuring a more balanced discussion of our study's limitations. Please find our point-by-point responses below.

- In my previous review, I asked the authors to reconcile the seemingly counterintuitive observation that MDA5 levels decrease in BALF while intracellular and membrane-associated MDA5 increase following bleomycin treatment. The explanation provided in the rebuttal is reasonable, but it is important that this interpretation also be included in the manuscript itself rather than remaining solely in the response letter.

Response: We apologize for forgetting to add the content for the Discussion section. We have now incorporated the explanation provided in our previous rebuttal directly into the Discussion section to explicitly reconcile this observation. We added the relevant explanation on Page 21-22, Lines 523-537.

- I would have been particularly interested to see a comparison between MDA5 KO and MDA5-IFNAR double KO mice with respect to chemokine production and fibrotic signalling. Such an experiment would help clarify the specific contribution of type I IFN signalling and determine whether additional innate sensing pathways are involved. The current comparison between MDA5 KO and IFNAR KO mice provides only limited mechanistic insight. While I do not

consider this experiment essential for publication given the novelty of the extracellular MDA5 findings, this limitation should be clearly acknowledged in the Discussion and highlighted as an important direction for future work.

Response: We appreciate this insightful comment and agree that evaluating MDA5-IFNAR double KO mice would provide a more definitive understanding of the specific contributions of type I IFN signaling versus other innate sensing pathways. As suggested, we have now clearly acknowledged this limitation in our manuscript and highlighted it as a critical direction for future research. A discussion regarding this limitation has been added to the Discussion section (Page 29, Lines 699-707).

- The authors should revisit several statements to ensure they accurately reflect the data. For example, lines 195-196 state that IFNAR KO mice have notably fewer macrophages than MDA5 KO mice (Fig S2H), but this does not appear to be supported by the data shown.

Response: We apologize for the errors and sincerely thank the reviewer for pointing them out. We realized that the figure citation was incorrect; the data regarding lung macrophage populations are shown in Figure EV2I (Appendix Figure S2I), rather than Appendix Figure S2H (which displays BALF cells). To accurately reflect the actual data, we have revised the statement by removing the word "notably" and toning down the description. The text on Lines 215-216 has been corrected.

- Lines 264-267: The data presented do not appear to have been generated using "primary lung macrophages" as stated. This should be clarified or corrected.

Response: We thank the reviewer for catching this error. We have corrected "primary lung macrophages" to "primary alveolar macrophages" (Line 300).

- Lines 268-270: Without data from MDA5-IFNAR double KO cells, I do not think the conclusion presented here is sufficiently supported. The sentence should be revised.

Response: We completely agree with the reviewer that stating the chemokine response is "independent of" type I IFN signaling was an overstatement without direct data from MDA5-IFNAR double KO cells.
Following your valuable suggestion, we have toned down the conclusion to more accurately reflect our findings. We have revised the sentence to state that the enhanced chemokine production in MDA5-deficient cells cannot be fully explained solely by the reduction or attenuation of classical type I IFN signaling (Line 303-313).

- Lines 304-305: The reference cited here does not justify the use of DNase, which I believe was the authors' intention. MDA5 remains an RNA sensor during "DNA virus infection". A more appropriate rationale should be provided.

Response: We completely agree with the reviewer's excellent point. Our previous text and citation were misleading, as MDA5 indeed functions as an RNA sensor even during DNA virus infection.
Our actual intention in using DNase was not related to DNA virus sensing, but rather to account for the fact that dying or damaged cells release a mixture of both RNA and DNA into the extracellular space. Therefore, we used DNase to evaluate or rule out any indirect contributions or confounding effects of extracellular DNA on the inflammatory responses.
We have removed the inappropriate reference and revised the text to provide this clear and accurate rationale (Line 352-357).

- The RNA imaging experiments in Fig 6 absolutely require additional quantification analyses. When comparing Fig 6C and Fig 6E, the interpretation is not entirely clear. Based on the proposed model, one would expect bleomycin-treated MDA5 KO cells to contain more extracellular RNA. This is the case in 6C but not apparent in 6E. In addition, more representative images showing punctate colocalization between MDA5 and RNA in WT cells should be included along with appropriate quantification.

Response: In accordance with the reviewer's suggestion, we have now performed robust quantification of the RNA imaging experiments. The RNA signals in Figure 6C, 6E, and 6F were quantified using Nikon AX/AXR analysis

software, and the resulting statistical graphs have been added adjacent to their respective panels in the revised Figure 6.

1. Regarding the interpretation of Fig 6C and Fig 6E: Regarding the visual discrepancy pointed out between the single representative images in Fig 6C and 6E, a closer inspection of the magnified view in Fig 6E reveals that the RNA signals in bleomycin-treated MDA5 KO cells are highly robust and fully comparable to those in WT cells, especially when considering the respective cell sizes. Furthermore, as shown in the newly added quantification graphs for Fig 6C and 6E, the objective statistical analysis across multiple fields of view clearly demonstrates a significant increase in extracellular RNA in bleomycin-treated MDA5 KO cells compared to WT cells, which supports our proposed model. The subtle initial impression is primarily due to the different experimental focuses and imaging scales: Fig 6C provides a global view of the extracellular environment at a lower magnification, whereas Fig 6E focuses on individual cells at a higher magnification with a specific focal plane optimized to examine intracellular/pericellular localization.

2. Regarding the punctate colocalization and representative images**:** To address the concern regarding the colocalization of MDA5 and RNA in WT cells, we believe that relying on objective statistical parameters provides a more rigorous verification than merely presenting selected images. By utilizing the Nikon AX/AXR analysis software, we quantified the spatial correlation (Pearson's correlation coefficient) between the MDA5 and RNA signals, which is now clearly presented in the new graph adjacent to Fig 6E. This quantitative data objectively proves that the punctate colocalization events in WT cells are statistically significant and robust, ensuring a reliable interpretation of our typical and unbiased imaging data.

- The authors should also provide a clearer explanation of when extracellular RNA would be expected to be susceptible or resistant to exogenous RNase treatment (Fig 6C, Fig 6G, and Fig S4F). Based on the current model, I would have expected MDA5 deficiency to reduce RNA internalization, thereby rendering extracellular RNA more susceptible to RNase digestion. However, this does not seem to be the case in Fig 6G.

Response: We sincerely appreciate the reviewer for pointing out this critical aspect, which requires careful mechanistic explanation. We hypothesize that in WT cells, extracellular RNA is efficiently captured and temporarily retained on the cell surface by MDA5, which renders it highly accessible and susceptible to exogenous RNase treatment.

Conversely, in MDA5-deficient cells, the lack of this surface trapping allows the RNA to be taken up via alternative, MDA5-independent uptake pathways. Once this RNA flows into the intracellular space through these alternative routes, it becomes physically protected from exogenous RNase, explaining the apparent resistance observed in Fig 6G. Over time, this uncontrolled influx leads to intracellular RNA accumulation and overwhelms the overall cellular clearance capacity. Ultimately, this cellular-level clearance failure results in the widespread accumulation of extracellular RNA at the tissue level, as observed *in vivo* in MDA5 KO mice.

We have now explicitly incorporated this mechanistic rationale into the Discussion section to clarify the dynamics of RNA internalization, clearance, and RNase susceptibility.

We have added an additional explanation regarding the balance between surface trapping by MDA5 and alternative RNA uptake mechanisms in the Discussion section (Page 26-27, Lines 626-651).

- The authors may wish to consider using an endocytosis inhibitor, such as Pitstop 2, to directly test whether MDA5-mediated RNA uptake is more broadly required for downstream signalling. The TLR8 aspect of the study remains largely correlative and currently relies heavily on extrapolation from previous work in neutrophils.

Response: We completely agree that directly testing RNA uptake with an endocytosis inhibitor like Pitstop 2 would be highly informative, especially given our proposed model that alternative endocytic pathways might mediate RNA influx in the absence of MDA5 (as discussed in Point 8).

We acknowledge that our proposed TLR8 mechanism relies partly on extrapolations from previous literature, and identifying the specific alternative endocytic pathways using inhibitors will be a crucial next step for our future mechanistic studies. We have added a comment in the Discussion section (Page 26-27, Lines 642-651) explicitly acknowledging this limitation and

highlighting the future use of endocytosis inhibitors to dissect these uptake pathways.

19th Aug 2026

Dear Dr. Ishii,

We are pleased to inform you that your manuscript is accepted for publication and is now being sent to our publisher to be included in the next available issue of EMBO Molecular Medicine.

Your manuscript will be processed for publication by EMBO Press. It will be copy edited and you will receive page proofs prior to publication. Please note that you will be contacted by Springer Nature Author Services to complete licensing and payment information.

You may qualify for financial assistance for your publication charges - either via a Springer Nature fully open access agreement or an EMBO initiative. Check your eligibility: https://link.springer.com/journal/44321/how-to-publish-with-us

Should you be planning a Press Release on your article, please notify the production team via this form in order to coordinate publication and release dates: https://forms.cloud.microsoft/e/eH4A8JVP6g

If you have any questions, please do not hesitate to contact the Editorial Office. Thank you for your contribution to EMBO Molecular Medicine.

Yours sincerely,
Lise Roth

Lise Roth, Ph.D
Senior Editor
EMBO Molecular Medicine

>>> Please note that it is EMBO Molecular Medicine policy for the transcript of the editorial process (containing referee reports and your response letter) to be published as an online supplement to each paper. If you do NOT want this, you will need to inform the Editorial Office via email immediately. More information is available here: https://link.springer.com/partners/embo-press/editorial-policies#Peer%20review

EMBO Press Author Checklist

Corresponding Author Name: Dr. Takashi Ishii	
Journal Submitted to: EMBO Molecular Medicine	
Manuscript Number: EMM-2025-22609	

USEFUL LINKS FOR COMPLETING THIS FORM
[The EMBO Journal - Author Guidelines]
[EMBO Reports - Author Guidelines]
[Molecular Systems Biology - Author Guidelines]
[EMBO Molecular Medicine - Author Guidelines]

Reporting Checklist for Life Science Articles (updated January
This checklist is adapted from Materials Design Analysis Reporting (MDAR) Checklist for Authors. MDAR establishes a minimum set of requirements in transparent reporting in the life sciences (see Statement of Task: 10.31222/osf.io/9sm4x). Please follow the journal's guidelines in preparing your
Please note that a copy of this checklist will be published alongside your article.

Abridged guidelines for figures
1. Data
The data shown in figures should satisfy the following conditions:
- the data were obtained and processed according to the field's best practice and are presented to reflect the results of the experiments in an accurate and unbiased manner.
- ideally, figure panels should include only measurements that are directly comparable to each other and obtained with the same assay.
- plots include clearly labeled error bars for independent experiments and sample sizes. Unless justified, error bars should not be shown for technical
- if n<5, the individual data points from each experiment should be plotted. Any statistical test employed should be justified.
- Source Data should be included to report the data underlying figures according to the guidelines set out in the authorship guidelines on Data

2. Captions
Each figure caption should contain the following information, for each panel where they are relevant:
- a specification of the experimental system investigated (eg cell line, species name).
- the assay(s) and method(s) used to carry out the reported observations and measurements.
- an explicit mention of the biological and chemical entity(ies) that are being measured.
- an explicit mention of the biological and chemical entity(ies) that are altered/varied/perturbed in a controlled manner.
- the exact sample size (n) for each experimental group/condition, given as a number, not a range;
- a description of the sample collection allowing the reader to understand whether the samples represent technical or biological replicates (including how many animals, litters, cultures, etc.).
- a statement of how many times the experiment shown was independently replicated in the laboratory.
- definitions of statistical methods and measures:
  - common tests, such as t-test (please specify whether paired vs. unpaired), simple χ2 tests, Wilcoxon and Mann-Whitney tests, can be unambiguously identified by name only, but more complex techniques should be described in the methods section;
  - are tests one-sided or two-sided?
  - are there adjustments for multiple comparisons?
  - exact statistical test results, e.g., P values = x but not P values < x;
  - definition of 'center values' as median or average;
  - definition of error bars as s.d. or s.e.m.

Please complete ALL of the questions below.
Select "Not Applicable" only when the requested information is not relevant for your study.

Materials

Newly Created Materials	Information included in the manuscript?	In which section is the information available? (Reagents and Tools Table, Materials and Methods, Figures, Data Availability Section)
New materials and reagents need to be available; do any restrictions apply?	Yes	Regents and Tools Table, Methods

Antibodies	Information included in the manuscript?	In which section is the information available? (Reagents and Tools Table, Materials and Methods, Figures, Data Availability Section)
For **antibodies** provide the following information:<br>- Commercial antibodies: RRID (if possible) or supplier name, catalogue number and or/clone number<br>- Non-commercial: RRID or citation	Yes	Regents and Tools Table, Methods

DNA and RNA sequences	Information included in the manuscript?	In which section is the information available? (Reagents and Tools Table, Materials and Methods, Figures, Data Availability Section)
**Short novel DNA or RNA including primers, probes**: provide the sequences.	Yes	Regents and Tools Table, Methods

Cell materials	Information included in the manuscript?	In which section is the information available? (Reagents and Tools Table, Materials and Methods, Figures, Data Availability Section)
**Cell lines:** Provide species information, strain. Provide accession number in repository **OR** supplier name, catalog number, clone number, and/**OR** RRID.	Yes	Regents and Tools Table, Methods
**Primary cultures:** Provide species, strain, sex of origin, genetic modification status.	Yes	Methods
Report if the cell lines were recently **authenticated** (e.g., by STR profiling) and tested for mycoplasma contamination.	Yes	Methods

Experimental animals	Information included in the manuscript?	In which section is the information available? (Reagents and Tools Table, Materials and Methods, Figures, Data Availability Section)
**Laboratory animals or Model organisms:** Provide species, strain, sex, age, genetic modification status. Provide accession number in repository **OR** supplier name, catalog number, clone number, **OR** RRID.	Yes	Regents and Tools Table, Methods
**Animal observed in or captured from the field:** Provide species, sex, and age where possible.	Not Applicable	
Please detail **housing and husbandry conditions**.	Yes	Methods

Plants and microbes	Information included in the manuscript?	In which section is the information available? (Reagents and Tools Table, Materials and Methods, Figures, Data Availability Section)
**Plants:** provide species and strain, ecotype and cultivar where relevant, unique accession number if available, and source (including location for collected wild specimens).	Not Applicable	
**Microbes:** provide species and strain, unique accession number if available, and source.	Not Applicable	

Human research participants	Information included in the manuscript?	In which section is the information available? (Reagents and Tools Table, Materials and Methods, Figures, Data Availability Section)
If collected and within the bounds of privacy constraints report on age, sex and gender or ethnicity for all study participants.	Not Applicable	

Core facilities	Information included in the manuscript?	In which section is the information available? (Reagents and Tools Table, Materials and Methods, Figures, Data Availability Section)
If your work benefited from core facilities, was their service mentioned in the acknowledgments section?	Yes	acknowledgements section

Design

Study protocol	Information included in the manuscript?	In which section is the information available? (Reagents and Tools Table, Materials and Methods, Figures, Data Availability Section)
If study protocol has been **pre-registered, provide DOI in the manuscript**. For clinical trials, provide the trial registration number **OR** cite DOI.	Not Applicable	
Report the **clinical trial registration number** (at ClinicalTrials.gov or equivalent), where applicable.	Not Applicable	

Laboratory protocol	Information included in the manuscript?	In which section is the information available? (Reagents and Tools Table, Materials and Methods, Figures, Data Availability Section)
Provide DOI OR other citation details if **external detailed step-by-step protocols** are available.	Not Applicable	

Experimental study design and statistics	Information included in the manuscript?	In which section is the information available? (Reagents and Tools Table, Materials and Methods, Figures, Data Availability Section)
Include a statement about **sample size** estimate even if no statistical methods were used.	Yes	Figure legends
Were any steps taken to minimize the effects of subjective bias when allocating animals/samples to treatment (e.g. **randomization procedure**)? If yes, have they been described?	Yes	Methods
Include a statement about **blinding** even if no blinding was done.	Yes	Methods
Describe **inclusion/exclusion criteria** if samples or animals were excluded from the analysis. Were the criteria pre-established? If sample or data points were omitted from analysis, report if this was due to attrition or intentional exclusion and provide justification.	Yes	Methods
For every figure, are **statistical tests** justified as appropriate? Do the data meet the assumptions of the tests (e.g., normal distribution)? Describe any methods used to assess it. Is there an estimate of variation within each group of data? Is the variance similar between the groups that are being statistically compared?	Yes	Methods, Figure legends

Sample definition and in-laboratory replication	Information included in the manuscript?	In which section is the information available? (Reagents and Tools Table, Materials and Methods, Figures, Data Availability Section)
In the figure legends: state number of times the experiment was **replicated** in laboratory.	Yes	Figure legends
In the figure legends: define whether data describe **technical or biological replicates**.	Yes	Figure legends

Ethics

Ethics	Information included in the manuscript?	In which section is the information available? (Reagents and Tools Table, Materials and Methods, Figures, Data Availability Section)
Studies involving **human participants**: State details of **authority granting ethics approval** (IRB or equivalent committee(s), provide reference number for approval.	Not Applicable	
Studies involving **human participants**: Include a statement confirming that **informed consent** was obtained from all subjects and that the experiments conformed to the principles set out in the WMA Declaration of Helsinki and the Department of Health and Human Services Belmont Report.	Not Applicable	
Studies involving **human participants:** For publication of **patient photos**, include a statement confirming that consent to publish was obtained.	Not Applicable	
Studies involving experimental **animals**: State details of **authority granting ethics approval** (IRB or equivalent committee(s), provide reference number for approval. Include a statement of compliance with ethical regulations.	Yes	Methods
Studies involving **specimen and field samples:** State if relevant **permits** obtained, provide details of authority approving study; if none were required, explain why.	Not Applicable	

Dual Use Research of Concern (DURC)	Information included in the manuscript?	In which section is the information available? (Reagents and Tools Table, Materials and Methods, Figures, Data Availability Section)
Could your study fall under dual use research restrictions? Please check biosecurity documents and list of **select agents and toxins** (CDC): https://www.selectagents.gov/sat/list.htm	Not Applicable	
If you used a select agent, is the security level of the lab appropriate and reported in the manuscript?	Not Applicable	
If a study is subject to dual use research of concern regulations, is the name of the **authority granting approval and reference number** for the regulatory approval provided in the manuscript?	Not Applicable	

Reporting

The MDAR framework recommends adoption of discipline-specific guidelines, established and endorsed through community initiatives. Journals have their own policy about requiring specific guidelines and recommendations to complement MDAR.

Adherence to community standards	Information included in the manuscript?	In which section is the information available? (Reagents and Tools Table, Materials and Methods, Figures, Data Availability Section)
State if relevant guidelines or checklists (e.g., **ICMJE, MIBBI, ARRIVE, PRISMA**) have been followed or provided.	Not Applicable	
For **tumor marker prognostic studies,** we recommend that you follow the **REMARK** reporting guidelines (see link list at top right). See author guidelines, under 'Reporting Guidelines'. Please confirm you have followed these guidelines.	Not Applicable	
For **phase II and III randomized controlled trials**, please refer to the **CONSORT** flow diagram (see link list at top right) and submit the CONSORT checklist (see link list at top right) with your submission. See author guidelines, under 'Reporting Guidelines'. Please confirm you have submitted this list.	Not Applicable	

Data Availability

Data availability	Information included in the manuscript?	In which section is the information available? (Reagents and Tools Table, Materials and Methods, Figures, Data Availability Section)
Have **primary datasets** been deposited according to the journal's guidelines (see 'Data Deposition' section) and the respective accession numbers provided in the Data Availability Section?	Yes	Methods Data Availability
Were **human clinical and genomic datasets** deposited in a public access-controlled repository in accordance to ethical obligations to the patients and to the applicable consent agreement?	Not Applicable	
Are **computational models** that are central and integral to a study available without restrictions in a machine-readable form? Were the relevant accession numbers or links provided?	Not Applicable	
If publicly available data were reused, provide the respective **data citations in the reference list**.	Yes	Methods
