# Supplementary material for: Extracellular RNA clearance by non-canonical cell-surface MDA5 protects against lung fibrosis
Source: EMBO Mol Med. 2026 Oct 12;18(10):4001–33. doi: 10.1038/s44321-026-00513-6 (PMC13658825; doi:10.1038/s44321-026-00513-6)
Supplement: Supplementary file 3 [file 44321_2026_513_MOESM2_ESM.pdf]

GEO Accession	Species	Biomaterial Used	Platform / Methodology	Cohort / Diagnosis Groups	Sample Size (n)	Clinical Characteristics & Demographics Provided in Original Database
GSE135893	Human	Whole lung single-cell suspensions	Single-cell RNA sequencing (scRNA-seq)(Illumina HiSeq 4000)	• Idiopathic Pulmonary Fibrosis (IPF)• Non-Specific Interstitial Pneumonia (NSIP)• Chronic Hypersensitivity Pneumonitis (cHP)• Healthy Controls (Unused donor lungs)	Total: 30 (ILD: 20 Controls: 10) ILD(Idiopathic Pulmonary Fibrosis (IPF; n = 12), chronic hypersensitivity pneumonitis (cHP; n = 3), nonspecific interstitial pneumonia (NSIP; n = 2), sarcoidosis (n = 2), and unclassifiable ILD (n = 1))	Adult human males and females. Metadata includes specific diagnosis, age, sex, race, ethnicity, and smoking history (ever/never smoked).
GSE70866	Human	Bronchoalveolar Lavage Fluid (BALF) cells	Gene expression microarray(Agilent-028004)	• Idiopathic Pulmonary Fibrosis (IPF)• Healthy Donors (HD)	Total: 232 (IPF: 212 Healthy Donors: 20)	Adult human subjects. Metadata includes age, sex, smoking status, pulmonary function test metrics (% predicted FVC, % predicted DLCO), and clinical survival outcomes.
GSE47460	Human	Lung tissue homogenates (LGRC Cohort)	Gene expression microarray(Agilent-028004)	• Idiopathic Pulmonary Fibrosis (IPF)• Non-diseased Controls	Total: 268 (IPF: 160 Controls: 108)	Adult human subjects from the Lung Genomics Research Consortium (LGRC). Metadata includes age, sex, race, smoking history (pack-years), and baseline pulmonary function parameters (% predicted FVC, % predicted DLCO).
GSE128314	Human	Skin tissue biopsies	Gene expression profiling(RNA-seq / Microarray)	• Anti-MDA5 autoantibody-positive Dermatomyositis (DM)• Healthy Controls	Human skin Subjects(Anti-MDA5 autoantibody-positive DM:3 Healthy controls:5)	Adult human subjects presenting with dermatomyositis cutaneous lesions. Metadata includes clinical confirmation of autoantibody profiles (anti-MDA5^{+}) and cutaneous disease severity scores.

Table EV1. Clinical demographics and biomaterial descriptions of publicly available human datasets. This table summarizes the specific biomaterials, experimental platforms, cohort stratifications, sample sizes, and available clinical metadata for the four Gene Expression Omnibus (GEO) datasets re-analyzed in the current study (GSE135893, GSE70866, GSE47460, and GSE128314). All clinical characteristics and patient demographics were extracted directly from the original database repositories to the extent that such data were provided by the primary investigators. *Abbreviations: BALF, bronchoalveolar lavage fluid; cHP, chronic hypersensitivity pneumonitis; DM, dermatomyositis; HD, healthy donor; ILD, interstitial lung disease; IPF, idiopathic pulmonary fibrosis; LGRC, Lung Genomics Research Consortium; NSIP, non-specific interstitial pneumonia; scRNA-seq, single-cell RNA sequencing.*
